# A comparative survey of veterinarians, equine owners, and equine keepers regarding the knowledge and implementation of legal requirements in Germany for the use and documentation of veterinary medicines in equines intended for slaughter

**DOI:** 10.1371/journal.pone.0283371

**Published:** 2023-04-06

**Authors:** Shary Tamara Schneider, Diana Meemken, Heidrun Gehlen, Roswitha Merle, Nina Langkabel

**Affiliations:** 1 Institute for Food Safety and Food Hygiene, Working Group Meat Hygiene, School of Veterinary Medicine, Freie Universität Berlin, Berlin, Germany; 2 Division for Internal Medicine, Equine Clinic, School of Veterinary Medicine, Freie Universität Berlin, Berlin, Germany; 3 Institute for Veterinary Epidemiology and Biostatistics, School of Veterinary Medicine, Freie Universität Berlin, Berlin, Germany; University of Zambia School of Veterinary Medicine, ZAMBIA

## Abstract

In Europe, equines destined for human consumption (hereafter called slaughter equines) are subject to the same restrictions of usage of veterinary drugs as other food-producing animals, with amendments regulated in the so-called ‘positive list’, Regulation (EC) No. 1950/2006. Due to the complex legal requirements for drug administration in slaughter equines, it might be that specific knowledge regarding the legislation of slaughter equines may be insufficient among veterinarians, equine owners, and equine keepers. To study this assumption, three target group-specific surveys were conducted in 2021. Answers from 153 equine treating veterinarians, 170 equine owners, and 70 equine keepers were included in the analysis. In total 68.4% (91/133) of the participating veterinarians, the regulations of the ‘positive list’, Regulation (EC) No. 1950/2006, were ‘rather complicated’ to ‘complicated’. Among the participating veterinarians, 38.4% (58/151) did not or could not answer correctly how to proceed if a slaughter equine is scheduled to receive phenylbutazone, usage of which is prohibited in all livestock by Regulation (EU) No. 37/2010. Simultaneously, 56.2% (86/153) of the participating veterinarians named phenylbutazone as the, or one of the, most often used non-steroidal anti-inflammatory drugs. Altogether, 41.2% (70/170) of participating equine owners and 42.9% (30/70) of equine keepers did not know under which circumstances an equine can legally be slaughtered for human consumption. In total, 34.3% (24/70) of the equine keepers classified their knowledge of national regulations for animal keepers regarding the documentation of drug usage in equines as ‘poor’ to ‘nonexistent’. This lack of knowledge in all three surveyed groups, combined with the complex legal regulations regarding the usage and documentation of drugs in slaughter equines, could result in missing and false documentation, treatment of slaughter equines with prohibited substances and therefore pose a risk factor for drug residues in equine meat.

## 1. Introduction

In 2019 in Germany, around 1.25 million privately owned horses were registered [1] and approximately 454,000 equines were held in agricultural holdings [2]. The number of donkeys living in Germany is estimated at 10,000 to 20,000 by the ‘Interest Group of Donkey and Mule Friends in Germany’ (Interessengemeinschaft der Esel- und Mulifreunde in Deutschland e.V., personal communication). There are no publicly available statistics on the total population of equines in Germany or on what percentage of them is destined for human consumption (meaning they are classified as slaughter equines in their passports).

In 2021 in Germany, a total of 3,489 equines were slaughtered for human consumption and horse-meat consumption was 0.02 kg/per capita [3, 4]. In comparison, the most commonly consumed meat in Germany in 2021 was pork with 42.9 kg/capita [3], and the highest consumption of horse meat in Europe was reported from Italy with 0.88 kg/capita [5]; therefore, equine meat in Germany is a niche product. As the annual number of slaughtered equines in Germany is small, the sample size of equine meat which is analyzed annually for drug residues and contaminants by official controls is also small. In 2020, only 117 samples of equine meat were analyzed for drug and other chemical residues by the federal veterinary offices, while in the same year, 27,928 pork samples and 13,491 beef samples were analyzed [6]. The number of equine meat samples that test positive for drug or chemical residues varies greatly from year to year, which could be explained by the statistically small sample size. From the samples tested annually, 4.51% were positive in 2018 [7], and this decreased to 0.91% in 2019 [8], but then increased up to 1.87% positive samples in 2020 [6]. In contrast, in the same years, the percentages of positive samples for pork were 0.31%, 0.39%, and 0.26% and for beef 0.6%, 0.5%, and 0.79% in 2018, 2019 and 2020 respectively [6–8].

In the European Union, equines can be considered as livestock or as companion animals following legislative definitions. At birth, all equines are considered as livestock i.e., as food-producing animals and, therefore, destined for slaughter. In contrast to other food-producing animals, this status can be changed to companion animal by the owner by documenting it in the individual equine passport; this change requires the signature of the owner and a veterinarian. The equine passport is mandatory for all equines as an official document regarding pedigree, individual marking, vaccination, and drug documentation [9]. With the transition of their status from slaughter equine to companion animals, comes a subsequent change of the applicable European regulations. If an equine receives the status of a companion animal, meaning it is excluded from human consumption, this transition is irreversible.

To ensure adequate medical care for equines destined for human consumption, a list of veterinary medications was compiled, the so-called ‘positive list’ [10]. The listed medications can be used for specific indications in slaughter equines, although they are not approved for other food-producing animals and are not listed in Table 1 of the annex to Regulation (EU) No. 37/2010 [11]. The usage of any of the listed drugs on the ‘positive list’ has to be documented in the equine passport, and a withdrawal period of six months between the last usage of such a drug and the equine’s slaughter must be adhered to. In Germany, the application and dispersion of all other drugs approved for food-producing animals have to be documented in an official form for application and dispersion which is called ‘drug application and dispersion form’ (in German: “Arzneimittelanwendungs- und -abgabe-Beleg”, ‘AuA-Beleg’) according to §13 of the German veterinary pharmacy regulation [12]. According to German legislation, this document has to be issued and signed by the attending veterinarian and given to the equine keeper. The equine keeper has to store the document for five years and must be able to show it during an official inspection [13]. The German legislation differentiates between somebody who owns an equine (equine owner) and somebody who manages a stable and is responsible for keeping an equine (equine keeper). Only the equine keepers are obligated by law to document the administration of medications to equines in their stables [13].

**Table 1:** Contact methods for veterinarians.

Distributor	Contact method
‘German Veterinary Society’ (Deutsche Veterinärmedizinische Gesellschaft, DVG)	Mailing list
‘Society for Equine Medicine’ (Gesellschaft für Pferdemedizin, GPM)	Mailing list
German Veterinary Association of the federal state of Saxony	Mailing list
German Veterinary Association of the federal states Schleswig-Holstein	Mailing list
German Veterinary Association of the federal state of Thuringia	Mailing list
German Veterinary Association of the federal states Baden-Wuerttemberg	Published link to survey on their website
Facebook	Link to seven German veterinarian groups (total number of members at the time of posting: 11,243)
Instagram	Link on private pages of two research group members with approx. 500 followers

The aims of the study were to determine if equine-attending veterinarians, equine owners, and equine keepers have sufficient knowledge about the legal national and European regulations applicable in Germany, and adhere to them, as well as about applicable legal regulations regarding the usage and documentation of drugs in equines destined for slaughter in Germany.

## 2. Materials and methods

### 2.1 Procedure and distribution of the survey

We conducted an online survey among equine-attending veterinarians, equine owners, and equine keepers in Germany. As equines, we included only horses and donkeys, even though zebras and zebra hybrids are equines by definition and the legal regulations in Germany also apply to them. This decision was made because in Germany zebras are usually kept in zoological institutions and are not intended for human consumption.

Data collection took place between 29^th^ October and 15^th^ December 2021. The online survey was created via Lime Survey Community Edition version 3.28.21 (LimeSurvey GmbH–Hamburg, Germany) and hosted on a server of Freie Universität Berlin, Germany.

After following the link to the survey, an explanation of the research project and assurance that the obtained data would be treated confidentially and anonymously according to the current data protection law in Germany was displayed. Additionally, it was made clear that participation was voluntary, that all data would only be used anonymously and confidentially for the sake of the research project, and that no personal data would be collected. Statements assured participants that there was no connection to the official veterinary authorities of the German federal states, and that the collected data would not be given to law enforcement or to anybody else.

Ethical approval for the study was obtained from the Central Ethics Committee of Freie Universität Berlin, Germany under ZEA-Nr. 2022–013.

The participants gave consent to use their data through clicking of the survey start button. The Central Ethics Committee waived the need for additional consent obtaining measures. No personally identifying data was collected. The study was designed for adults only.

As answers for some questions were to clarify facts or were preconditions for further questions, the survey had to be completed in the given order. It was not possible to skip questions, neither forwards nor backwards. Once given, the answers could not be changed. This setting was chosen to minimize cheating on knowledge questions.

#### 2.1.1 Veterinarians´ survey

Veterinarians were contacted via email distribution lists or social media (Table 1).

After opening the link, it was explained on the first page that the target group was all veterinarians who treated equines. Veterinarians who had a main focus of practice on other animals and seldomly treated equines were also encouraged to complete the survey, as were veterinarians who treated only equines.

#### 2.1.2 Equine owners´ and equine keepers´ survey

Equine owners and equine keepers were contacted only via social media by posting the survey links on Instagram and Facebook. For the Facebook postings, 16 German equestrian and donkey groups covering a great variety of equestrian fields and interests were selected, including groups focused on dressage, show jumping, long distance riding, carriage riding, wood logging, pony riding, draft horses, horse breeding, or donkeys. The total number of the Facebook group members was 102,051 at time of posting. A reminder was posted in the groups after two to three weeks.

In the postings, it was explained that there were two different surveys: one for equine owners and one for equine keepers. The definition of equine owner was explained as someone who has the ownership of one or more horses, donkeys, or mules and whose animals were taken care of in a stable that was owned by another person. The definition of equine keeper was explained as someone who owns a stable and takes care of their own or somebody else’s equines. For the equine keeper it did not matter if all or some of the equines kept in their stable were owned by the equine keeper themself or by a different person.

After opening the link to each of these surveys, the difference between equine owners and equine keepers was again explained to ensure that the two surveys were answered by the right target group.

### 2.2 Development of the questionnaires

All questionnaires were developed with the input of the authors and created in German with the Lime Survey software.

A pre-test with seven veterinarians, eleven equine owners, and ten equine keepers was conducted from 19^th^ August to 15^th^ September 2021. The feedback from the pre-test regarding the surveys´ usability was incorporated in the final surveys and the questionnaires were slightly edited. Answers obtained in the pre-test were not included in the final analysis.

#### 2.2.1 Veterinarians´ questionnaire

The veterinarians´ questionnaire consisted of 66 questions (S1 File) with an estimated duration of 15 to 20 minutes. Depending on the answers to specific questions, different or no follow-up questions were displayed. The minimum number of questions that needed to be answered was 32.

Firstly, demographic questions were asked regarding the location of the practice, the animals treated, and whether the participant supervised equine slaughter in abattoirs or butcheries.

Next were specific questions regarding the usage of drugs (especially non-steroidal anti-inflammatory drugs (NSAIDs) like phenylbutazone), and on the documentation procedure. The questionnaire determined if the drug usage and documentation differed depending on the slaughter status of the equine.

The veterinarians were asked about their knowledge of the regulations and legislation covering drug usage and documentation of administered drugs in equines destined for human consumption. Some of the questions were designed as free-text-answer-questions to accurately determine participants’ knowledge.

In the last part of the questionnaire, the veterinarians were asked about the perceived complexity of the regulations and legislation, and whether they would like to have more opportunities to further educate themselves on those topics.

#### 2.2.2 Equine owners´ questionnaire

The equine owners´ questionnaire consisted of 80 questions (S2 File) with an estimated duration of 15 minutes. Depending on the answers to specific questions, different or no follow-up questions were displayed. All displayed questions needed to be answered.

Firstly, the equine owners were asked questions concerning demographics and how many equines they owned. Depending on the answer regarding the number of owned equines, the following questions were designed to fit the given number. General questions about the owned equine(s) were then asked, for example: ‘What breed is/are your equine/s?’

Then, more specific questions about the documentation of administered drugs (via the equine passport and the drug administration and dispersion form) were asked, as well as general knowledge regarding the slaughter status of equines and if they would also send an owned animal to slaughter.

#### 2.2.3 Equine keepers´ questionnaire

The equine keepers´ questionnaire consisted of 42 questions (S3 File) with an estimated duration of 15 minutes. Depending on the answers to specific questions, different or no follow-up questions were displayed. All displayed questions needed to be answered.

Firstly, the equine keepers were asked demographic questions regarding location and type of their stable as well as equine species held. This was followed by specific questions about drug administration and documentation management, including regulations for the drug application and dispersion form.

### 2.3 Data analysis

For veterinarians, 153 records were considered for analysis. Since question No. 32 of questionnaire Q1 (‘Which NSAIDs are prescribed/used most frequently?’; supplementary material Q1) was considered as essential, exclusively datasets of veterinarians who answered at least until question No. 32 were included in the analysis. Due to the integration of incomplete datasets, the reference value changes between results obtained.

Altogether, 170 equine owners and 70 equine keepers completely answered the questionnaires, and these were included in the final analysis. As the equine owners often had more than one equine, we acquired the data for 312 individual equine owners’ animals in total.

Data were analyzed descriptively using and IBM SPSS version 28.0 for Windows (IBM® - Armonk, New York, USA). For some specific questions, the answers of veterinarians, equine owners, and equine keepers were compared between each other in a descriptive way. Frequency tables and figures were configurated.

## 3. Results

### 3.1 Demographic results

In the following a selection of the demographic results for each group are presented. All detailed demographic results for the three participating groups are listed in S1–S3 Tables.

#### 3.1.1 Veterinarians

The reference value changes between the following results due to the integration of incomplete datasets (see 2.3 Data analysis).

The participating veterinarians worked in practices located in 12 of the 16 federal states in Germany (S1 Table). The 153 participating veterinarians owned or were employed in veterinary practices with between one and 45 veterinarians (median = 3.0). Most of the veterinary practices (51.6%; 79/153) had a catchment area with a radius of 50 km or less.

For 68.6% (105/153) of the participating veterinarians, the percentage of equine patients in relation to all treated patients ranged from 75% to 100%. Altogether, 52.3% (80/153) of participating veterinarians stated that the percentage of slaughter equines in relation to all attended equines was less than 10%. However, 2.0% (3/153) answered that between 75% and 100% of their equine patients are slaughter equines, and 4.6% (7/153) of veterinarians stated that they do not know the percentage of equine patients that would be considered as slaughter equines.

While the proportion of mobile practice in total businesses was high, with 64.1% (98/153) of veterinarians having between 75% and 100% of mobile practice, many (37.3%; 57/153) of the veterinarians had facilities to attend to the equines as in-patients. Of those veterinarians, 71.9% (41/57) also had an operating theater for equines.

In total, 4.6% (7/153) of the participating veterinarians worked additionally as official veterinarians in an abattoir or butchery where horses or donkeys were slaughtered. Three of them had attended at least one equine emergency slaughter between 2019 and 2021.

#### 3.1.2 Equine owners

The participating equine owners came from all federal states in Germany (S2 Table). Altogether, 51.8% (88/170) of the equine owners owned one equine at the time of completion of the survey. Two equines were owned by 29.4% (50/170) of owners, 8.2% (14/170) owned three equines, 4.7% (8/170) owned four equines, and 5.9% (10/170) owned five or more equines.

A great variety of different breeds were reported, 87 in total (S2 Table). The three most common breeds were Hanoverian Horse 11.9% (37/312), German Sport Horse (DSP) 5.8% (18/312), and Icelandic Horse 5.8% (18/312). Two of the equines were donkeys. Most equines (80.4%; 251/312) were born in Germany. The oldest equine was 37 years old, the youngest less than one year (n = 310). For two horses, the date of birth was unknown.

The owners were asked to state the slaughter status individually for every equine that they own (as well as if they would hand in an owned equine for slaughter (S1 and S2 Figs), to which 9.4% answered ‘yes’.

#### 3.1.3 Equine keepers

The participating equine keepers had their stables in 11 of the 16 federal states of Germany (S3 Table). The stables of the participating equine keepers were mostly classified as private stable (40.0%; 28/70), or as boarding stable (37.1%; 26/70), followed by 18.6% (13/70) as ‘other’, 15.7% (11/70) as a breeding stable, and 5.7% (4/70) as a horse-riding school.

Selected statistics for the kept equines and their slaughter status can be seen in Table 2.

**Table 2:** Demographic description of kept equines and their slaughter status.

Question	Answers received			
S3F3/F9—Do you care for horses, donkeys, or both?	‘Only horses’ 77.1 % (54/70)	‘Only donkeys’ 5.7 % (4/70)	‘Horses and donkeys’ 17.1 % (12/70)	
S3F3—How many horses do you keep?	‘Less than 10 horses’ 74.2 % (49/66)	‘From 10 to less than 20 horses’ 12.1 % (8/66)	‘From 20 to less than 50 horses’ 12.1 % (8/66)	‘From 50 to 100 horses’ 1.5 % (1/66)
S3F9—How many donkeys do you keep?	‘Less than 10 donkeys’ 93.8 % (15/16)	‘From 10 to less than 20 donkeys’ 6.2 % (1/16)		
S3F4—What percentage of the kept horses is considered for slaughter?	‘<10 %’ 65.2 % (43/66)	‘75 % to ≤100 %’ 10.6 % (7/66)	‘I do not know’ 4.5 % (3/66)	
S3F10—What percentage of the kept donkeys is considered for slaughter?	‘<10 %’ 100 % (16/16)			

In total, 7.1% (5/70) of equine keepers who participated in the survey answered that an emergency slaughter of an equine had been necessary in their stable at least once. This was the fact for four horse keepers and for one donkey keeper. The equine keepers stated that they had one to ten attending veterinarians, with a median of two.

### 3.2 Usage of NSAIDs–veterinarian questionnaire

The veterinarians were asked which NSAIDs are most commonly used and prescribed (more than one answer was allowed). In total, eight different NSAIDs were mentioned: meloxicam (86.3%; 132/153), flunixin (60.1%; 92/153), phenylbutazone (56.2%; 86/153), flunixin-meglumine (26.8%; 41/153), metamizole (11.8%; 18/153), firocoxib (4.6%; 7/153), suxibuzone (4.6%; 7/153), and ketoprofen (1.3%; 2/153).

The veterinarians prescribed or used phenylbutazone four times a month (median; minimum = 0, maximum = 300 times a month, n = 151). Regarding the usage of the most frequently used NSAIDs, 45.4% (69/152) of the veterinarians reported that there was no difference between slaughter equines and companion equines. The question ‘How would you proceed if an equine that is scheduled to receive phenylbutazone is an equine destined for slaughter?’ (correct answers: ‘Change the status of the equine destined for slaughter to not allowed to be slaughtered meaning the horse is a companion animal.’, ‘Use a different drug and document this usage in equine passport or in an ‘AuA-Beleg’.’, and ‘Let the equine owner choose between changing the status of the equine or choosing a different medication with the appropriate documentation.’) were correctly given by 61.6% (93/151) of participating veterinarians. Among the veterinarians, 29.8% (45/151) answered mostly correctly but did forget to mention the adequate documentation; 2.6% (4/151) answered incorrectly. In addition, 6.0% (9/151) did not, or avoided answering the question (S3 Fig). The detailed results are listed in the S4 Table.

### 3.3 Documentation requirements for equines

#### 3.3.1 Veterinarians

In total, 22.0% (33/150) of the veterinarians answered that they know the specifications of the national regulation regarding veterinary drug usage and distribution of medication regarding the use of antibiotics of critical importance (3^rd^ and 4^th^ generation cephalosporins and fluoroquinolones) (TÄHAV, 2018) ‘very well’. Additionally, 47.3% (71/150) stated that they know the specifications ‘well’, 25.3% (38/150) ‘moderately’, 3.3% (5/150) ‘poorly’, and 2.0% (3/150) ‘not at all’.

From the participating veterinarians, 67.2% (90/134) claimed to know the withdrawal period between the last application of a reallocated (Usage that differs from the original drug registration, for example for a different indication.) drug and the slaughter of the animal, while 32.8% (44/134) reported they did not know this withdrawal period. Those 90 veterinarians who stated that they know the withdrawal period were then asked its duration. The correct answer of 28 days was given by 48.9% (44/90), while 1.1% (1/90) answered ‘30 days’, 43.3% (39/90) ‘6 months’ and 6.7% (6/90) did not answer the question. Considering all participating veterinarians, 32.8% (44/134) knew the correct withdrawal period.

Table 3 summarizes the questions and answers regarding the veterinarians’ knowledge of regulations regarding slaughter equines in general as well as of the ‘positive list’.

**Table 3:** Description of veterinarians’ self-assessed knowledge regarding different regulations concerning slaughter equines.

Questions	Answers received				
S4F43—How well do you know the regulations regarding the documentation for equines destined for slaughter?	‘Very well’ 22.7 % (34/150)	‘Well’ 47.3 % (71/150)	‘Moderately’ 22.0 % (33/150)	‘Poorly’ 6.7 % (10/150)	‘I have no knowledge at all’ 1.3 % (2/150)
S4F52—How well do you know the regulations of the ‘positive list’ (Reg. (EC) No. 1950/2006)?	‘Very well’ 8.7 % (13/149)	‘Well’ 38.3 % (57/149)	‘Moderately’ 37.6 % (56/149)	‘Poorly’ 9.4 % (14/149)	‘I have no knowledge at all’ 6.0 % (9/149)
S4F63—How do you perceive the complexity of the regulations regarding the ‘positive list’ (Reg. (EC) No. 1950/2006)?	‘Complicated’ 25.6 % (34/133)	‘Rather complicated’ 42.9 % (57/133)	‘Neither complicated nor simple’ 25.6 % (34/133)	‘Rather simple’ 6.0 % (8/133)	‘Simple’ 0 % (0/133)
S4F62—How do you perceive the overall documentation effort for equines destined for slaughter?	‘Large’ 45.9 % (61/133)	‘Rather large’ 36.8 % (49/133)	‘Moderate’ 13.5 % (18/133)	‘Rather small’ 3.0 % (4/133)	‘Small’ 0.8 % (1/133)

Altogether, 32.2% (48/149) of veterinarians admitted that they did not know the duration of the withdrawal period after administration of a drug on the ‘positive list’. The 67.8% (101/149) of veterinarians who answered that they knew the withdrawal period were then asked to state the correct duration: 82.2% (83/101) answered correctly (6 months), 11.9% (12/101) gave a wrong answer, and 5.9% (6/101) did not answer the question at all. In total among the 149 veterinarians, 83 (55.7%) knew the correct withdrawal period.

In total, 84.8% (106/125) of participating veterinarians answered they would consider uniformly structured equine passports, independent from the breeding associations that issue them, as a simplification. Opportunities for advanced training regarding regulations of drug administration documentation (especially for food-producing animals) was desired by 60.7% (65/107) of the participating veterinarians. The detailed results are listed in the S4 Table.

#### 3.3.2 Equine keepers

Most of the equine keepers perceived their knowledge regarding the documentation of drug usage in equines as ‘moderate’ (27.1%; 19/70) or as ‘good’ (24.3%; 17/70), but 14.3% (10/70) said their knowledge was ‘very good’, while the same number described their knowledge as ‘non-existent’.

Altogether, 70.0% (49/70) of equine keepers claimed that the administration of drugs is documented either by themselves, the attending veterinarian, or the keeper’s employees. In total, 27.1% (19/70) stated that they themselves always document administered drugs, while 52.9% (37/70) reported that they never do this.

The status as slaughter or companion animal was documented for each of the housed equines in the animals’ respective equine passports by 61.4% (43/70) of horse keeping establishments. In total, 42.9% (30/70) of the equine keepers reported that a template for drug usage documentation would be helpful. The detailed results are listed in the S6 Table.

### 3.4 Compared results

In the following the results of the three survey groups for specific questions are compared. All detailed results are listed in the S4–S6 Tables.

#### 3.4.1 Comparison of equine owners and equine keepers of the knowledge regarding the equines´ slaughter status and sources of drug supply

In total, 41.2% (70/170) of all equine owners could not answer correctly under which circumstances an equine is considered for human consumption. Among the self-classified slaughter equine owners, 40.6% (13/32) of these individuals also could not answer this question at all.

This was similar to results for the equine keepers, of whom 42.9% (30/70) did not know under which circumstances an equine is destined for human consumption. For those who only kept horses, 38.9% (21/54) did not know these circumstances, in comparison to 56.3% (9/16) of equine keepers who keep horses and donkeys or only donkeys.

In Table 4, the answers concerning the sources of drug supply for their equines are compared between all equine owners, self-classified slaughter-equine owners, and equine keepers. Over all three groups, the most common source of drug supply was veterinarians, who supplied drugs for equines to between 90.6% (slaughter equine owners) and 100.0% (equine keepers). Almost 40% of all three groups selected online shops as a source of drug supply (38.8% (66/170) of all equine owners, 37.5% (12/32) of slaughter-equine owners, and 40.0% (28/70) of equine keepers).

**Table 4:** Comparison of the drug supply sources of all equine owners, self-classified slaughter-equine owners, and equine keepers.

Answer options	Answer percentage and frequency		
	All equine owners (N = 170)	Slaughter equine owners (N = 32)	Equine keepers (N = 70)
Veterinarian	94.7 % (161/170)	90.6 % (29/32)	100 % (70/70)
Pharmacy	44.7 % (76/170)	53.1 % (17/32)	38.6 % (27/70)
Online shops	38.8 % (66/170)	37.5 % (12/32)	40.0 % (28/70)
Animal healer	8.2 % (14/170)	12.5 % (4/32)	20.0 % (14/70)
Animal chiropractor	1.8 % (3/170)	3.1 % (1/32)	5.7 % (4/70)
Farrier	4.7 % (8/170)	12.5 % (4/32)	7.1 % (5/70)

#### 3.4.2 Comparison of equine passport inspection rates by all three participating groups

While 1.3% (2/150) of veterinarians admitted to never inspecting the equine passport before treating an equine, 5.9% (10/170) of all equine owners, 6.3% (2/32) of the slaughter equine owners, and 10.0% (7/70) of equine keepers reported that their attending veterinarian had never inspected the equine passports.

Concurrently, 5.3% (8/150) of veterinarians answered that they inspect the equine passport before every treatment, whereas 9.4% (16/170) of all equine owners, 12.5% (4/32) of the slaughter equine owners, and 12.9% (9/70) of equine keepers stated the same for their attending veterinarian.

Among the equine keepers, 11.1% (6/54) of the horse keepers and 6.3% (1/16) of the horse and donkey or donkey-only keepers reported that they themselves never inspected the equine passport before housing a new equine.

#### 3.4.3 Comparison of answers concerning the drug application and dispersion form (‘AuA-Belege’) by all three participating groups

Overall, 58.0% (87/150) of veterinarians claimed to ‘always’ issue the drug application and dispersion form for slaughter equines, whereas 11.3% (17/150) stated ‘sometimes’, 17.3% (26/150) ‘only if necessary’, 7.3% (11/150) ‘rarely’, and 6.0% (9/150) ‘never’. When asked for which drugs they issued the drug application and dispersion form, 3.5% (5/141) chose the correct answer (all drugs that do not have to be documented in the equine passport). Among the participating veterinarians, 29.1% (41/141) reported that the equine keeper always received the drug application and dispersion form immediately after application or dispensing, but 10.6% (15/141) stated that ‘the equine owner receives the drug application and dispersion form’.

The official drug application and dispersion form was not known by 59.4% (101/170) of all equine owners, nor by 59.4% (19/32) of the slaughter equine owners. Of the 69 equine owners who did know the drug application and dispersion form, 23 sometimes or always received it from their attending veterinarian (33.3%). Five of the slaughter equine owners sometimes or always received this form from their attending veterinarian (15.6%). Among the owners who receive the drug application and dispersion form, 8.7% (2/23) pass it to their respective equine keeper, and 17.4% (4/23) stated that they would dispose of this form (i.e., they would not store the form).

In total, 57.1% (40/70) of equine keepers knew of the official drug application and dispersion form. Among them, 32.5% (13/40) stated that they do not receive the forms from their attending veterinarian. Of those that receive the drug application and dispersion form, 92.6% (25/27) answered that they store the form. However, just 8.0% (2/25) reported that they store the form for the correct period of five years.

## 4. Discussion

Our survey was conducted to compare the knowledge and practice regarding equines destined for human consumption and documentation requirements in Germany among equine veterinarians, equine owners, and equine keepers. The first critical result of our survey is the high rate of misconception regarding under which circumstances an equine is destined for human consumption, as more than 40% of all equine owners and approximately 40% of the self-classified slaughter equine owners did not know this. This inability of many owners to correctly classify the slaughter status of their equines, and therefore, not be able to inform their equine keeper or attending veterinarian, in turn will lead to wrong or missing documentation of administered drugs. For the equine keepers, the same situation was found, as more than 40% did not know when equines are destined for human consumption. For donkey keepers, this failure rate was even higher, with >50% not knowing the differentiation between slaughter equines and companion equines.

In addition, around 11% of the horse keepers and more than 6% of the horse and donkey or donkey-only keepers admitted that they never inspected the equine passport before housing a new equine, which means these keepers are completely reliant on the information given by the respective equine owner. The equine keepers are legally required to document the administration of drugs for food-producing animals in their stable [13]. It can be assumed that this documentation often is not completed and not stored properly because neither the equine keeper nor the owner know whether the equine in question is classified as slaughter equine in its equine passport. Missing documentation can be a potential risk factor for the safety of equine meat consumers. Without correct and complete documentation, it is impossible to track drug administrations, especially if the equine is sold to a different owner.

The second group of potentially critical results relate to the poor knowledge about applicable laws and regulations regarding drug documentation in equines and the omitted inspections of equine passports within the scope of medical treatment. This poor knowledge can result in a potential consumer risk due to drug residues. In this context, of particular interest are residues of drugs that are forbidden to be administered to slaughter equines (this is any drug not listed in Table 1 of the annex to Reg. (EU) No. 37/2010 resp. in the positive list Reg. (EC) No. 1950/2006 [10, 11]. More than half of the participating veterinarians answered that phenylbutazone (prohibited in livestock) was one of the most frequently used NSAID in their practice. Simultaneously, almost 40% of veterinarians did not or could not answer correctly on how to proceed if a slaughter equine received phenylbutazone. In recent years, a single-digit percentage of horse meat samples analyzed for NSAID residues by the federal veterinary offices in Germany tested positive for phenylbutazone: the failure rate was between 2 and 5% in the years between 2015 and 2018 [7, 14, 15]. Even though the overall risk for consumers due to phenylbutazone residues was classified as low by the European Food Safety Authority [16], the main risks of idiosyncratic blood dyscrasias and genotoxic/carcinogenic potential were reaffirmed [17]. Comparing analytical results for residues in German horse meat with those in horse meat from Mexico, Mexican horse meat contained significantly higher clenbuterol residue rates than German horse meat [18]. Clenbuterol is not a forbidden NSAID in livestock like phenylbutazone is, but maximum residue limits in food products from bovines and equines are set (EC, 2010). In Germany, clenbuterol was not detected in horse meat in the last 20 years [6–8, 14, 15, 19–23], but other chemical contaminants and residues were found in higher amounts than in food products from other animals [6–8]. In 2019 and 2020 and EU wide, the highest proportion of samples tested positive for NSAIDs were derived from horses and their products (>1%), whereas <0.5% of bovine samples and <0.1% of pig and poultry samples were NSAID-positive [16]. Consequently, horse meat poses a higher risk to consumers than meat from other livestock animal species. As the veterinarians in our study did not really know the correct regulations regarding administration and documentation of drug usage in slaughter equines, we conclude that this lack of knowledge in veterinarians will result in a possible risk for drug residues in equine meat. Such knowledge gaps can generally be a risk factor for unlawful behavior, resulting in and associated with grievances in cattle, as shown by Scheibl [24].

Almost 6% of all equine owners, more than 6% of the slaughter equine owners, and 10% of equine keepers reported that their attending veterinarian had never inspected the equine passports of any of their owned or kept equines. Interestingly, only little more than 1% of participating veterinarians admitted they never inspect the equine passport before treating an equine, a great discrepancy with the statements of the equine owners and equine keepers. It is likely that the true equine passport inspection frequency by veterinarians is closer to that reported by equine owners and keepers, who have, unlike the veterinarians themselves, no reason to embellish the true frequency. If we assume the case that unreliable information is given by an equine owner or keeper in respect of an equine’s slaughter status, and the attending veterinarian themself does not inspect the equine passport, the veterinarian cannot know reliably which drugs are allowed to be used for the equine. This could result in an equine destined for human consumption being treated with a drug which is prohibited for usage in food-producing animals; the consequence would be a food safety risk for consumers. It could also result in a companion equine not receiving certain drugs because of the wrongly assumed slaughter status. While the latter scenario does not concern food safety, it could be to the medical disadvantage of the individual animal, resulting in animal welfare or health problems.

A study among pig and cattle farms and pig- and cattle-treating veterinarians showed that records regarding antimicrobial drug usage were incomplete in 9.4% of cases for farms and 3.1% of veterinary practices [25]. This could lead to the assumption that the medication documentation for slaughter equines is at least comparably often incomplete. This assumption is further solidified through our results showing poor knowledge of documentation requirements for slaughter equines, especially regarding the official drug application and dispersion form, as nearly 60% of the self-classified slaughter equine owners and more than 40% of the equine keepers did not know of this form. Altogether, just 3.5% of veterinarians correctly knew which medication for slaughter equines required them to issue the form. In addition, only 55% of the participating veterinarians knew the withdrawal period after administering a drug from the ‘positive list’, which leads to the assumption that documentation of such drugs has a significant likelihood of being incorrect.

The widespread practice of obtaining medication from sources other than a veterinarian must be considered the third problematic result and is critical for consumer safety. Around 40% of the participating equine owners and keepers bought medication from online shops. We were unable to verify if the substances obtained from these online shops were in fact medications or were only substances that pose as medication (i.e., feed supplements or chemical substances), and if they were medications, if they were approved and registered for animals, specifically for food-producing animals. The same applies to the information that some equine owners and equine keepers buy what they perceive as medication from animal healers, farriers, and animal chiropractors. These three occupational groups do not have any legal right to disperse veterinary drugs in Germany. This practice of obtaining medication for equines through sources other than an attending veterinarian is not limited to German equine owners and keepers, as a study from Denmark reported that 15.8% of horse owners bought anthelmintics from pharmacies, 3.3% over the internet, and 2.6% from other sources [26]. While this proportion of Danish horse owners is significantly lower than for the German owners in our study, the Danish study takes only anthelmintics into account; nonetheless, a tendency to use other, non-veterinary sources for obtaining medications, like in our study, can be seen.

Lastly, equine-attending veterinarians and equine keepers are willing to optimize their documentation practice, if given access to easy-to-use and standardized documentation templates. Almost 85% of veterinarians consider a uniformly structured equine passport independent from the breeding associations that issue them, a desirable simplification, and more than 40% of equine keepers perceive that a template for drug usage documentation would be helpful. Standardization of training and documentation material was generally perceived as helpful in a study among pig and cattle abattoirs, where 72.4% of the participants would prefer standardized material regarding animal welfare over their individually implemented material [27].

In summary, after interpreting the obtained results, we conclude that the average equine-attending veterinarian, equine owner, and equine keeper do not have sufficient knowledge about the legal German and European regulations applicable for drug usage in equines in Germany and, therefore, do not adhere to them.

Our results based only on an online survey, meaning veterinarians, equine owners, and equine keepers without internet access could not be included in the study, so a selection bias has to be acknowledged, even though internet access is widely available in Germany. Furthermore, it is likely that mostly equine owners and equine keepers with a heightened interest in equine medication completed the survey. However, to the best of our knowledge, this study is the first one in Germany comparing the knowledge of equine veterinarians, equine owners, and equine keepers regarding requirements and practices of administration and documentation of drugs in equines and slaughter equines. Even though there are limitations in our study, we assume that the tendencies of a serious lack of knowledge, of missing documentation, and of associated challenges can be generalized for Germany.

## 5. Conclusion

From our survey results, it can be deduced that a lack of knowledge exists among veterinarians, equine owners, and equine keepers in Germany. The main knowledge gaps concern the national applicable regulations regarding drug usage and documentation in equines as well as under which circumstances an equine can legally be slaughtered for human consumption. This lack of knowledge results in a potential high risk of drug residues in equine meat. To mitigate this fact, more educational opportunities need to be implemented for veterinarians, equine owners, and equine keepers. Uniform standardized templates need to be created and distributed for the documentation of drug usage in all equines and especially in slaughter equines.

## Supporting information

S1 Fig Answers of the participating owners for each individual owned horse (N = 312) regarding the equines´ slaughter status (questions S2F69-F73). (TIF)

S2 Fig Answers of the participating owners (N = 170) regarding hypothetical decision to hand in their own equine(s) for slaughter (question S2F74). (TIF)

S3 Fig Answers received from participating veterinarians (N = 151) on the procedure regarding usage of phenylbutazone and documentation in slaughter equines (question S4F35). (TIF)

S1 Table Demographic questions–veterinarians. (DOCX)

S2 Table Demographic questions–equine owners. (DOCX)

S3 Table Demographic questions–equine keepers. (DOCX)

S4 Table Specific questions–veterinarians. (DOCX)

S5 Table Specific questions–equine owners. (DOCX)

S6 Table Specific questions–equine keepers. (DOCX)

S1 File Veterinarian´s questionnaire. (PDF)

S2 File Equine owners´ questionnaire. (PDF)

S3 File Equine keepers´ questionnaire. (PDF)
