# Supplementary figures and images for: A comparative survey of veterinarians, equine owners, and equine keepers regarding the knowledge and implementation of legal requirements in Germany for the use and documentation of veterinary medicines in equines intended for slaughter
Source: PLoS One. 2023 Apr 6;18(4):e0283371. doi: 10.1371/journal.pone.0283371 (PMC10079036; doi:10.1371/journal.pone.0283371)

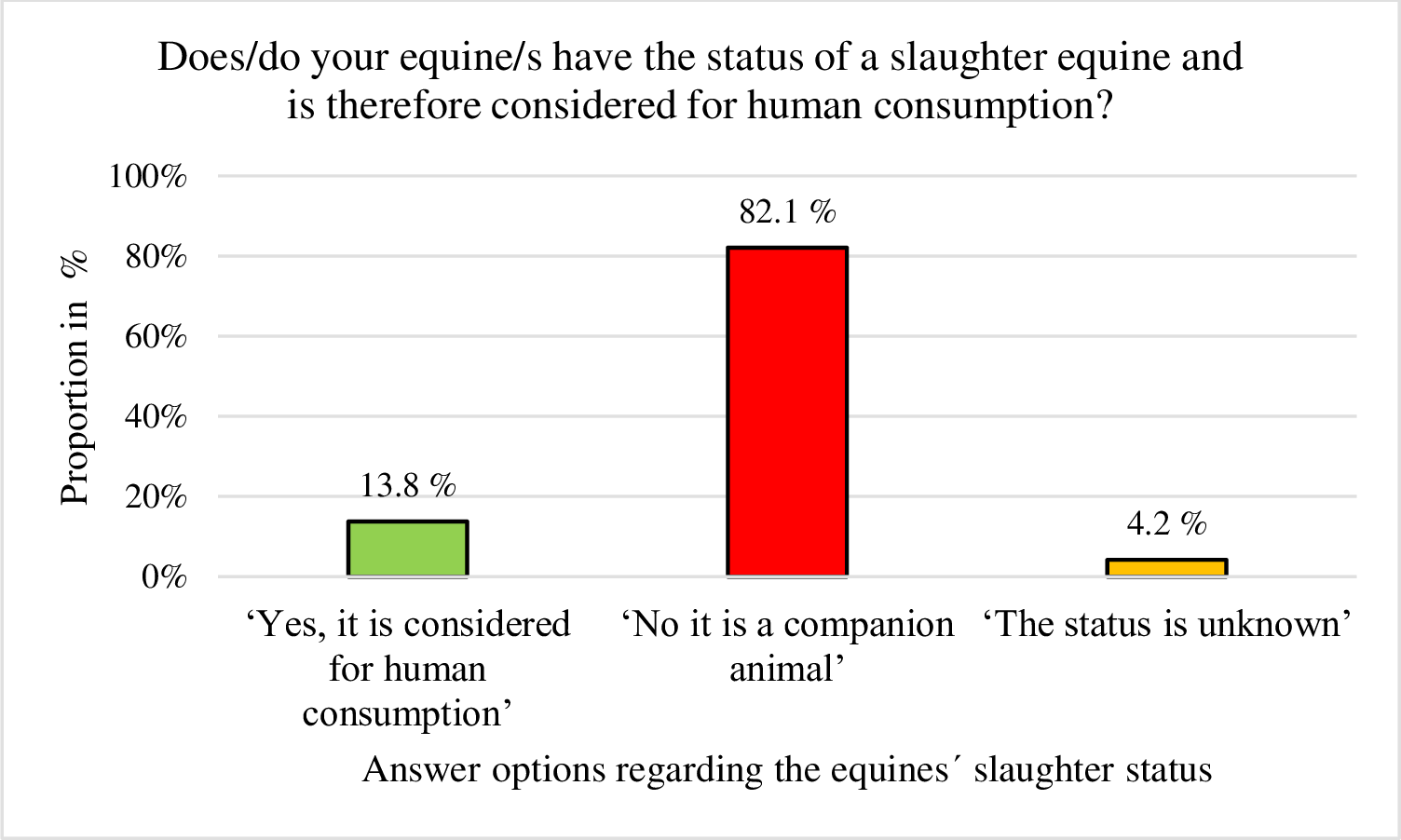

Supplement: S1 Fig — (TIF) [file pone.0283371.s001.tif]

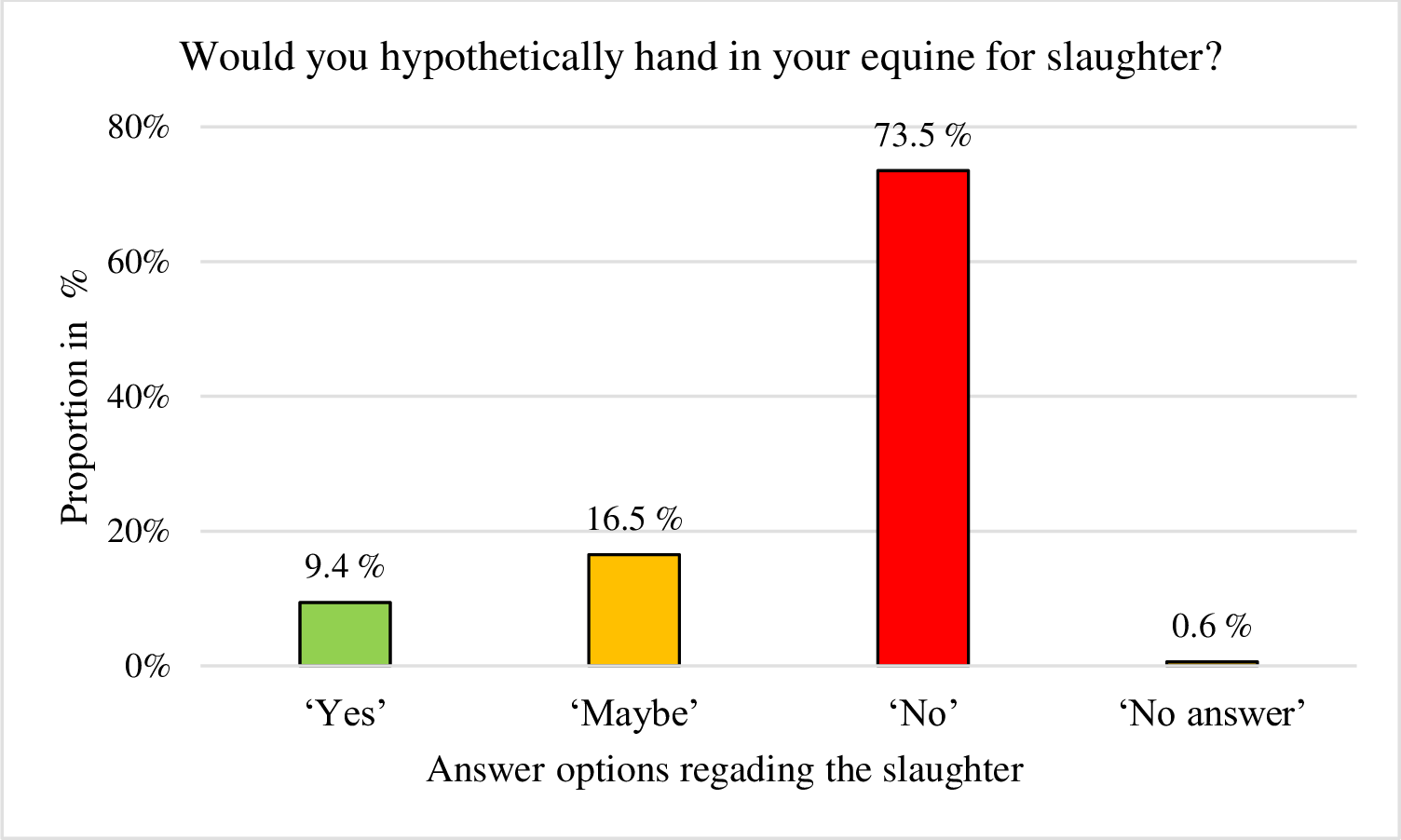

Supplement: S2 Fig — (TIF) [file pone.0283371.s002.tif]

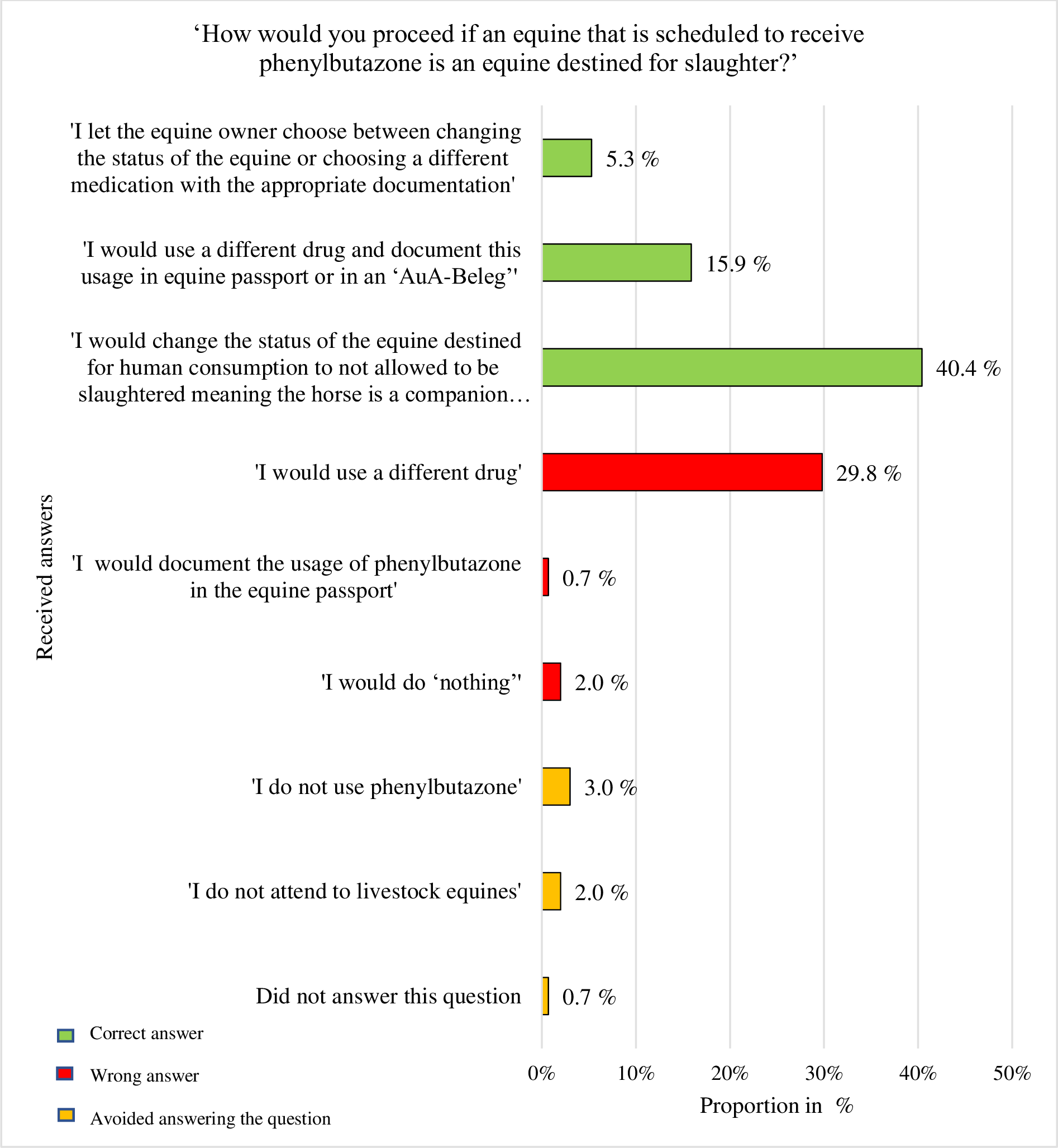

Supplement: S3 Fig — (TIF) [file pone.0283371.s003.tif]
