# Supplementary material for: A comparative survey of veterinarians, equine owners, and equine keepers regarding the knowledge and implementation of legal requirements in Germany for the use and documentation of veterinary medicines in equines intended for slaughter
Source: PLoS One. 2023 Apr 6;18(4):e0283371. doi: 10.1371/journal.pone.0283371 (PMC10079036; doi:10.1371/journal.pone.0283371)
Supplement: S1 Table — (DOCX) [file pone.0283371.s004.docx]

**Table S 1: Demographic questions - Veterinarians**

| **F1 ‘In which federal state is your practice located?’** | | |
| --- | --- | --- |
| **Answer options** | **No. of answers** | **Answer percentage** |
| ‘Baden-Wuerttemberg’ | 13 | 8.4 |
| ‘Bavaria’ | 24 | 15.6 |
| ‘Berlin’ | 1 | 0.6 |
| ‘Brandenburg’ | 15 | 9.7 |
| ‘Bremen’ | 0 | 0.0 |
| ‘Hamburg’ | 0 | 0.0 |
| ‘Hessia’ | 8 | 5.2 |
| ‘Mecklenburg-Western Pomerania’ | 0 | 0.0 |
| ‘Lower Saxony’ | 30 | 19.5 |
| ‘North Rhine-Westphalia’ | 22 | 14.3 |
| ‘Rhineland-Palatinate’ | 6 | 3.9 |
| ‘Saarland’ | 2 | 1.3 |
| ‘Saxony-Anhalt’ | 0 | 0.0 |
| ‘Saxony’ | 4 | 2.6 |
| ‘Schleswig-Holstein’ | 27 | 17.5 |
| ‘Thuringia’ | 2 | 1.3 |
| Total | 153 | 100.0 |
|  | | |
| **F2 ‘How many veterinarians are employed in the veterinary practice you own or are employed in?’** | | |
| N | 153 | |
| Mean | 4.892 | |
| Median | 3.000 | |
| Standard deviation | 5.9297 | |
| Span | 44.0 | |
| Minimum | 1.0 | |
| Maximum | 45.0 | |
|  | | |
| **F3 ‘What is the catchment area of your practice?’** | | |
| **Answer options** | **No. of answers** | **Answer percentage** |
| ‘20 km or less’ | 5 | 3.3 |
| ‘50 km or less’ | 79 | 51.6 |
| ‘75 km or less’ | 22 | 14.4 |
| ‘100 km or less’ | 27 | 17.6 |
| ‘More than 100 km’ | 20 | 13.1 |
| Total | 153 | 100.0 |
|  | | |
| **F4 ‘What is the estimated percentage of equine patients (horses and donkeys) in relation to all treated patients in your practice?’** | | |
| **Answer options** | **No. of answers** | **Answer percentage** |
| ‘<10%’ | 4 | 2.6 |
| ‘10 to <25%’ | 7 | 4.6 |
| ‘25 to <50%’ | 22 | 14.4 |
| ‘50 to <75%’ | 15 | 9.8 |
| ‘75 to ≤100%’ | 105 | 68.6 |
| Total | 153 | 100.0 |

| **F5 ‘What percentage of your equine patients are livestock equines and destined for slaughter?’** | | |
| --- | --- | --- |
| **Answer options** | **No. of answers** | **Answer percentage** |
| ‘<10%’ | 80 | 52.3 |
| ‘10 to <25%’ | 44 | 28.8 |
| ‘25 to <50%’ | 14 | 9.2 |
| ‘50 to <75%’ | 5 | 3.3 |
| ‘75 to ≤100%’ | 3 | 2.0 |
| ‘I do not know’ | 7 | 4.6 |
| Total | 153 | 100.0 |
|  | | |
| **F6 ‘What is the share of mobile practice in total business?’** | | |
| **Answer options** | **No. of answers** | **Answer percentage** |
| ‘<10%’ | 9 | 5.9 |
| ‘10 to <25%’ | 11 | 7.2 |
| ‘25 to <50%’ | 12 | 7.8 |
| ‘50 to <75%’ | 23 | 15.0 |
| ‘75 to ≤100%’ | 98 | 64.1 |
| Total | 153 | 100.0 |
|  | | |
| **F7 ‘Do you have the means to treat equines in patient?’** | | |
| **Answer options** | **No. of answers** | **Answer percentage** |
| ‘Yes’ | 57 | 37.3 |
| ‘No’ | 96 | 62.7 |
| Total | 153 | 100.0 |
|  | | |
| **F8 ‘Do you have an operating theater for equines?’** | | |
| **Answer options** | **No. of answers** | **Answer percentage** |
| ‘Yes’ | 41 | 71.9 |
| ‘No’ | 16 | 28.1 |
| Total | 57 | 100.0 |
|  | | |
| **F9 ‘Do you also work as official veterinarian in an abattoir resp. butchery where horses or donkeys are slaughtered?’** | | |
| **Answer options** | **No. of answers** | **Answer percentage** |
| ‘Yes’ | 7 | 4.6 |
| ‘No’ | 146 | 95.4 |
| Total | 153 | 100.0 |
|  | | |
| **F10 ‘Have you conducted an official ante-mortem inspection as part of an emergency equine slaughter since 2019?’** | | |
| **Answer options** | **No. of answers** | **Answer percentage** |
| ‘Yes’ | 3 | 42.9 |
| ‘No’ | 4 | 57.1 |
| Total | 7 | 100.0 |

**F** = Questions from the Questionnaires

The numeration and order of the tables follows the numeration and the order of the questions displayed in the questionnaires.

The gaps in the numeration result from the fact that data from questions that are not discussed in the study are not shown here.
