# Supplementary material for: A comparative survey of veterinarians, equine owners, and equine keepers regarding the knowledge and implementation of legal requirements in Germany for the use and documentation of veterinary medicines in equines intended for slaughter
Source: PLoS One. 2023 Apr 6;18(4):e0283371. doi: 10.1371/journal.pone.0283371 (PMC10079036; doi:10.1371/journal.pone.0283371)
Supplement: S2 Table — (DOCX) [file pone.0283371.s005.docx]

**Table S 2: Demographic questions – Equine owners**

| **F1 ‘In which federal state is/are your horse(s) kept?’** | | |
| --- | --- | --- |
| **Answer options** | **No. of answers** | **Percentage of answers** |
| ‘Baden-Wuerttemberg’ | 69 | 40.6 |
| ‘Bavaria’ | 18 | 10.6 |
| ‘Berlin’ | 3 | 1.8 |
| ‘Brandenburg’ | 14 | 8.2 |
| ‘Bremen’ | 1 | 0.6 |
| ‘Hamburg’ | 1 | 0.6 |
| ‘Hessia’ | 8 | 4.7 |
| ‘Mecklenburg-Western Pomerania’ | 1 | 0.6 |
| ‘Lower Saxony’ | 20 | 11.8 |
| ‘North Rhine-Westphalia’ | 18 | 10.6 |
| ‘Rhineland-Palatinate’ | 3 | 1.8 |
| ‘Saarland’ | 2 | 1.2 |
| ‘Saxony-Anhalt’ | 6 | 3.5 |
| ‘Saxony’ | 1 | 0.6 |
| ‘Schleswig-Holstein’ | 3 | 1.8 |
| ‘Thuringia’ | 2 | 1.2 |
| Total | 170 | 100.0 |
|  | | |
| **F2 ‘How many equines do you own?’** | | |
| **Answer option** | **No. of answers** | **Answer percentage** |
| ‘1 equine’ | 88 | 51.8 |
| ‘2 equines’ | 50 | 29.4 |
| ‘3 equines’ | 14 | 8.2 |
| ‘4 equines’ | 8 | 4.7 |
| ‘5 equines or more’ | 10 | 5.9 |
|  | | |
| **F3-F7 ‘In which year was/were your equine/s born?’** | | |
| N | 310 | |
| Mean | 2007,84 | |
| Standard Deviation | 7,302 | |
| Span | 37 | |
| Minimum | 1984 | |
| Maximum | 2021 | |
|  | | |
| **F8-F12 ‘What breed is/are your equine/s?’** | | |
| **Given answers** | **No. of answers** | **Answer percentage** |
| American Quarter Horse | 1 | 0.3 |
| Anglo Arabian Horse | 1 | 0.3 |
| Appaloosa-Mix | 1 | 0.3 |
| Arabian Horse | 1 | 0.3 |
| Arabian-Friesian-Mix | 1 | 0.3 |
| Arabian-Lipizzan-Mix | 1 | 0.3 |
| Austrian Horse | 1 | 0.3 |
| Austrian Warmblood | 1 | 0.3 |
| Bardigiano | 1 | 0.3 |
| Bavarian Warmblood | 6 | 1.9 |
| Belgian Warmblood | 1 | 0.3 |
| Black Forest Horse | 2 | 0.6 |
| Brandenburg Warmblood | 1 | 0.3 |
| Classic Pony | 1 | 0.3 |
| Coldblood-Friesian-Mix | 1 | 0.3 |
| Cold blood-German Riding Pony-Mix | 1 | 0.3 |
| Connemara | 1 | 0.3 |
| Cruzado | 1 | 0.3 |
| Donkey | 1 | 0.3 |
| Dutch Horse | 2 | 0.6 |
| European Large Donkey | 1 | 0.3 |
| English Thoroughbred | 1 | 0.3 |
| English Thouroughbred-Trakehner-Mix | 1 | 0.3 |
| Freiberger | 2 | 0.6 |
| Frisian | 1 | 0.3 |
| German Horse | 2 | 0.6 |
| German Riding Horse | 3 | 1.0 |
| German Riding Pony | 9 | 2.9 |
| German Sport Horse | 18 | 5.8 |
| Haflinger | 7 | 2.2 |
| Haflinger Mix | 1 | 0.3 |
| Hanoverian | 37 | 11.9 |
| Hanoverian-Mix | 1 | 0.3 |
| Holsteiner | 17 | 5.4 |
| Hungarian Warmblood | 2 | 0.6 |
| Icelandic Horse | 18 | 5.8 |
| Icelandic Horse-Mix | 1 | 0.3 |
| Irish Tinker | 1 | 0.3 |
| Knabstrupper | 2 | 0.6 |
| Lewitzer | 4 | 1.3 |
| Lusitano | 2 | 0.6 |
| Mecklenburg Horse | 1 | 0.3 |
| Mix | 1 | 0.3 |
| Moritzburg Horse | 2 | 0.6 |
| Noriker | 2 | 0.6 |
| Oldenburg Horse | 11 | 3.5 |
| Oldenburg Show Jumper | 3 | 1.0 |
| Paint Horse | 1 | 0.3 |
| Pintabian Thoroughbred Arabian Horse | 1 | 0.3 |
| Pinto-Hunter-Mix | 1 | 0.3 |
| Polish Coldblood-Warmblood-Mix | 1 | 0.3 |
| Polish Halfbreed | 2 | 0.6 |
| Polish Horse | 1 | 0.3 |
| Polish Warmblood | 1 | 0.3 |
| Pony | 3 | 1.0 |
| Pony Mix | 1 | 0.3 |
| Quarab | 1 | 0.3 |
| Quarter Horse | 4 | 1.3 |
| Quarter Pony | 1 | 0.3 |
| Rhinelander Horse | 5 | 1.6 |
| Rhineland Pony | 1 | 0.3 |
| Royal Dutch Warmblood Horse | 5 | 1.6 |
| Saxon-Anhaltian Horse | 2 | 0.6 |
| Saxon Thuringian Heavy Warmblood | 1 | 0.3 |
| Saxon Thuringian Horse | 2 | 0.6 |
| Shetland Pony | 10 | 3.2 |
| Shire Horse-Noriker-Mix | 1 | 0.3 |
| Spanish Purebred | 9 | 2.9 |
| Swiss Cream Color | 1 | 0.3 |
| Swiss Warmblood | 1 | 0.3 |
| Thoroughbred | 1 | 0.3 |
| Thoroughbred-Arabian-Mix | 2 | 0.6 |
| Tinker | 5 | 1.6 |
| Tinker-Arabian-Mix | 1 | 0.3 |
| Trakehner | 6 | 1.9 |
| Trotter | 4 | 1.3 |
| Warmblood | 17 | 5.4 |
| Welsh A | 3 | 1.0 |
| Welsh Cob | 3 | 1.0 |
| Welshpony-Percheron mix | 1 | 0.3 |
| Westfalian Warmblood | 14 | 4.5 |
| Westfalian Warmblood-German Riding Pony-Mix | 1 | 0.3 |
| Württemberger | 13 | 4.2 |
| Zweibrücker | 3 | 1 |
| Not specified | 4 | 1.3 |
| Total | 312 | 100.0 |
|  | | |
| **F23-F27 ‘In which country was/were your equine/s born?’** | | |
| **Given answers** | **No. of answers** | **Answer percentage** |
| ‘Austria’ | 11 | 3.5 |
| ‘Belgium’ | 1 | 0.3 |
| ‘England’ | 2 | 0.6 |
| ‘France’ | 3 | 1.0 |
| ‘Germany’ | 251 | 80.4 |
| ‘Hungary’ | 3 | 1.0 |
| ‘Ireland’ | 3 | 1.0 |
| ‘Iceland’ | 1 | 0.3 |
| ‘Italy’ | 1 | 0.3 |
| ‘Poland’ | 7 | 2.2 |
| ‘Switzerland’ | 3 | 1.0 |
| ‘Spain’ | 9 | 2.9 |
| ‘The Netherlands’ | 12 | 3.8 |
| ‘Unknown’ | 1 | 0.3 |
| No answer | 4 | 1.3 |
| Total | 312 | 100.0 |
|  | | |
| **F69-F73 ‘Does/do your equine/s have the status of a slaughter equine and is/are therefore**  **considered for human consumption?’** | | |
| **Answer options** | **No. of answers** | **Answer percentage** |
| ‘Yes, it is considered for slaughter.’ | 43 | 13.8 |
| ‘No, it is a companion animal.’ | 256 | 82.1 |
| ‘The status is unknown.’ | 13 | 4.2 |
| Total | 312 | 100.0 |
|  | | |
| **F74 ‘Would you hypothetically hand in your equine(s) for slaughter?’** | | |
| **Answer options** | **No. of answers** | **Answer percentage** |
| ‘Yes’ | 16 | 9.4 |
| ‘Maybe’ | 28 | 16.5 |
| ‘No’ | 125 | 73.5 |
| ‘No answer’ | 1 | 0.6 |
| Total | 170 | 100.0 |

**F** = Questions from the Questionnaires

The numeration and order of the tables follows the numeration and the order of the questions displayed in the questionnaires.

The gaps in the numeration result from the fact that data from questions that are not discussed in the study are not shown here.
