# Supplementary material for: A comparative survey of veterinarians, equine owners, and equine keepers regarding the knowledge and implementation of legal requirements in Germany for the use and documentation of veterinary medicines in equines intended for slaughter
Source: PLoS One. 2023 Apr 6;18(4):e0283371. doi: 10.1371/journal.pone.0283371 (PMC10079036; doi:10.1371/journal.pone.0283371)
Supplement: S3 Table — (DOCX) [file pone.0283371.s006.docx]

**Table S 3:** **Demographic questions – Equine keepers**

| F1 ‘In which federal state is your stable located?’ | | |
| --- | --- | --- |
| Answer options | **No. of answers** | **Answer percentage** |
| ‘Baden-Wuerttemberg’ | 25 | 35.7 |
| ‘Bavaria’ | 9 | 12.9 |
| ‘Berlin’ | 0 | 0.0 |
| ‘Brandenburg’ | 2 | 2.9 |
| ‘Bremen’ | 0 | 0.0 |
| ‘Hamburg’ | 0 | 0.0 |
| ‘Hessia’ | 2 | 2.9 |
| ‘Mecklenburg-Western Pomerania’ | 1 | 1.4 |
| ‘Lower Saxony’ | 7 | 10.0 |
| ‘North Rhine-Westphalia’ | 12 | 17.1 |
| ‘Rhineland-Palatinate’ | 1 | 1.4 |
| ‘Saarland’ | 0 | 0.0 |
| ‘Saxony-Anhalt’ | 6 | 8.6 |
| ‘Saxony’ | 2 | 2.9 |
| ‘Schleswig-Holstein’ | 3 | 4.3 |
| ‘Thuringia’ | 0 | 0.0 |
| Total | 70 | 100.0 |
|  | | |
| F2 ‘What kind of horse- or donkey-keeping stable do you operate?’; Multiple answers could be given; N = 70 | | |
| Given answers | **No. of answers** | **Answer percentage** |
| ‘Privat stable*’ | 28 | 40.0 |
| ‘Boarding stable’ | 26 | 37.1 |
| ‘Breeding stable’ | 11 | 15.7 |
| ‘Horse-riding school’ | 4 | 5.7 |
| ‘Mare milk farm’ | 0 | 0.0 |
| ‘Other’ | 13 | 18.6 |
| *most frequent answer explained in the “other” comment field | | |
|  | | |
| F3 ‘How many horses do you keep?’ | | |
| Answer options | **No. of answers** | **Answer percentage** |
| ‘Less than 10 horses’ | 49 | 74.2 |
| ‘From 10 to less than 20 horses’ | 8 | 12.1 |
| ‘From 20 to less than 50 horses’ | 8 | 12.1 |
| ‘From 50 to 100 horses’ | 1 | 1.5 |
| More than 100 horses | 0 | 0.0 |
| Total | 66 | 100.0 |
|  | | |
| F3/F9* ‘Do you care for horses, donkeys, or both?’ | | |
| Answer options | **No. of answers** | **Answer percentage** |
| ‘Only horses’ | 54 | 77.1 |
| ‘Only donkeys’ | 4 | 5.7 |
| ‘Horses and donkeys’ | 12 | 17.1 |
| Total | 70 | 100.0 |
| *calculated from questions F3 and F9 | | |

| F4 ‘What percentage of the kept horses is considered for slaughter?’ | | |
| --- | --- | --- |
| Answer options | **No. of answers** | **Answer percentage** |
| ‘<10%’ | 43 | 65.2 |
| ‘10% to <25%’ | 7 | 10.6 |
| ‘25% to <50%’ | 4 | 6.1 |
| ‘50% to <75%’ | 2 | 3.0 |
| ‘75% to ≤100%’ | 7 | 10.6 |
| ‘I do not know’ | 3 | 4.5 |
| Total | 66 | 100.0 |
|  | | |
| F9 ‘How many donkeys do you keep?’ | | |
| Answer options | **No. of answers** | **Answer percentage** |
| ‘Less than 10 donkeys’ | 15 | 93.8 |
| ‘From 10 to less than 20 donkeys’ | 1 | 6.2 |
| ‘From 20 to less than 50 donkeys’ | 0 | 0.0 |
| ‘From 50 to 100 donkeys’ | 0 | 0.0 |
| ‘More than 100 donkeys’ | 0 | 0.0 |
| Total | 16 | 100.0 |
|  | | |
| F10 ‘What percentage of the kept donkeys is considered for slaughter?’ | | |
| Answer options | **No. of answers** | **Answer percentage** |
| ‘<10%’ | 16 | 100.0 |
| ‘10% to <25%’ | 0 | 0.0 |
| ‘25% to <50%’ | 0 | 0.0 |
| ‘50% to <75%’ | 0 | 0.0 |
| ‘75% to ≤100%’ | 0 | 0.0 |
| ‘I do not know’ | 0 | 0.0 |
| Total | 16 | 100.0 |
|  | | |
| F8/F14 ‘Was it ever necessary to perform an emergency slaughter of a horse/donkey in your stable?’ | | |
| Answer options | **No. of answers** | **Answer percentage** |
| ‘Yes’ | 4 | 6.1 |
| ‘No’ | 61 | 92.4 |
| ‘No answer’ | 1 | 1.5 |
| Total | 66 | 100.0 |
|  | | |
| F15 ‘How many veterinarians attend to animals in your stable?’ | | |
| N | 70 | |
| Mean | 2.34 | |
| Median | 2.00 | |
| Standard Deviation | 1.453 | |
| Span | 9 | |
| Minimum | 1 | |
| Maximum | 10 | |

**F** = Questions from the Questionnaires

The numeration and order of the tables follows the numeration and the order of the questions displayed in the questionnaires.

The gaps in the numeration result from the fact that data from questions that are not discussed in the study are not shown here.
