# Supplementary material for: A comparative survey of veterinarians, equine owners, and equine keepers regarding the knowledge and implementation of legal requirements in Germany for the use and documentation of veterinary medicines in equines intended for slaughter
Source: PLoS One. 2023 Apr 6;18(4):e0283371. doi: 10.1371/journal.pone.0283371 (PMC10079036; doi:10.1371/journal.pone.0283371)
Supplement: S4 Table — (DOCX) [file pone.0283371.s007.docx]

**Table S 4: Specific questions – Veterinarians**

| **F32 ‘What NSAID(s) do you most frequently use/disperse/prescribe?’;**  **Multiple answers could be given. No answer options were predefined. N = 153** | | |
| --- | --- | --- |
| **Given answers** | **No. of answers** | **Answer percentage** |
| ‘Firocoxib’ | 7 | 4.6 |
| ‘Flunixine’ | 92 | 60.1 |
| ‘Flunixine-Meglumine’ | 41 | 26.8 |
| ‘Ketoprofen’ | 2 | 1.3 |
| ‘Meloxicam’ | 132 | 86.3 |
| ‘Metamizole’ | 18 | 11.8 |
| ‘Phenylbutazone’ | 86 | 56.2 |
| ‘Suxibuzone’ | 7 | 4.6 |
|  | | |
| **F33 ‘Are there differences in the most commonly prescribed/used NSAIDs between slaughter equines and companion equines?’** | | |
| **Answer options** | **No. of answers** | **Answer percentage** |
| ‘Yes’ | 83 | 54.6 |
| ‘No’ | 69 | 45.4 |
| Total | 152 | 100.0 |
|  | | |
| **F34 ‘How often do you prescribe or use phenylbutazone in a horse/donkey on average in one month?’** | | |
| N | 151 | |
| Mean | 12.444 | |
| Median | 4.000 | |
| Standard Deviation | 29.4064 | |
| Span | 300.0 | |
| Minimum | 0 | |
| Maximum | 300.0 | |

| **F35 ‘How would you proceed if an equine that is scheduled to receive phenylbutazone is an equine destined for slaughter?’** | | | | | |
| --- | --- | --- | --- | --- | --- |
| **Answer options** | **No. of answers** | | **Answer percentage** | | **Classification of answer** |
| ‘Do nothing. ’ | 3 | | 2.0 | | wrong |
| ‘Document the usage of phenylbutazone in the equine passport. ’ | 1 | | 0.7 | | wrong |
| ‘Change the status of the equine destined for slaughter to not allowed to be slaughtered meaning the horse is a companion animal. ’ | 61 | | 40.4 | | correct |
| ‘Use a different drug. ’ | 45 | | 29.8 | | wrong answer, since no documentation was mentioned |
| ‘Use a different drug and document this usage in equine passport or in an ‘AuA-Beleg’. ’ | 24 | | 15.9 | | correct |
| ‘Let the equine owner choose between changing the status of the equine or choosing a different medication with the appropriate documentation. ’ | 8 | | 5.3 | | correct |
| ‘Other’ (specified through the answering veterinarians): I do not use phenylbutazone | 5 | | 3.3 | | avoided answering the question |
| ‘Other’ (specified through the answering veterinarians): I do not attend to livestock equines. | 3 | | 2.0 | | avoided answering the question |
| No answer. | 1 | | 0.7 | | No answer |
| Total | 151 | | 100.0 | |  |
|  | | | | | |
| **F41 ‘How well do you know the specifications of the national regulation regarding veterinary drug usage and distribution of medication (TÄHAV, 2018)** **regarding the use of antibiotics of critical importance?’** | | | | | |
| **Answer options** | | **No. of answers** | | **Answer percentage** | |
| ‘Very well’ | | 33 | | 22.0 | |
| ‘Well’ | | 71 | | 47.3 | |
| ‘Moderately’ | | 38 | | 25.3 | |
| ‘Poorly’ | | 5 | | 3.3 | |
| ‘I have no knowledge’ | | 3 | | 2.0 | |
| Total | | 150 | | 100.0 | |
|  | | | | | |
| **F43 ‘How well do you know the regulations regarding the documentation for equines destined for slaughter?’** | | | | | |
| **Answer options** | | **No. of answers** | | **Answer percentage** | |
| ‘Very well’ | | 34 | | 22.7 | |
| ‘Well’ | | 71 | | 47.3 | |
| ‘Moderately’ | | 33 | | 22.0 | |
| ‘Poorly’ | | 10 | | 6.7 | |
| ‘I have no knowledge’ | | 2 | | 1.3 | |
| Total | | 150 | | 100.0 | |

| **F44 ‘When do you inspect the equine passport before treating an equine?’** | | |
| --- | --- | --- |
| **Given answers** | **No. of answers** | **Answer percentage** |
| ‘Never’ | 2 | 1.3 |
| ‘Once before first patient admission’ | 85 | 56.7 |
| ‘Before major procedures’ | 20 | 13.3 |
| ‘Before every treatment’ | 8 | 5.3 |
| ‘Other’ | 35 | 23.3 |
| Total | 150 | 100.0 |
|  | | |
| **F49 ‘Do you issue a drug application and dispersion form (‘AuA-Beleg’) for slaughter equines?’** | | |
| **Given answers** | **No. of answers** | **Answer percentage** |
| ‘Yes, always’ | 87 | 58.0 |
| ‘Yes, sometimes’ | 17 | 11.3 |
| ‘Yes, only if necessary’ | 26 | 17.3 |
| ‘Yes, rarely’ | 11 | 7.3 |
| ‘No, never’ | 9 | 6.0 |
| Total | 150 | 100.0 |
|  | | |
| **F50 ‘For which medications do you issue drug application and dispersion forms (‘AuA-Belege’)?’** | | |
| **Given answers** | **No. of answers** | **Answer percentage** |
| ‘For drugs with ‘waiting period’’ | 36 | 25.5 |
| ‘For ‘antimicrobial drugs’’ | 6 | 4.3 |
| ‘For ‘all administered drugs’’ | 90 | 63.8 |
| ‘For ‘all drugs that do not have to be documented in the equine passport’’ | 5 | 3.5 |
| ‘Other’ | 4 | 2.8 |
| Total | 141 | 100.0 |
|  | | |
| **F51 ‘When does the equine keeper receive the drug administration and dispersion form   (‘AuA-Beleg’)?’** | | |
| **Given answers** | **No. of answers** | **Answer percentage** |
| ‘Always immediately after application or dispensing’ | 41 | 29.1 |
| ‘Mostly immediately’ | 34 | 24.1 |
| ‘Usually later with the invoice’ | 45 | 31.9 |
| ‘The equine owner receive the AuA-Beleg’ | 15 | 10.6 |
| ‘Other’ | 6 | 4.3 |
| Total | 141 | 100.0 |
| **F52 ‘How well do you know the regulations of the ‘positive list’ (Reg. (EC) No. 1950/2006)?’** | | |
| **Answer options** | **No. of answers** | **Answer percentage** |
| ‘Very well’ | 13 | 8.7 |
| ‘Well’ | 57 | 38.3 |
| ‘Moderately’ | 56 | 37.6 |
| ‘Poorly’ | 14 | 9.4 |
| ‘I have no knowledge’ | 9 | 6.0 |
| Total | 149 | 100.0 |
|  | | |
| **F53 ‘Do you know which withdrawal period*** **must be observed when using a substance from the positive list?’** | | |
| **Answer options** | **No. of answers** | **Answer percentage** |
| ‘Yes’ | 101 | 67.8 |
| ‘No’ | 48 | 32.2 |
| Total | 149 | 100.0 |
| * Time that must pass between drug administration and slaughter of treated equine | | |

| **F54 ‘What is the withdrawal period* for substances of the positive list?’** | | | | | |
| --- | --- | --- | --- | --- | --- |
| **Given answers** | **No. of answers** | | **Answer percentage** | | **Classification of answer** |
| ‘21 days’ | 1 | | 1.0 | | Wrong |
| ‘28 days’ | 9 | | 8.9 | | Wrong |
| ‘48 days’ | 1 | | 1.0 | | Wrong |
| ‘53 days’ | 1 | | 1.0 | | Wrong |
| ‘6 months’ | 83 | | 82.2 | | Correct |
| No time period given or question not answered | 6 | | 5.9 | |  |
| Total | 101 | | 100.0 | |  |
| * Time that must pass between drug administration and slaughter of treated equine | | | | | |
|  | | | | | |
| **F60 ‘Do you know what withdrawal period* must be observed for the use of a reallocated^1^ drug?’** | | | | | |
| **Answer options** | | **No. of answers** | | **Answer percentage** | |
| **‘Yes’** | | 90 | | 67.2 | |
| **‘No’** | | 44 | | 32.8 | |
| **Total** | | 134 | | 100.0 | |
| * Time that must pass between drug administration and slaughter of treated equine | | | | | |
|  | | | | | |
| **F61 ‘What is the withdrawal period* to be observed for the use of a reallocated^1^ drug?’** | | | | | |
| **Given answers** | **No. of answers** | | **Answer percentage** | | **Classification of answer** |
| ‘28 days’ | 44 | | 48.9 | | Correct |
| ‘30 days’ | 1 | | 1.1 | | Wrong |
| ‘6 months’ | 39 | | 43.3 | | Wrong |
| No answer given | 6 | | 6.7 | | Wrong |
| Total | 90 | | 100.0 | |  |
| * Time that must pass between drug administration and slaughter of treated equine | | | | | |
|  | | | | | |
| **F62 ‘How do you perceive the overall documentation effort for equines destined for slaughter?’** | | | | | |
| **Answer options** | | **No. of answers** | | **Answer percentage** | |
| ‘Large’ | | 61 | | 45.9 | |
| ‘Rather large’ | | 49 | | 36.8 | |
| ‘Moderate’ | | 18 | | 13.5 | |
| ‘Rather small’ | | 4 | | 3.0 | |
| ‘Small’ | | 1 | | 0.8 | |
| Total | | 133 | | 100.0 | |
| **F63 ‘How do you perceive the complexity of the regulation regarding the ‘positive list’   (Reg. (EC) No. 1950/2006)?** | | | | | |
| **Answer options** | | **No. of answers** | | **Answer percentage** | |
| ‘Complicated’ | | 34 | | 25.6 | |
| ‘Rather complicated’ | | 57 | | 42.9 | |
| ‘Neither complicated nor simple’ | | 34 | | 25.6 | |
| ‘Rather simple’ | | 8 | | 6.0 | |
| ‘Simple’ | | 0 | | 0.0 | |
| Total | | 133 | | 100.0 | |
|  | | | | | |
| **F64 ‘Would you consider a uniformly structured equine passport independent from the breeding associations that issue them, as a simplification?’** | | | | | |
| **Answer options** | | **No. of answers** | | **Answer percentage** | |
| ‘Yes’ | | 106 | | 84.8 | |
| ‘No’ | | 19 | | 15.2 | |
| Total | | 125 | | 100.0 | |

| **F65 ‘Would you like more opportunities for advanced training regarding regulations of drug administration documentations, specifically for food-producing animals?’** | | |
| --- | --- | --- |
| **Answer options** | **No. of answers** | **Answer percentage** |
| ‘Yes’ | 65 | 60.7 |
| ‘No’ | 42 | 39.3 |
| Total | 107 | 100.0 |

**^1^**Usage that differs from the original drug registration, for example for a different indication.

**F** = Questions from the Questionnaires

The numeration and order of the tables follows the numeration and the order of the questions displayed in the questionnaires.

The gaps in the numeration result from the fact that data from questions that are not discussed in the study are not shown here.
