# Supplementary material for: A comparative survey of veterinarians, equine owners, and equine keepers regarding the knowledge and implementation of legal requirements in Germany for the use and documentation of veterinary medicines in equines intended for slaughter
Source: PLoS One. 2023 Apr 6;18(4):e0283371. doi: 10.1371/journal.pone.0283371 (PMC10079036; doi:10.1371/journal.pone.0283371)
Supplement: S5 Table — (DOCX) [file pone.0283371.s008.docx]

**Table S 5: Specific questions – Equine owners**

| F64 – All equine owners –  ‘From which sources do you obtain medication for your equine/s?’; Multiple answers could be given; N = 170 | | | | | |
| --- | --- | --- | --- | --- | --- |
| Answer options | | **No. of answers** | | **Answer percentage** | |
| ‘Veterinarian’ | | 161 | | 94.7 | |
| ‘Pharmacy’ | | 76 | | 44.7 | |
| ‘Websites’ | | 66 | | 38.8 | |
| ‘Animal healer’ | | 14 | | 8.2 | |
| ‘Farrier’ | | 8 | | 4.7 | |
| ‘Animal chiropractor’ | | 3 | | 1.8 | |
|  | | | | | |
| F64 – Self-classified slaughter equine owners –  ‘From which sources do you obtain medication for your equine/s?’; Multiple answers could be given; N = 32 | | | | | |
| Answer options | | **No. of answers** | | **Answer percentage** | |
| ‘Veterinarian’ | | 29 | | 90.6 | |
| ‘Pharmacy’ | | 17 | | 53.1 | |
| ‘Websites’ | | 12 | | 37.5 | |
| ‘Animal healer’ | | 4 | | 12.5 | |
| ‘Farrier’ | | 4 | | 12.5 | |
| ‘Animal chiropractor’ | | 1 | | 3.1 | |
|  | | | | | |
| F67 – All equine owners –  ‘Under which circumstances is an equine considered for slaughter?’ | | | | | |
| Answer options | **No. of answers** | | **Answer percentage** | | **Classification of answer** |
| ‘It is considered for slaughter after it is classified as such in the equine passport.’ | 59 | | 34.7 | | Wrong |
| ‘Every equine is considered for slaughter until its status is changed in the equine passport.’ | 100 | | 58.8 | | Correct |
| ‘There are no slaughter equines in Germany’ | 0 | | 0.0 | | Wrong |
| ‘Other’ | 2 | | 1.2 | | Wrong |
| ‘I do not know’ | 9 | | 5.3 | | Wrong |
| Total | 170 | | 100.0 | |  |
|  | | | | | |
| F67 – Self-classified slaughter equine owners –  ‘Under which circumstances is an equine considered for slaughter?’ | | | | | |
| Answer options | **No. of answers** | | **Answer percentage** | | **Classification of answer** |
| ‘It is considered for slaughter after it is classified as such in the equine passport.’ | 11 | | 34.4 | | Wrong |
| ‘Every equine is considered for slaughter until its status is changed in the equine passport.’ | 19 | | 59.4 | | Correct |
| ‘There are no slaughter equines in Germany’ | 0 | | 0.0 | | Wrong |
| ‘Other‘ | 0 | | 0.0 | | Wrong |
| ‘I do not know‘ | 2 | | 6.3 | | Wrong |
| Total | 32 | | 100.0 | |  |

| F77 – All equine owners –  ‘How frequently does your attending veterinarian inspect the equine passport/s?’ | | | | |
| --- | --- | --- | --- | --- |
| Given Answers | | **No. of answers** | | **Answer percentage** |
| ‘Before every treatment’ | | 16 | | 9.4 |
| ‘Depending on the type of treatment’ | | 81 | | 47.6 |
| ‘During first admission of the equine’ | | 12 | | 7.1 |
| ‘Never’ | | 10 | | 5.9 |
| ‘Other’, specified as ‘During vaccinations’ | | 47 | | 27.6 |
| ‘Other’ | | 1 | | 0.6 |
| ‘I do not know’ | | 3 | | 1.8 |
| Total | | 170 | | 100.0 |
|  | | | | |
| F77 – Self-classified slaughter equine owners –  ‘How frequently does your attending veterinarian inspect the equine passport/s?’ | | | | |
| Given answers | | **No. of answers** | | **Answer percentage** |
| ‘Before every treatment’ | | 4 | | 12.5 |
| ‘Depending on the type of treatment’ | | 14 | | 43.8 |
| ‘During first admission of the equine’ | | 1 | | 3.1 |
| ‘Never’ | | 2 | | 6.3 |
| ‘Other’, specified as ‘During vaccinations’ | | 10 | | 31.3 |
| ‘I do not know’ | | 1 | | 3.1 |
| Total | | 32 | | 100.0 |
|  | | | | |
| F78 – All equine owners –  ‘Do you know what drug application and dispersion forms (‘AuA-Belege’) are? | | | | |
| Answer options | | **No. of answers** | | **Answer percentage** |
| ‘Yes’ | | 69 | | 40.6 |
| ‘No’ | | 101 | | 59.4 |
| Total | | 170 | | 100.0 |
|  | | | | |
| F78 – Self-classified slaughter equine owners –  ‘Do you know what drug application and dispersion forms (‘AuA-Belege’) are?’ | | | | |
| Answer options | | **No. of answers** | | **Answer percentage** |
| ‘Yes’ | | 13 | | 40.6 |
| ‘No’ | | 19 | | 59.4 |
| Total | | 32 | | 100.0 |
|  | | | | |
| F79 – All equine owners –  ‘Do you receive drug application and dispersion forms (‘AuA-Belege’) from your attending veterinarians, for example in the scope of receiving anthelmintic treatment for your equine?’ | | | | |
| Answer options | **No. of answers** | | **Answer percentage** | |
| ‘Yes, always’ | 8 | | 11.6 | |
| ‘Yes, sometimes’ | 15 | | 21.7 | |
| ‘No’ | 46 | | 66.7 | |
| Total | 69 | | 100.0 | |
|  | | | | |
| F79 – Self-classified slaughter equine owners –  ‘Do you receive drug application and dispersion forms (‘AuA-Belege’) from your attending veterinarians, for example in the scope of receiving anthelmintic treatment for your equine?’ | | | | |
| Answer options | **No. of answers** | | **Answer percentage** | |
| ‘Yes, always’ | 1 | | 7.7 | |
| ‘Yes, sometimes’ | 4 | | 30.8 | |
| ‘No’ | 8 | | 61.5 | |
| Total | 13 | | 100.0 | |

| F80 – All equine owners –  ‘What do you do with the drug application and dispersion forms (‘AuA-Belege’) that you receive from your attending veterinarian?’ | | | |
| --- | --- | --- | --- |
| Answer options | **No. of answers** | **Answer percentage** | **Classification of answer** |
| ‘I save them for one year’ | 1 | 4.3 | Wrong |
| ‘I save them as long as I own the equine that received the document’ | 13 | 56.5 | Wrong |
| ‘I give it to the equine keeper that owns the stable where my equine is kept’ | 2 | 8.7 | Correct |
| ‘I throw them away’ | 4 | 17.4 | Wrong |
| ‘Other’ | 3 | 13.0 | Wrong |
| Total | 23 | 100.0 |  |

**F** = Questions from the Questionnaires

The numeration and order of the tables follows the numeration and the order of the questions displayed in the questionnaires.

The gaps in the numeration result from the fact that data from questions that are not discussed in the study are not shown here.
