# Supplementary material for: A comparative survey of veterinarians, equine owners, and equine keepers regarding the knowledge and implementation of legal requirements in Germany for the use and documentation of veterinary medicines in equines intended for slaughter
Source: PLoS One. 2023 Apr 6;18(4):e0283371. doi: 10.1371/journal.pone.0283371 (PMC10079036; doi:10.1371/journal.pone.0283371)
Supplement: S6 Table — (DOCX) [file pone.0283371.s009.docx]

**Table S 6: Specific questions – Equine keepers**

| **F18 ‘From which sources do you obtain medication for your equine/s?’; Multiple answers could be given; N = 70** | | |
| --- | --- | --- |
| **Answer options** | **No. of answers** | **Answer percentage** |
| ‘Veterinarian’ | 70 | 100.0 |
| ‘Pharmacy’ | 27 | 38.6 |
| ‘Websites’ | 28 | 40.0 |
| ‘Animal healer’ | 14 | 20.0 |
| ‘Farrier’ | 5 | 7.1 |
| ‘Animal chiropractor’ | 4 | 5.7 |
|  | | |
| **F21 ‘Is the administration of drugs documented either by yourself, the attending veterinarian, the equine owner, or by your staff?’** | | |
| **Answer options** | **No. of answers** | **Answer percentage** |
| ‘Yes, always’ | 34 | 48.6 |
| ‘Yes, sometimes’ | 15 | 21.4 |
| ‘No’ | 20 | 28.6 |
| ‘I do not know’ | 1 | 1.4 |
| Total | 70 | 100.0 |
|  | | |
| **F29 ‘How frequently do you inspect the equine passport before boarding a new equine in your stable?’** | | |
| **Answer options** | **No. of answers** | **Answer percentage** |
| ‘Always’ | 54 | 77.1 |
| ‘Most of the times’ | 6 | 8.6 |
| ‘Sometimes’ | 1 | 1.4 |
| ‘Seldom’ | 2 | 2.9 |
| ‘Never’ | 7 | 10.0 |
| Total | 70 | 100.0 |
|  | | |
| **F31 ‘How frequently does your attending veterinarian inspect the equine passport/s?’** | | |
| **Answer options** | **No. of answers** | **Answer percentage** |
| ‘Before every treatment’ | 9 | 12.9 |
| ‘Depending on the type of treatment’ | 47 | 67.1 |
| ‘During first admission of the equine’ | 5 | 7.1 |
| ‘Never’ | 7 | 10.0 |
| ‘No answer’ | 1 | 1.4 |
| ‘I do not know’ | 1 | 1.4 |
| Total | 70 | 100.0 |

| **F32 – Only-horse-keepers –**  **Under which circumstances is an equine considered for slaughter?** | | | | | |
| --- | --- | --- | --- | --- | --- |
| **Answer options** | **No. of answers** | | **Answer percentage** | | **Classification of answer** |
| ‘It is considered for slaughter after it is classified as such in the equine passport.’ | 17 | | 31.5 | | Wrong |
| ‘Every equine is considered for slaughter until its status is changed in the equine passport.’ | 33 | | 61.1 | | Correct |
| ‘I do not know‘ | 4 | | 7.4 | | Wrong |
| Total | 54 | | 100.0 | |  |
|  | | | | | |
| **F32 – Only-donkey and horse-and-donkey-keepers –**  **Under** **which circumstances is an equine considered for slaughter?** | | | | | |
| **Answer options** | **No. of answers** | | **Answer percentage** | | **Classification of answer** |
| ‘It is considered for slaughter after it is classified as such in the equine passport.’ | 9 | | 56.3 | | Wrong |
| ‘Every equine is considered for slaughter until its status is changed in the equine passport.’ | 7 | | 43.8 | | Correct |
| ‘I do not know’ | 0 | | 0.0 | | Wrong |
| Total | 16 | | 100.0 | |  |
|  | | | | | |
| **F33 ‘Is the status of companion animal documented in the equines´ passports for every equine in your stable that is not destined for slaughter?’** | | | | | |
| **Given answers** | | **No. of answers** | | **Answer percentage** | |
| ‘Yes, for every equine’ | | 43 | | 61.4 | |
| ‘Yes, for most of equines’ | | 16 | | 22.9 | |
| ‘No’ | | 6 | | 8.6 | |
| ‘I do not know’ | | 5 | | 7.1 | |
| Total | | 70 | | 100.0 | |
|  | | | | | |
| **F34 ‘How well do you know the documentation requirements of drug usage in equines   (THAMNV 2015)?’** | | | | | |
| **Answer options** | | **No. of answers** | | **Answer percentage** | |
| ‘Very well’ | | 10 | | 14.3 | |
| ‘Well’ | | 17 | | 24.3 | |
| ‘Moderate’ | | 19 | | 27.1 | |
| ‘Poor’ | | 14 | | 20.0 | |
| ‘Not at all’ | | 10 | | 14.3 | |
| Total | | 70 | | 100.0 | |
|  | | | | | |
| **F35 ‘Do you know what drug application and dispersion forms (‘AuA-Belege’) are?’** | | | | | |
| **Answer options** | | **No. of answers** | | **Answer percentage** | |
| ‘Yes’ | | 40 | | 57.1 | |
| ‘No’ | | 30 | | 42.9 | |
| Total | | 70 | | 100.0 | |

| **F36 ‘Do you receive drug application and dispersion forms (‘AuA-Belege’) from your attending veterinarians, for example in the scope of receiving anthelmintic treatment for equines you keep?’** | | | | | |
| --- | --- | --- | --- | --- | --- |
| **Answer options** | | **No. of answers** | | **Answer percentage** | |
| ‘Yes, always’ | | 14 | | 35.0 | |
| ‘Yes, sometimes’ | | 13 | | 32.5 | |
| ‘No’ | | 13 | | 32.5 | |
| Total | | 40 | | 100.0 | |
|  | | | | | |
| **F37 ‘Do you store the drug application and dispersion forms ‘(AuA-Belege’) which you receive from the veterinarian/s?’** | | | | | |
| **Answer options** | | **No. of answers** | | **Answer percentage** | |
| ‘Yes, always’ | | 21 | | 77.8 | |
| ‘Yes, sometimes’ | | 4 | | 14.8 | |
| ‘No’ | | 2 | | 7.4 | |
| Total | | 27 | | 100.0 | |
|  | | | | | |
| **F38 ‘How long do you store the drug application and dispersion forms (‘AuA-Belege’)?’** | | | | | |
| **Answer options** | **No. of answers** | | **Answer percentage** | | **Classification of answer** |
| ‘One year’ | 2 | | 8.0 | | Wrong |
| ‘Three years’ | 3 | | 12.0 | | Wrong |
| ‘Five years’ | 2 | | 8.0 | | Correct |
| ‘As long as I board the equine in my stable.’ | 16 | | 64.0 | | Wrong |
| ‘Until I can give them to the owner of the equines.’ | 2 | | 8.0 | | Wrong |
| Total | 25 | | 100.0 | |  |
|  | | | | | |
| **F40 ‘Is the administration of drugs documented by yourself?’** | | | | | |
| **Answer options** | | **No. of answers** | | **Answer percentage** | |
| ‘Yes, always’ | | 19 | | 27.1 | |
| ‘Yes, most of the times’ | | 8 | | 11.4 | |
| ‘Yes, seldomly’ | | 6 | | 8.6 | |
| ‘No’ | | 37 | | 52.9 | |
| Total | | 70 | | 100.0 | |
|  | | | | | |
| **F41 ‘Would you perceive a template on how to document drug administrations as helpful?’** | | | | | |
| **Answer options** | | **No. of answers** | | **Answer percentage** | |
| ‘Yes’ | | 30 | | 42.9 | |
| ‘No’ | | 20 | | 28.6 | |
| ‘Indifferent’ | | 20 | | 28.6 | |
| Total | | 70 | | 100.0 | |

**F** = Questions from the Questionnaires

The numeration and order of the tables follows the numeration and the order of the questions displayed in the questionnaires.

The gaps in the numeration result from the fact that data from questions that are not discussed in the study are not shown here.
