# Supplementary material for: A comparative survey of veterinarians, equine owners, and equine keepers regarding the knowledge and implementation of legal requirements in Germany for the use and documentation of veterinary medicines in equines intended for slaughter
Source: PLoS One. 2023 Apr 6;18(4):e0283371. doi: 10.1371/journal.pone.0283371 (PMC10079036; doi:10.1371/journal.pone.0283371)
Supplement: S2 File — (PDF) [file pone.0283371.s011.pdf]

S2 File – Questionnaire for equine owners

Tierarzneimitteldokumentation bei Equiden - Fragebogen für Pferdebesitzer*innen

Vielen Dank für Ihr Interesse und Ihre Unterstützung für das Forschungsvorhaben „Arzneimittelanwendung und Dokumentation bei Equiden".
Mein Name ist Shary Schneider, ich bin Tierärztin und schreibe eine Doktorarbeit an der Freien Universität Berlin am Fachbereich Veterinärmedizin.
Im Rahmen meiner Doktorarbeit befrage ich Tierbesitzer*innen, Stallbetreiber*innen und Tierärzte/Tierärztinnen zu verschiedenen Punkten der Arzneimittelanwendung bei Pferden und Eseln.
Ziel dieser Befragung ist es, den Stand der aktuell bei Equiden praktizierten Tiermedizin abzubilden. So sollen langfristig Ansatzpunkte zur Verbesserung der Arzneimittelsicherheit bei Pferden und Eseln gefunden werden.
Der nachfolgende Fragebogen für Pferdeigentümer*innen umfasst allgemeine Fragen, Behandlung und Anwendungen von Arzneimitteln, sowie Arzneimitteldokumentation.
Die Dauer der Befragung beträgt ca. 15 Minuten.
Sämtliche Daten werden nach den Richtlinien der DSGVO (Datenschutz-Grundverordnung) streng vertraulich behandelt. Eine personenbezogene Darstellung, sowie die Weitergabe an Dritte sind grundsätzlich ausgeschlossen. Dieses Forschungsprojekt steht in keinerlei Verbindung zur behördlichen Überwachung.
Hinweis: Für die Beantwortung mancher Fragen ist es hilfreich, wenn Sie den Equidenpass vorliegen haben.

Vielen Dank, dass Sie sich Zeit nehmen, um an dieser Studie teilzunehmen!
In dieser Umfrage sind 80 Fragen enthalten.

Veterinary drug documentation for equines - Questionnaire for equine owners

Thank you for your interest and support for the research project "Medication use and documentation in equines".
My name is Shary Schneider, I am a veterinarian and I am writing a doctoral thesis at the Freie Universität Berlin in the Department of Veterinary Medicine.
As part of my doctoral thesis, I am interviewing animal owners, stable owners, and veterinarians about various aspects of drug use in horses and donkeys.
The aim of this survey is to map the state of veterinary medicine currently practiced for equids. In this way, starting points for improving drug safety in horses and donkeys will be found in the long term.
The following questionnaire for equine owners covers general questions, treatment and use of medicines, as well as medicine documentation.
The duration of the survey is approximately 15 minutes.
All data will be treated as strictly confidential according to the guidelines of the DSGVO (Data Protection Regulation). Any personal representation or passing any data on to third parties are principally excluded. This research project is in no way connected to official monitoring.
Note: For answering some questions it is helpful if you have the equine passport available.

Thank you for taking the time to participate in this study!
There are 80 questions in this survey.

Demographische Fragen / Demographic questions

F 1

In welchem Bundesland steht Ihr Pferd / Ihre Pferde? *

Bitte wählen Sie nur eine der folgenden Antworten aus:

- o Baden-Württemberg
- o Bayern
- o Berlin
- o Brandenburg
- o Bremen
- o Hamburg
- o Hessen
- o Mecklenburg-Vorpommern
- o Niedersachsen
- o Nordrhein-Westfalen
- o Rheinland-Pfalz
- o Saarland
- o Sachsen
- o Sachsen-Anhalt
- o Schleswig-Holstein
- o Thüringen

In which federal state is / are your equine(s) kept? *

Please select only one of the following answers:

- o Baden-Wuerttemberg
- o Bavaria
- o Berlin
- o Brandenburg
- o Bremen
- o Hamburg
- o Hessia
- o Mecklenburg-Western Pomerania
- o Lower Saxony
- o North Rhine-Westphalia
- o Rhineland-Palatinate
- o Saarland
- o Saxony
- o Saxony-Anhalt
- o Schleswig-Holstein
- o Thuringia

F 2

Wie viele Pferde besitzen Sie? *

Bitte wählen Sie eine der folgenden Antworten:

- o 1 Pferd
- o 2 Pferde
- o 3 Pferde
- o 4 Pferde
- o 5 oder mehr Pferde

How many equines do you own? *

Please select one of the following answers:

- o 1 equine
- o 2 equines
- o 3 equines
- o 4 equines
- o 5 or more equines

F 3

In welchem Jahr wurde Ihr Pferd geboren? *

Diese Frage wird nur angezeigt, wenn folgende Bedingungen erfüllt sind:
Antwort war '1 Pferd' bei Frage '2'.
In dieses Feld dürfen nur Zahlen eingegeben werden.
Bitte geben Sie Ihre Antwort hier ein:

Bitte nur die Jahreszahl eingeben.

In what year was your equine born? *

This question is only displayed if the following conditions are met:
Answer was '1 equine' for question 2.
Only numbers may be entered in this field.
Please enter your answer here:

Please enter only the year.

F 4

In welchem Jahr wurden Ihre Pferde geboren? *
Diese Frage wird nur angezeigt, wenn folgende Bedingungen erfüllt sind:
Antwort war '2 Pferde' bei Frage '2'.
Nur Zahlen dürfen in diese Felder eingegeben werden.

Bitte geben Sie Ihre Antwort(en) hier ein:
Pferd 1 [ ]

Pferd 2 [ ]

Bitte tragen Sie nur die Jahreszahl ein.

In what year were your equines born? *
This question is only displayed if the following conditions are met:
Answer was '2 equines' for question 2.
Only numbers may be entered in these fields.

Please enter your answer(s) here:

Equine 1: [ ]

Equine 2: [ ]

Please enter only the year.

F 5

In welchem Jahr wurden Ihre Pferde geboren? *
Diese Frage wird nur angezeigt, wenn folgende Bedingungen erfüllt sind:
Antwort war '3 Pferde' bei Frage '2'.
Nur Zahlen dürfen in diese Felder eingegeben werden.
Bitte geben Sie Ihre Antwort(en) hier ein:

Pferd 1 [ ]

Pferd 2 [ ]

Pferd 3 [ ]

Bitte tragen Sie nur die Jahreszahl ein.

In what year were your equines born? *
This question is only displayed if the following conditions are met:
Answer was '3 equines' for question 2.
Only numbers may be entered in these fields.
Please enter your answer(s) here:

Equine 1: [ ]

Equine 2: [ ]

Equine 3: [ ]

Please enter only the year.

F 6

In welchem Jahr wurden Ihre Pferde geboren? *
Diese Frage wird nur angezeigt, wenn folgende Bedingungen erfüllt sind:
Antwort war '4 Pferde' bei Frage '2'.
Nur Zahlen dürfen in diese Felder eingegeben werden.
Bitte geben Sie Ihre Antwort(en) hier ein:

Pferd 1 [ ]

Pferd 2 [ ]

Pferd 3 [ ]

Pferd 4 [ ]

Bitte tragen Sie nur die Jahreszahl ein.

In what year were your equines born? *
This question is only displayed if the following conditions are met:
Answer was '4 equines' for question 2.
Only numbers may be entered in these fields.
Please enter your answer(s) here:
Equine 1: [ ]

Equine 2: [ ]

Equine 3: [ ]

Equine 4: [ ]

Please enter the year only.

F 7

In welchem Jahr wurden Ihre Pferde geboren? *
Diese Frage wird nur angezeigt, wenn folgende Bedingungen erfüllt sind:
Antwort war '5 oder mehr Pferde' bei Frage '2'.
Nur Zahlen dürfen in diese Felder eingegeben werden.
Bitte geben Sie Ihre Antwort(en) hier ein:

Pferd 1 [ ]

Pferd 2 [ ]

Pferd 3 [ ]

Pferd 4 [ ]

Pferd 5 [ ]

Bitte tragen Sie nur die Jahreszahl ein.

Die folgenden Fragen sind für maximal fünf Pferde ausgelegt.
Falls Sie mehr als fünf Pferde besitzen, legen Sie sich bitte fest, für welche Ihrer Pferde Sie den Fragebogen ausfüllen.
Sie können den Fragebogen für weitere Pferde erneut ausfüllen.

In what year were your equines born? *
This question is only displayed if the following conditions are met:
Answer was '5 or more equines' for question 2.
Only numbers may be entered in these fields.
Please enter your answer(s) here:

Equine 1: [ ]

Equine 2: [ ]

Equine 3: [ ]

Equine 4: [ ]

Equine 5: [ ]

Please fill in the year only.

The following questions are designed for a maximum of five equines.
If you own more than five equines, please determine which of your equines you are completing the questionnaire for.

F 8

Welcher Rasse gehört Ihr Pferd an? *
Diese Frage wird nur angezeigt, wenn folgende Bedingungen erfüllt sind:
Antwort war '1 Pferd' bei Frage '2'.
Bitte geben Sie Ihre Antwort hier ein:
[ ]

What breed is your equine? *
This question is only displayed if the following conditions are met:
Answer was '1 equine' for question 2.
Please enter your answer here:
[ ]

F 9

Welcher Rasse gehören Ihre Pferde an? *
Diese Frage wird nur angezeigt, wenn folgende Bedingungen erfüllt sind:
Antwort war '2 Pferde' bei Frage '2'.

Pferd 1: [ ]

Pferd 2: [ ]

What breed are your equines? *
This question is only displayed if the following conditions are met:
Answer was '2 equines' for question 2.

Equine 1: [ ]

Equine 2: [ ]

F 10

Deutsch	English
**Welcher Rasse gehören Ihre Pferde an? ***	**What breed are your equines? ***
Diese Frage wird nur angezeigt, wenn folgende Bedingungen erfüllt sind:	This question is only displayed if the following conditions are met:
Antwort war '3 Pferde' bei Frage '2'.	Answer was '3 equines' for question 2.
Pferd 1:	Equine 1:
Pferd 2:	Equine 2:
Pferd 3:	Equine 3:

F 11

Deutsch	English
**Welcher Rasse gehören Ihre Pferde an? ***	**What breed are your equines? ***
Diese Frage wird nur angezeigt, wenn folgende Bedingungen erfüllt sind:	This question is only displayed if the following conditions are met:
Antwort war '4 Pferde' bei Frage '2'.	Answer was '4 equines' for question 2.
Pferd 1:	Equine 1:
Pferd 2:	Equine 2:
Pferd 3:	Equine 3:
Pferd 4:	Equine 4:

F 12

Deutsch	English
**Welcher Rasse gehören Ihre Pferde an? ***	**What breed are your equines? ***
Diese Frage wird nur angezeigt, wenn folgende Bedingungen erfüllt sind:	This question is only displayed if the following conditions are met:
Antwort war '5 oder mehr Pferde' bei Frage '2'.	Answer was '5 or more equines' for question 2.
Pferd 1:	Equine 1:
Pferd 2:	Equine 2:
Pferd 3:	Equine 3:
Pferd 4:	Equine 4:
Pferd 5:	Equine 5:

F 13

Wie wird Ihr Pferd überwiegend genutzt? *

Diese Frage wird nur angezeigt, wenn folgende Bedingungen erfüllt sind:
Antwort war '1 Pferd' bei Frage '2'.
Bitte wählen Sie alle zutreffenden Antworten aus:

- o Freizeitpferd
- o Springreiten
- o Dressurreiten
- o Westernreiten
- o Vielseitigkeitsreiten
- o Distanzreiten
- o Zucht
- o Sonstiges: [ ]

Falls Sie "Sonstiges" wählen, können Sie dies im Kommentarfeld erläutern.

How is your equine predominantly used? *

This question is only displayed if the following conditions are met:
Answer was '1 equine' for question 2.
Please select all that apply:

- o Recreational horse
- o Show jumping
- o Dressage
- o Western riding
- o Eventing
- o Endurance riding
- o Breeding
- o Other: [ ]

If you select "Other", you can explain in the comment field.

F 14

Wie werden Ihre Pferde überwiegend genutzt? *

Diese Frage wird nur angezeigt, wenn folgende Bedingungen erfüllt sind:
Antwort war '2 Pferde' bei Frage '2'.
Bitte wählen Sie die zutreffende Antwort für jeden Punkt aus:

Pferd 1:

- o Freizeitpferd
- o Springreiten
- o Dressurreiten
- o Westernreiten
- o Vielseitigkeitsreiten
- o Distanzreiten
- o Zucht
- o Sonstiges: [ ]

Pferd 2:

- o Freizeitpferd
- o Springreiten
- o Dressurreiten
- o Westernreiten
- o Vielseitigkeitsreiten
- o Distanzreiten
- o Zucht
- o Sonstiges: [ ]

How are your equines predominantly used? *

This question is only displayed if the following conditions are met:
Answer was '2 equines' for question 2.
Please select the applicable answer for each item:

Equine 1:

- o Recreational horse
- o Show jumping
- o Dressage
- o Western riding
- o Eventing
- o Endurance riding
- o Breeding
- o Other: [ ]

Equine 2:

- o Recreational horse
- o Show jumping
- o Dressage
- o Western riding
- o Eventing
- o Endurance riding
- o Breeding
- o Other: [ ]

F 15

Wie werden Ihre Pferde überwiegend genutzt? *

Diese Frage wird nur angezeigt, wenn folgende Bedingungen erfüllt sind:
Antwort war '3 Pferde' bei Frage '2'.
Bitte wählen Sie die zutreffende Antwort für jeden Punkt aus:

Pferd 1:
- Freizeitpferd
- Springreiten
- Dressurreiten
- Westernreiten
- Vielseitigkeitsreiten
- Distanzreiten
- Zucht
- Sonstiges: [ ]

Pferd 2:
- Freizeitpferd
- Springreiten
- Dressurreiten
- Westernreiten
- Vielseitigkeitsreiten
- Distanzreiten
- Zucht
- Sonstiges: [ ]

Pferd 3:
- Freizeitpferd
- Springreiten
- Dressurreiten
- Westernreiten
- Vielseitigkeitsreiten
- Distanzreiten
- Zucht
- Sonstiges: [ ]

How are your equines predominantly used? *

This question is only displayed if the following conditions are met:
Answer was '3 equines' for question 2.
Please select the applicable answer for each item:

Equine 1:
- Recreational horse
- Show jumping
- Dressage
- Western riding
- Eventing
- Endurance riding
- Breeding
- Other: [ ]

Equine 2:
- Recreational horse
- Show jumping
- Dressage
- Western riding
- Eventing
- Endurance riding
- Breeding
- Other: [ ]

Equine 3:
- Recreational horse
- Show jumping
- Dressage
- Western riding
- Eventing
- Endurance riding
- Breeding
- Other: [ ]

F 16

Wie werden Ihre Pferde überwiegend genutzt? *

Diese Frage wird nur angezeigt, wenn folgende Bedingungen erfüllt sind:
Antwort war '4 Pferde' bei Frage '2'.
Bitte wählen Sie die zutreffende Antwort für jeden Punkt aus:

Pferd 1:
- Freizeitpferd
- Springreiten
- Dressurreiten
- Westernreiten
- Vielseitigkeitsreiten
- Distanzreiten
- Zucht
- Sonstiges: [ ]

Pferd 2:
- Freizeitpferd
- Springreiten
- Dressurreiten
- Westernreiten
- Vielseitigkeitsreiten
- Distanzreiten
- Zucht
- Sonstiges: [ ]

Pferd 3:
- Freizeitpferd
- Springreiten
- Dressurreiten
- Westernreiten
- Vielseitigkeitsreiten
- Distanzreiten
- Zucht
- Sonstiges: [ ]

Pferd 4:
- Freizeitpferd
- Springreiten
- Dressurreiten
- Westernreiten
- Vielseitigkeitsreiten
- Distanzreiten
- Zucht
- Sonstiges: [ ]

How are your equines predominantly used? *

This question is only displayed if the following conditions are met:
Answer was '4 equines' for question 2.
Please select the applicable answer for each item:

Equine 1:
- Recreational horse
- Show jumping
- Dressage
- Western riding
- Eventing
- Endurance riding
- Breeding
- Other: [ ]

Equine 2:
- Recreational horse
- Show jumping
- Dressage
- Western riding
- Eventing
- Endurance riding
- Breeding
- Other: [ ]

Equine 3:
- Recreational horse
- Show jumping
- Dressage
- Western riding
- Eventing
- Endurance riding
- Breeding
- Other: [ ]

Equine 4:
- Recreational horse
- Show jumping
- Dressage
- Western riding
- Eventing
- Endurance riding
- Breeding
- Other: [ ]

F 17

Wie werden Ihre Pferde überwiegend genutzt? *
Diese Frage wird nur angezeigt, wenn folgende Bedingungen erfüllt sind:
Antwort war '5 oder mehr Pferde' bei Frage '2'.
Bitte wählen Sie die zutreffende Antwort für jeden Punkt aus:

Pferd 1:
- Freizeitpferd
- Springreiten
- Dressurreiten
- Westernreiten
- Vielseitigkeitsreiten
- Distanzreiten
- Zucht
- Sonstiges: [ ]

Pferd 2:
- Freizeitpferd
- Springreiten
- Dressurreiten
- Westernreiten
- Vielseitigkeitsreiten
- Distanzreiten
- Zucht
- Sonstiges: [ ]

Pferd 3:
- Freizeitpferd
- Springreiten
- Dressurreiten
- Westernreiten
- Vielseitigkeitsreiten
- Distanzreiten
- Zucht
- Sonstiges: [ ]

Pferd 4:
- Freizeitpferd
- Springreiten
- Dressurreiten
- Westernreiten
- Vielseitigkeitsreiten
- Distanzreiten
- Zucht
- Sonstiges: [ ]

Pferd 5:
- Freizeitpferd
- Springreiten
- Dressurreiten
- Westernreiten
- Vielseitigkeitsreiten
- Distanzreiten
- Zucht
- Sonstiges: [ ]

How are your equines predominantly used? *
This question is only displayed if the following conditions are met:
Answer was '5 or more equines' for question 2.
Please select the applicable answer for each item:

Equine 1:
- Recreational horse
- Show jumping
- Dressage
- Western riding
- Eventing
- Endurance riding
- Breeding
- Other: [ ]

Equine 2:
- Recreational horse
- Show jumping
- Dressage
- Western riding
- Eventing
- Endurance riding
- Breeding
- Other: [ ]

Equine 3:
- Recreational horse
- Show jumping
- Dressage
- Western riding
- Eventing
- Endurance riding
- Breeding
- Other: [ ]

Equine 4:
- Recreational horse
- Show jumping
- Dressage
- Western riding
- Eventing
- Endurance riding
- Breeding
- Other: [ ]

Equine 5:
- Recreational horse
- Show jumping
- Dressage
- Western riding
- Eventing
- Endurance riding
- Breeding
- Other: [ ]

F 18

Nehmen Sie mit Ihrem Pferd an Turnieren teil? *
Diese Frage wird nur angezeigt, wenn folgende Bedingungen erfüllt sind:
Antwort war '1 Pferd' bei Frage '2'.
Bitte wählen Sie nur eine der folgenden Antworten aus:
- o Ja
- o Nein

Do you participate in competitions with your equine? *
This question is only displayed if the following conditions are met:
Answer was '1 equine' for question 2.
Please select only one of the following answers:
- o Yes
- o No

F 19

Nehmen Sie mit Ihren Pferden an Turnieren teil? *
Diese Frage wird nur angezeigt, wenn folgende Bedingungen erfüllt sind:
Antwort war '2 Pferde' bei Frage '2'.
Bitte wählen Sie die zutreffende Antwort für jeden Punkt aus:
Pferd 1:
- o Ja
- o Nein

Pferd 2:
- o Ja
- o Nein

Do you participate in competitions with your equines? *
This question is only displayed if the following conditions are met:
Answer was '2 equines' for question 2.
Please select the applicable answer for each item:
Equine 1:
- o Yes
- o No

Equine 2:
- o Yes
- o No

F 20

Nehmen Sie mit Ihren Pferden an Turnieren teil? *
Diese Frage wird nur angezeigt, wenn folgende Bedingungen erfüllt sind:
Antwort war '3 Pferde' bei Frage '2'.
Bitte wählen Sie die zutreffende Antwort für jeden Punkt aus:
Pferd 1:
- o Ja
- o Nein

Pferd 2:
- o Ja
- o Nein

Pferd 3:
- o Ja
- o Nein

Do you participate in competitions with your equines? *
This question is only displayed if the following conditions are met:
Answer was '3 equines' for question 2.
Please select the applicable answer for each item:
Equine 1:
- o Yes
- o No

Equine 2:
- o Yes
- o No

Equine 3:
- o Yes
- o No

F 21

Nehmen Sie mit Ihren Pferden an Turnieren teil? *
Diese Frage wird nur angezeigt, wenn folgende Bedingungen erfüllt sind:
Antwort war '4 Pferde' bei Frage '2'.
Bitte wählen Sie die zutreffende Antwort für jeden Punkt aus:
Pferd 1:
- Ja
- Nein

Pferd 2:
- Ja
- Nein

Pferd 3:
- Ja
- Nein

Pferd 4:
- Ja
- Nein

Do you participate in competitions with your equines? *
This question is only displayed if the following conditions are met:
Answer was '4 equines' for question 2.
Please select the applicable answer for each item:
Equine 1:
- Yes
- No

Equine 2:
- Yes
- No

Equine 3:
- Yes
- No

Equine 4:
- Yes
- No

F 22

Nehmen Sie mit Ihren Pferden an Turnieren teil? *
Diese Frage wird nur angezeigt, wenn folgende Bedingungen erfüllt sind:
Antwort war '5 oder mehr Pferde' bei Frage '2'.
Bitte wählen Sie die zutreffende Antwort für jeden Punkt aus:
Pferd 1:
- Ja
- Nein

Pferd 2:
- Ja
- Nein

Pferd 3:
- Ja
- Nein

Pferd 4:
- Ja
- Nein

Pferd 5:
- Ja
- Nein

Do you participate in competitions with your equines? *
This question is only displayed if the following conditions are met:
Answer was '5 or more equines' for question 2.
Please select the applicable answer for each item:
Equine 1:
- Yes
- No

Equine 2:
- Yes
- No

Equine 3:
- Yes
- No

Equine 4:
- Yes
- No

Equine 5:
- Yes
- No

F 23

In welchem Land wurde Ihr Pferd geboren? *

Diese Frage wird nur angezeigt, wenn folgende Bedingungen erfüllt sind:

Antwort war '1 Pferd' bei Frage '2'.

Bitte geben Sie Ihre Antwort hier ein:

In which country was your equine born? *

This question is only displayed if the following conditions are met:

Answer was '1 equine' for question 2.

Please enter your answer here:

F 24

In welchem Land wurden Ihre Pferde geboren? *

Diese Frage wird nur angezeigt, wenn folgende Bedingungen erfüllt sind:

Antwort war '2 Pferde' bei Frage '2'.

Pferd 1:

Pferd 2:

In which country were your equines born? *

This question is only displayed if the following conditions are met:

Answer was '2 equines' for question 2.

Equine 1:

Equine 2:

F 25

In welchem Land wurden Ihre Pferde geboren? *

Diese Frage wird nur angezeigt, wenn folgende Bedingungen erfüllt sind:

Antwort war '3 Pferde' bei Frage '2'.

Pferd 1:

Pferd 2:

Pferd 3:

In which country were your equines born? *

This question is only displayed if the following conditions are met:

Answer was '3 equines' for question 2.

Equine 1:

Equine 2:

Equine 3:

F 26

In welchem Land wurden Ihre Pferde geboren? *

Diese Frage wird nur angezeigt, wenn folgende Bedingungen erfüllt sind:

Antwort war '4 Pferde' bei Frage '2'.

Pferd 1:

Pferd 2:

Pferd 3:

Pferd 4:

In which country were your equines born? *

This question is only displayed if the following conditions are met:

Answer was '4 equines' for question 2.

Equine 1:

Equine 2:

Equine 3:

Equine 4:

F 27

In welchem Land wurden Ihre Pferde geboren? *
Diese Frage wird nur angezeigt, wenn folgende Bedingungen erfüllt sind:
Antwort war '5 oder mehr Pferde' bei Frage '2'.

Pferd 1: ________

Pferd 2: ________

Pferd 3: ________

Pferd 4: ________

Pferd 5: ________

In which country were your equines born? *
This question is only displayed if the following conditions are met:
Answer was '5 or more equines' for question 2.

Equine 1: ________

Equine 2: ________

Equine 3: ________

Equine 4: ________

Equine 5: ________

Kastration / Specialized questions – castration

F 28

Besitzen Sie einen Wallach? *
Diese Frage wird nur angezeigt, wenn folgende Bedingungen erfüllt sind:
Antwort war '1 Pferd' bei Frage '2'.
Bitte wählen Sie nur eine der folgenden Antworten aus:
- Ja
- Nein

Do you own a gelding? *
This question is only displayed if the following conditions are met:
Answer was '1 equine' for question 2.
Please select only one of the following answers:
- Yes
- No

F 29

Besitzen Sie einen oder mehrere Wallache? *
Diese Frage wird nur angezeigt, wenn folgende Bedingungen erfüllt sind:
Antwort war '2 Pferde' bei Frage '2'.
Bitte wählen Sie nur eine der folgenden Antworten aus:
- Ja, einen Wallach
- Ja, zwei Wallache
- Nein, ich besitze keinen Wallach

Do you own one or more geldings? *
This question is only displayed if the following conditions are met:
Answer was '2 equines' for question 2.
Please select only one of the following answers:
- Yes, one gelding
- Yes, two geldings
- No, I do not own a gelding

F 30

Besitzen Sie einen oder mehrere Wallache? *
Diese Frage wird nur angezeigt, wenn folgende Bedingungen erfüllt sind:
Antwort war '3 Pferde' bei Frage '2'.
Bitte wählen Sie nur eine der folgenden Antworten aus:
- Ja, ich besitze einen Wallach
- Ja, ich besitze zwei Wallache
- Ja, ich besitze drei Wallache
- Nein, ich besitze keine Wallache

Do you own one or more geldings? *
This question is only displayed if the following conditions are met:
Answer was '3 equines' for question 2.
Please select only one of the following answers:
- Yes, I own one gelding
- Yes, I own two geldings
- Yes, I own three geldings
- No, I do not own any geldings

F 31

Besitzen Sie einen oder mehrere Wallache? *
Diese Frage wird nur angezeigt, wenn folgende Bedingungen erfüllt sind:
Antwort war '4 Pferde' bei Frage '2'.
Bitte wählen Sie nur eine der folgenden Antworten aus:
- Ja, ich besitze einen Wallach
- Ja, ich besitze zwei Wallache
- Ja, ich besitze drei Wallache
- Ja, ich besitze vier Wallache
- Nein, ich besitze keine Wallache

Do you own one or more geldings? *
This question is only displayed if the following conditions are met:
Answer was '4 equines' for question 2.
Please select only one of the following answers:
- Yes, I own one gelding
- Yes, I own two geldings
- Yes, I own three geldings
- Yes, I own four geldings
- No, I do not own any geldings

F 32

Besitzen Sie einen oder mehrere Wallache? *
Diese Frage wird nur angezeigt, wenn folgende Bedingungen erfüllt sind:
Antwort war '5 oder mehr Pferde' bei Frage '2'.
Bitte wählen Sie nur eine der folgenden Antworten aus:

- o Ja, ich besitze einen Wallach
- o Ja, ich besitze zwei Wallache
- o Ja, ich besitze drei Wallache
- o Ja, ich besitze vier Wallache
- o Ja, ich besitze fünf Wallache
- o Nein, ich besitze keine Wallache

Do you own one or more geldings? *
This question is only displayed if the following conditions are met:
Answer was '5 or more equines' for question 2.
Please select only one of the following answers:

- o Yes, I own one gelding
- o Yes, I own two geldings
- o Yes, I own three geldings
- o Yes, I own four geldings
- o Yes, I own five geldings
- o No, I do not own any geldings

F 33

Ist die Kastration im Pferdepass vermerkt? *
Diese Frage wird nur angezeigt, wenn folgende Bedingungen erfüllt sind:
-------- Scenario 16 --------
Antwort war 'Ja' bei Frage '28'
-------- oder Scenario 17 --------
Antwort war 'Ja, einen Wallach' bei Frage '29'
-------- oder Scenario 18 --------
Antwort war 'Ja, ich besitze einen Wallach' bei Frage '30'
-------- oder Scenario 19 --------
Antwort war 'Ja, ich besitze einen Wallach' bei Frage '31'
-------- oder Scenario 20 --------
Antwort war 'Ja, ich besitze einen Wallach' bei Frage '32'
Bitte wählen Sie nur eine der folgenden Antworten aus:

- o Ja
- o Nein
- o Ich weiß es nicht

Is the castration noted in the equine's passport? *
This question is only displayed if the following conditions are met:
-------- Scenario 16 --------
Answer was 'Yes' to question 28
-------- or Scenario 17 --------
Answer was 'Yes, I own one gelding' at question 29.
-------- or Scenario 18 --------
Answer was 'Yes, I own one gelding' at question 30.
-------- or Scenario 19 --------
Answer was 'Yes, I own one gelding' at question 31.
-------- or Scenario 20 --------
Answer was 'Yes, I own one gelding' at question 32.
Please select only one of the following answers:

- o Yes
- o No
- o I do not know

F 34

Sind die Kastrationen in den Pferdepässen vermerkt? *
Diese Frage wird nur angezeigt, wenn folgende Bedingungen erfüllt sind:
-------- Scenario 21 --------
Antwort war 'Ja, zwei Wallache' bei Frage '29'
-------- oder Scenario 22 --------
Antwort war 'Ja, ich besitze zwei Wallache' bei Frage '30'
-------- oder Scenario 23 --------
Antwort war 'Ja, ich besitze zwei Wallache' bei Frage '31'
-------- oder Scenario 24 --------
Antwort war 'Ja, ich besitze zwei Wallache' bei Frage '32'
Bitte wählen Sie die zutreffende Antwort für jeden Punkt aus:
Wallach 1:
- o Ja
- o Nein
- o Ich weiß es nicht

Wallach 2:
- o Ja
- o Nein
- o Ich weiß es nicht

Are the castrations noted in the equine's passports? *
This question is only displayed if the following conditions are met:
-------- Scenario 21 --------
Answer was 'Yes, two geldings' at question 29
-------- or Scenario 22 --------
Answer was 'Yes, I own two geldings' at question 30.
-------- or Scenario 23 --------
Answer was 'Yes, I own two geldings' at question 31.
-------- or Scenario 24 --------
Answer was 'Yes, I own two geldings' at question 32.
Please select the applicable answer for each item:
Gelding 1:
- o Yes
- o No
- o I do not know

Gelding 2:
- o Yes
- o No
- o I do not know

F 35

Sind die Kastrationen in den Pferdepässen vermerkt? *
Diese Frage wird nur angezeigt, wenn folgende Bedingungen erfüllt sind:
-------- Scenario 25 --------
Antwort war 'Ja, ich besitze drei Wallache' bei Frage '30'
-------- oder Scenario 26 --------
Antwort war 'Ja, ich besitze drei Wallache' bei Frage '31'
-------- oder Scenario 27 --------
Antwort war 'Ja, ich besitze drei Wallache' bei Frage '32'
Bitte wählen Sie die zutreffende Antwort für jeden Punkt aus:
Wallach 1:
- o Ja
- o Nein
- o Ich weiß es nicht

Wallach 2:
- o Ja
- o Nein
- o Ich weiß es nicht

Wallach 3:
- o Ja
- o Nein
- o Ich weiß es nicht

Are the castrations noted in the equine's passports? *
This question is only displayed if the following conditions are met:
-------- Scenario 25 --------
Answer was 'Yes, I own three geldings' at question 30
-------- or Scenario 26 --------
Answer was 'Yes, I own three geldings' at question 31.
-------- or Scenario 27 --------
Answer was 'Yes, I own three geldings' at question 32.
Please select the applicable answer for each item:
Gelding 1:
- o Yes
- o No
- o I do not know

Gelding 2:
- o Yes
- o No
- o I do not know

Gelding 3:
- o Yes
- o No
- o I do not know

F 36

Sind die Kastrationen in den Pferdepässen vermerkt? *

Diese Frage wird nur angezeigt, wenn folgende Bedingungen erfüllt sind:

-------- Scenario 28 --------

Antwort war 'Ja, ich besitze vier Wallache' bei Frage '3'

-------- oder Scenario 29 --------

Antwort war 'Ja, ich besitze vier Wallache' bei Frage '32'

Bitte wählen Sie die zutreffende Antwort für jeden Punkt aus:

Wallach 1:
- Ja
- Nein
- Ich weiß es nicht

Wallach 2:
- Ja
- Nein
- Ich weiß es nicht

Wallach 3:
- Ja
- Nein
- Ich weiß es nicht

Wallach 4:
- Ja
- Nein
- Ich weiß es nicht

Are the castrations noted in the equine's passports? *

This question is only displayed if the following conditions are met:

-------- Scenario 28 --------

Answer was 'Yes, I own four geldings' at question 31

-------- or Scenario 29 --------

Answer was 'Yes, I own four geldings' at question 32.

Please select the applicable answer for each item:

Gelding 1:
- Yes
- No
- I do not know

Gelding 2:
- Yes
- No
- I do not know

Gelding 3:
- Yes
- No
- I do not know

Gelding 4:
- Yes
- No
- I do not know

F 37

Sind die Kastrationen in den Pferdepässen vermerkt? *
Diese Frage wird nur angezeigt, wenn folgende Bedingungen erfüllt sind:
Antwort war 'Ja, ich besitze fünf Wallache' bei Frage '32'
Bitte wählen Sie die zutreffende Antwort für jeden Punkt aus:
Wallach 1:
- Ja
- Nein
- Ich weiß es nicht

Wallach 2:
- Ja
- Nein
- Ich weiß es nicht

Wallach 3:
- Ja
- Nein
- Ich weiß es nicht

Wallach 4:
- Ja
- Nein
- Ich weiß es nicht

Wallach 5:
- Ja
- Nein
- Ich weiß es nicht

Are the castrations noted in the equine's passports? *
This question is only displayed if the following conditions are met:
Answer was 'Yes, I own five geldings' for question 32.
Please select the applicable answer for each item:
Gelding 1:
- Yes
- No
- I do not know

Gelding 2:
- Yes
- No
- I do not know

Gelding 3:
- Yes
- No
- I do not know

Gelding 4:
- Yes
- No
- I do not know

Gelding 5:
- Yes
- No
- I do not know

F 38

In welchem Jahr wurde Ihr Pferd kastriert?
Diese Frage wird nur angezeigt, wenn folgende Bedingungen erfüllt sind:
-------- Scenario 16 --------
Antwort war 'Ja' bei Frage '28'
-------- oder Scenario 17 --------
Antwort war 'Ja, einen Wallach' bei Frage '29'
-------- oder Scenario 18 --------
Antwort war 'Ja, ich besitze einen Wallach' bei Frage '30'
-------- oder Scenario 19 --------
Antwort war 'Ja, ich besitze einen Wallach' bei Frage '3'
-------- oder Scenario 20 --------
Antwort war 'Ja, ich besitze einen Wallach' bei Frage '32'
In dieses Feld dürfen nur Zahlen eingegeben werden.
Bitte geben Sie Ihre Antwort hier ein:

Falls Sie nicht wissen, in welchem Jahr Ihr Pferd kastriert wurde, dann überspringen Sie die Frage.

In which year was your equine castrated?
This question is only displayed if the following conditions are met:
-------- Scenario 16 --------
Answer was 'Yes' to question 28
-------- or Scenario 17 --------
Answer was 'Yes, I own one gelding' at question 29
-------- or Scenario 18 --------
Answer was 'Yes, I own one gelding' at question 30.
-------- or Scenario 19 --------
Answer was 'Yes, I own one gelding' at question 31.
-------- or Scenario 20 --------
Answer was 'Yes, I own one gelding' at question 32.
Only numbers may be entered in this field.
Please enter your answer here:

If you do not know in which year your equine was gelded, then skip the question.

F 39

In welchem Jahr wurden Ihre Pferde kastriert?
Diese Frage wird nur angezeigt, wenn folgende Bedingungen erfüllt sind:
-------- Scenario 21 --------
Antwort war 'Ja, zwei Wallache' bei Frage '29'
-------- oder Scenario 22 --------
Antwort war 'Ja, ich besitze zwei Wallache' bei Frage '30'
-------- oder Scenario 23 --------
Antwort war 'Ja, ich besitze zwei Wallache' bei Frage '31'
-------- oder Scenario 24 --------
Antwort war 'Ja, ich besitze zwei Wallache' bei Frage '32'
Nur Zahlen dürfen in diese Felder eingegeben werden.
Bitte geben Sie Ihre Antwort(en) hier ein:
Wallach 1: [ ]

Wallach 2: [ ]

Falls Sie nicht wissen, in welchem Jahr Ihre Pferde kastriert wurden, dann überspringen Sie die Frage.

In which year were your equines castrated?
This question is only displayed if the following conditions are met:
-------- Scenario 21 --------
Answer was 'Yes, two geldings' at question 29
-------- or Scenario 22 --------
Answer was 'Yes, I own two geldings' at question 30.
-------- or Scenario 23 --------
Answer was 'Yes, I own two geldings' at question 31.
-------- or Scenario 24 --------
Answer was 'Yes, I own two geldings' at question 32.
Only numbers may be entered in these fields.
Please enter your answer(s) here:
Gelding 1: [ ]

Gelding 2: [ ]

If you do not know what year your equines were gelded, then skip this question.

F 40

In welchem Jahr wurden Ihre Pferde kastriert?
Diese Frage wird nur angezeigt, wenn folgende Bedingungen erfüllt sind:
-------- Scenario 25 --------
Antwort war 'Ja, ich besitze drei Wallache' bei Frage '30'
-------- oder Scenario 26 --------
Antwort war 'Ja, ich besitze drei Wallache' bei Frage '31'
-------- oder Scenario 27 --------
Antwort war 'Ja, ich besitze drei Wallache' bei Frage '32'
Nur Zahlen dürfen in diese Felder eingegeben werden.
Bitte geben Sie Ihre Antwort(en) hier ein:
Wallach 1: [ ]

Wallach 2: [ ]

Wallach 3: [ ]

Falls Sie nicht wissen, in welchem Jahr Ihre Pferde kastriert wurden, dann überspringen Sie die Frage.

In which year were your equines castrated?
This question is only displayed if the following conditions are met:
-------- Scenario 25 --------
Answer was 'Yes, I own three geldings' at question 30.
-------- or Scenario 26 --------
Answer was 'Yes, I own three geldings' at question 31.
-------- or Scenario 27 --------
Answer was 'Yes, I own three geldings' at question 32.
Only numbers may be entered in these fields.
Please enter your answer(s) here:

Gelding 1: [ ]

Gelding 2: [ ]

Gelding 3: [ ]

If you do not know in which year your equines were gelded, then skip this question.

F 41

In welchem Jahr wurden Ihre Pferde kastriert?
Diese Frage wird nur angezeigt, wenn folgende Bedingungen erfüllt sind:
-------- Scenario 28 --------
Antwort war 'Ja, ich besitze vier Wallache' bei Frage '31'
-------- oder Scenario 29 --------
Antwort war 'Ja, ich besitze vier Wallache' bei Frage '32'
Nur Zahlen dürfen in diese Felder eingegeben werden.
Bitte geben Sie Ihre Antwort(en) hier ein:

Wallach 1: [ ]

Wallach 2: [ ]

Wallach 3: [ ]

Wallach 4: [ ]

Falls Sie nicht wissen, in welchem Jahr Ihre Pferde kastriert wurden, dann überspringen Sie die Frage.

In which year were your equines castrated?
This question is only displayed if the following conditions are met:
-------- Scenario 28 --------
Answer was 'Yes, I own four geldings' at question 31
-------- or Scenario 29 --------
Answer was 'Yes, I own four geldings' at question 32.
Only numbers may be entered in these fields.
Please enter your answer(s) here:

Gelding 1: [ ]

Gelding 2: [ ]

Gelding 3: [ ]

Gelding 4: [ ]

If you do not know what year your equines were gelded, then skip this question.

F 42

In welchem Jahr wurden Ihre Pferde kastriert?
Diese Frage wird nur angezeigt, wenn folgende Bedingungen erfüllt sind:
Antwort war 'Ja, ich besitze fünf Wallache' bei Frage '32'
Nur Zahlen dürfen in diese Felder eingegeben werden.
Bitte geben Sie Ihre Antwort(en) hier ein:

Wallach 1: [ ]

Wallach 2: [ ]

Wallach 3: [ ]

Wallach 4: [ ]

Wallach 5: [ ]

Falls Sie nicht wissen, in welchem Jahr Ihre Pferde kastriert wurden, dann überspringen Sie die Frage.

In which year were your equines castrated?
This question is only displayed if the following conditions are met:
Answer was 'Yes, I own five geldings' for question 32.
Only numbers may be entered in these fields.
Please enter your answer(s) here:

Gelding 1: [ ]

Gelding 2: [ ]

Gelding 3: [ ]

Gelding 4: [ ]

Gelding 5: [ ]

If you do not know in which year your equines were gelded, then skip the question.

Arzneimittel / Specialized questions - drugs

F 43

Sind im Abschnitt IX Teil III (Tierarzneimittel) des Pferdepasses Arzneimittel vermerkt? *
Diese Frage wird nur angezeigt, wenn folgende Bedingungen erfüllt sind:
Antwort war '1 Pferd' bei Frage '2'.
Bitte wählen Sie nur eine der folgenden Antworten aus:
- o Ja
- o Nein
- o Weiß ich nicht

Impfungen zählen hierbei nicht als Arzneimittel.

Are any medications documented in Section IX, Part III (Veterinary Medications) of the equine passport? *
This question is only displayed if the following conditions are met:
Answer was '1 equine' for question 2.
Please select only one of the following answers:
- o Yes
- o No
- o I do not know

Vaccinations do not count as medications for this purpose.

F 44

Sind in den Abschnitten IX Teil III (Tierarzneimittel) der Pferdepässe Arzneimittel vermerkt? *
Diese Frage wird nur angezeigt, wenn folgende Bedingungen erfüllt sind:
Antwort war '2 Pferde' bei Frage '2'.
Bitte wählen Sie die zutreffende Antwort für jeden Punkt aus:
Pferd 1:
- o Ja
- o Nein

Pferd 2:
- o Ja
- o Nein

Impfungen zählen hierbei nicht als Arzneimittel.

Are any medications documented in Section IX, Part III (Veterinary Medications) of the equine passports? *
This question is only displayed if the following conditions are met:
Answer was '2 equines' for question 2.
Please select the applicable answer for each item:
Equine 1:
- o Yes
- o No

Equine 2:
- o Yes
- o No

Vaccinations do not count as medications for this purpose.

F 45

Sind in den Abschnitten IX Teil III (Tierarzneimittel) der Pferdepässe Arzneimittel vermerkt? *
Diese Frage wird nur angezeigt, wenn folgende Bedingungen erfüllt sind:
Antwort war '3 Pferde' bei Frage '2'.
Bitte wählen Sie die zutreffende Antwort für jeden Punkt aus:
Pferd 1:
- o Ja
- o Nein

Pferd 2:
- o Ja
- o Nein

Pferd 3:
- o Ja
- o Nein

Impfungen zählen hierbei nicht als Arzneimittel.

Are any medications documented in Section IX, Part III (Veterinary Medications) of the equine passports? *
This question is only displayed if the following conditions are met:
Answer was '3 equines' for question 2.
Please select the applicable answer for each item:
Equine 1:
- o Yes
- o No

Equine 2:
- o Yes
- o No

Equine 3:
- o Yes
- o No

Vaccinations do not count as medications for this purpose.

F 46

Sind in den Abschnitten IX Teil III (Tierarzneimittel) der Pferdepässe Arzneimittel vermerkt? *

Diese Frage wird nur angezeigt, wenn folgende Bedingungen erfüllt sind:
Antwort war '4 Pferde' bei Frage '2'.
Bitte wählen Sie die zutreffende Antwort für jeden Punkt aus:

Pferd 1:
- Ja
- Nein

Pferd 2:
- Ja
- Nein

Pferd 3:
- Ja
- Nein

Pferd 4:
- Ja
- Nein

Impfungen zählen hierbei <u>nicht</u> als Arzneimittel.

Are any medications documented in Section IX, Part III (Veterinary Medications) of the equine passports? *

This question is only displayed if the following conditions are met:
Answer was '4 equines' for question 2.
Please select the applicable answer for each item:

Equine 1:
- Yes
- No

Equine 2:
- Yes
- No

Equine 3:
- Yes
- No

Equine 4:
- Yes
- No

Vaccinations do <u>not</u> count as medications for this purpose.

F 47

Sind in den Abschnitten IX Teil III (Tierarzneimittel) der Pferdepässe Arzneimittel vermerkt? *

Diese Frage wird nur angezeigt, wenn folgende Bedingungen erfüllt sind:
Antwort war '5 oder mehr Pferde' bei Frage '2'.
Bitte wählen Sie die zutreffende Antwort für jeden Punkt aus:

Pferd 1:
- Ja
- Nein

Pferd 2:
- Ja
- Nein

Pferd 3:
- Ja
- Nein

Pferd 4:
- Ja
- Nein

Pferd 5:
- Ja
- Nein

Impfungen zählen hierbei <u>nicht</u> als Arzneimittel.

Are any medications documented in Section IX, Part III (Veterinary Medications) of the equine passports? *

This question is only displayed if the following conditions are met:
Answer was '5 or more equines' for question 2.
Please select the applicable answer for each item:

Equine 1:
- Yes
- No

Equine 2:
- Yes
- No

Equine 3:
- Yes
- No

Equine 4:
- Yes
- No

Equine 5:
- Yes
- No

Vaccinations do <u>not</u> count as medications for this purpose.

F 48

Welche Arzneimittel sind im Pferdepass eingetragen?

Diese Frage wird nur angezeigt, wenn folgende Bedingungen erfüllt sind:

Antwort war 'Ja ' bei Frage '43'. Bitte geben Sie Ihre Antwort hier ein:

Which medicines are documented in the equine passport?

This question is only displayed if the following conditions are met:

Answer was 'Yes ' to question 43. Please enter your answer here:

F 49

Welche Arzneimittel sind im Pferdepass / in den Pferdepässen eingetragen?

Diese Frage wird nur angezeigt, wenn folgende Bedingungen erfüllt sind:

-------- Scenario 30 --------
Antwort war 'Ja' bei Frage '44' (Pferd 1)
-------- oder Scenario 31 --------
Antwort war 'Ja' bei Frage '44' (Pferd 2)
-------- oder Scenario 32 --------
Antwort war 'Ja' bei Frage '45' (Pferd 1)
-------- oder Scenario 33 --------
Antwort war 'Ja' bei Frage '45' (Pferd 2)
-------- oder Scenario 34 --------
Antwort war 'Ja' bei Frage '45' (Pferd 3)
-------- oder Scenario 35 --------
Antwort war 'Ja' bei Frage '46' (Pferd 1)
-------- oder Scenario 36 --------
Antwort war 'Ja' bei Frage '46' (Pferd 2)
-------- oder Scenario 37 --------
Antwort war 'Ja' bei Frage '46' (Pferd 3)
-------- oder Scenario 38 --------
Antwort war 'Ja' bei Frage '46' (Pferd 4)
-------- oder Scenario 39 --------
Antwort war 'Ja' bei Frage '47' (Pferd 1)
-------- oder Scenario 40 --------
Antwort war 'Ja' bei Frage '47' (Pferd 2)
-------- oder Scenario 41 --------
Antwort war 'Ja' bei Frage '47' (Pferd 3)
-------- oder Scenario 42 --------
Antwort war 'Ja' bei Frage '47' (Pferd 4)
-------- oder Scenario 43 --------
Antwort war 'Ja' bei Frage '47' (Pferd 5)

Antwortbeispiel:
Pferd 1: Midazolam, Acepromazin
Pferd 2: -
Pferd 3: Relaquine

Wenn bei einem Pferd keine Medikamente im Equidenpass vermerkt sind, bzw. Sie weniger Pferde besitzen, als Antwortmöglichkeiten gegeben sind, tragen Sie bitte einen Strich in das Antwortfeld ein.

Pferd 1: [ ]

Pferd 2: [ ]

Pferd 3: [ ]

Pferd 4: [ ]

Pferd 5: [ ]

Which medicines are documented in the equine passport(s)?

This question is only displayed if the following conditions are met:

-------- Scenario 30 --------
Answer was 'Yes' to question 44 (Equine 1)
-------- or Scenario 31 --------
Answer was 'Yes' to question 44 (Equine 2)
-------- or Scenario 32 --------
Answer was 'Yes' to question 45 (Equine 1)
-------- or Scenario 33 --------
Answer was 'Yes' to question 45 (Equine 2)
-------- or Scenario 34 --------
Answer was 'Yes' to question 45 (Equine 3)
-------- or Scenario 35 --------
Answer was 'Yes' to question 46 (Equine 1)
-------- or Scenario 36 --------
Answer was 'Yes' to question 46 (Equine 2)
-------- or Scenario 37 --------
Answer was 'Yes' to question 46 (Equine 3)
-------- or Scenario 38 --------
Answer was 'Yes' to question 46 (Equine 4)
-------- or Scenario 39 --------
Answer was 'Yes' to question 47 (Equine 1)
-------- or Scenario 40 --------
Answer was 'Yes' to question 47 (Equine 2)
-------- or Scenario 41 --------
Answer was 'Yes' to question 47 (Equine 3)
-------- or Scenario 42 --------
Answer was 'Yes' to question 47 (Equine 4)
-------- or Scenario 43 --------
Answer was 'Yes' to question 47 (Equine 5)

Sample answer:
Equine 1: Midazolam, Acepromazine.
Equine 2: -
Equine 3: Relaquine

If a horse does not have any medications listed on its equine passport, or you have fewer horses than answer choices given, please enter a dash in the answer box.

Equine 1: [ ]

Equine 2: [ ]

Equine 3: [ ]

Equine 4: [ ]

Equine 5: [ ]

F 50

Werden in dem Stall, in dem Ihr Pferd/Ihre Pferde steht/stehen, gemeinschaftliche Entwurmungen durchgeführt? *

Bitte wählen Sie nur eine der folgenden Antworten aus:

- Ja
- Nein

Does the stable where your horse(s) are located perform community deworming? *

Please select only one of the following responses:

- Yes
- No

F 51

Wie häufig werden gemeinschaftliche Entwurmungen durchgeführt? *

Diese Frage wird nur angezeigt, wenn folgende Bedingungen erfüllt sind:

Antwort war 'Ja' bei Frage '50'.

Bitte wählen Sie nur eine der folgenden Antworten aus:

- Monatlich
- Vierteljährlich
- Halbjährlich
- Jährlich
- Sonstiges

How often are community deworming performed? *

This question is only displayed if the following conditions are met:

Answer was 'Yes' to question 50.

Please select only one of the following responses:

- Monthly
- Quarterly
- Biannually
- Annually
- Other

F 52

Wer wendet die Wurmkur an? *

Diese Frage wird nur angezeigt, wenn folgende Bedingungen erfüllt sind:

Antwort war 'Ja' bei Frage '50'.

Bitte wählen Sie alle zutreffenden Antworten aus:

- Tierarzt/Tierärztin
- Der/die Stallbetreiber*in
- Angestellte/r
- Pferdebesitzer*innen
- Sonstiges:

Falls Sie "Sonstiges" wählen, können Sie dies im Kommentarfeld erläutern.

Who applies the deworming treatment? *

This question is only displayed if the following conditions are met:

Answer was 'Yes' to question 50.

Please select all that apply:

- Veterinarian(s)
- The stable operator
- Employee
- Equine owner
- Other:

If you select "Other", you can explain in the comment field.

F 53

Wissen Sie, ob die Anwendung der Wurmkur dokumentiert wird? *

Diese Frage wird nur angezeigt, wenn folgende Bedingungen erfüllt sind:

Antwort war 'Ja' bei Frage '50'.

Bitte wählen Sie nur eine der folgenden Antworten aus:

- Ja, die Anwendung von Wurmkuren wird dokumentiert
- Nein, die Anwendung von Wurmkuren wird nicht dokumentiert
- Ich weiß nicht, ob die Anwendung von Wurmkuren dokumentiert wird

Do you know if the application of the deworming treatment is documented? *

This question is only displayed if the following conditions are met:

Answer was 'Yes' to question 50.

Please select only one of the following responses:

- Yes, the application of deworming is documented
- No, the application of worming treatments is not documented
- I do not know if the application of deworming is documented

F 54

Hat Ihr Pferd eine oder mehrere chronische Erkrankungen?
Diese Frage wird nur angezeigt, wenn folgende Bedingungen erfüllt sind:
Antwort war '1 Pferd' bei Frage '2'.
Bitte wählen Sie nur eine der folgenden Antworten aus:
- Ja
- Nein

Does your equine have one or more chronic diseases?
This question is only displayed if the following conditions are met:
Answer was '1 equine' for question 2.
Please select only one of the following answers:
- Yes
- No

F 55

Haben Ihre Pferde eine oder mehrere chronische Erkrankungen?
Diese Frage wird nur angezeigt, wenn folgende Bedingungen erfüllt sind:
Antwort war '2 Pferde' bei Frage '2'.
Bitte wählen Sie die zutreffende Antwort für jeden Punkt aus:
Pferd 1:
- Ja
- Nein

Pferd 2:
- Ja
- Nein

Do your equines have one or more chronic diseases?
This question is only displayed if the following conditions are met:
Answer was '2 equines' for question 2.
Please select the applicable answer for each item:
Equine 1:
- Yes
- No

Equine 2:
- Yes
- No

F 56

Haben Ihre Pferde eine oder mehrere chronische Erkrankungen?
Diese Frage wird nur angezeigt, wenn folgende Bedingungen erfüllt sind:
Antwort war '3 Pferde' bei Frage '2'.
Bitte wählen Sie die zutreffende Antwort für jeden Punkt aus:
Pferd 1:
- Ja
- Nein

Pferd 2:
- Ja
- Nein

Pferd 3:
- Ja
- Nein

Do your equines have one or more chronic diseases?
This question is only displayed if the following conditions are met:
Answer was '3 equines' for question 2.
Please select the applicable answer for each item:
Equine 1:
- Yes
- No

Equine 2:
- Yes
- No

Equine 3:
- Yes
- No

F 57

Haben Ihre Pferde eine oder mehrere chronische Erkrankungen?

Diese Frage wird nur angezeigt, wenn folgende Bedingungen erfüllt sind:

Antwort war '4 Pferde' bei Frage '2'.

Bitte wählen Sie die zutreffende Antwort für jeden Punkt aus:

Pferd 1:
- o Ja
- o Nein

Pferd 2:
- o Ja
- o Nein

Pferd 3:
- o Ja
- o Nein

Pferd 4:
- o Ja
- o Nein

Do your equines have one or more chronic diseases?

This question is only displayed if the following conditions are met:

Answer was '4 equines' for question 2.

Please select the applicable answer for each item:

Equine 1:
- o Yes
- o No

Equine 2:
- o Yes
- o No

Equine 3:
- o Yes
- o No

Equine 4:
- o Yes
- o No

F 58

Haben Ihre Pferde eine oder mehrere chronische Erkrankungen?

Diese Frage wird nur angezeigt, wenn folgende Bedingungen erfüllt sind:

Antwort war '5 oder mehr Pferde' bei Frage '2'.

Bitte wählen Sie die zutreffende Antwort für jeden Punkt aus:

Pferd 1:
- o Ja
- o Nein

Pferd 2:
- o Ja
- o Nein

Pferd 3:
- o Ja
- o Nein

Pferd 4:
- o Ja
- o Nein

Pferd 5:
- o Ja
- o Nein

Do your equines have one or more chronic diseases?

This question is only displayed if the following conditions are met:

Answer was '5 or more equines' for question 2.

Please select the applicable answer for each item:

Equine 1:
- o Yes
- o No

Equine 2:
- o Yes
- o No

Equine 3:
- o Yes
- o No

Equine 4:
- o Yes
- o No

Equine 5:
- o Yes
- o No

F 59

Welche Erkrankungen hat Ihr Pferd?

Diese Frage wird nur angezeigt, wenn folgende Bedingungen erfüllt sind:

Antwort war 'Ja' bei Frage '54'. Bitte geben Sie Ihre Antwort hier ein:

[ ]

What diseases does your equine have?

This question is only displayed if the following conditions are met:

Answer was 'Yes' to question 54. Please enter your answer here:

[ ]

F 60

Welche Erkrankung hat Ihr Pferd / haben Ihre Pferde?
Diese Frage wird nur angezeigt, wenn folgende Bedingungen erfüllt sind:
-------- Scenario 44 --------
Antwort war 'Ja' bei Frage '55' (Pferd 1)
-------- oder Scenario 45 --------
Antwort war 'Ja' bei Frage '55 (Pferd 2)
-------- oder Scenario 46 --------
Antwort war 'Ja' bei Frage '56' (Pferd 1)
-------- oder Scenario 47 --------
Antwort war 'Ja' bei Frage '56' (Pferd 2)
-------- oder Scenario 48 --------
Antwort war 'Ja' bei Frage '56' (Pferd 3)
-------- oder Scenario 49 --------
Antwort war 'Ja' bei Frage '57' (Pferd 1)
-------- oder Scenario 50 --------
Antwort war 'Ja' bei Frage '57' (Pferd 2)
-------- oder Scenario 51 --------
Antwort war 'Ja' bei Frage '57' (Pferd 3)
-------- oder Scenario 52 --------
Antwort war 'Ja' bei Frage '57' (Pferd 4)
-------- oder Scenario 53 --------
Antwort war 'Ja' bei Frage '58' (Pferd 1)
-------- oder Scenario 54 --------
Antwort war 'Ja' bei Frage '58' (Pferd 2)
-------- oder Scenario 55 --------
Antwort war 'Ja' bei Frage '58' (Pferd 3)
-------- oder Scenario 56 --------
Antwort war 'Ja' bei Frage '58' (Pferd 4)
-------- oder Scenario 57 --------
Antwort war 'Ja' bei Frage '58' (Pferd 5)

Beispielantwort:
Pferd 1: COB
Pferd 2: -
Pferd 3: Cushing
Wenn bei einem Pferd keine chronische Erkrankung vorliegt, bzw. Sie weniger Pferde besitzen, als Antwortmöglichkeiten gegeben sind, tragen Sie bitte einen Strich in das Antwortfeld ein.

Pferd 1: [ ]

Pferd 2: [ ]

Pferd 3: [ ]

Pferd 4: [ ]

Pferd 5: [ ]

What disease does your equine/s have?
This question is only displayed if the following conditions are met:
-------- Scenario 44 --------
Answer was 'Yes' to question 55 (Equine 1)
-------- or Scenario 45 --------
Answer was 'Yes' to question 55 (Equine 2)
-------- or Scenario 46 --------
Answer was 'Yes' to question 56 (Equine 1)
-------- or Scenario 47 --------
Answer was 'Yes' to question 56 (Equine 2)
-------- or Scenario 48 --------
Answer was 'Yes' to question 56 (Equine 3)
-------- or Scenario 49 --------
Answer was 'Yes' to question 57 (Equine 1)
-------- or Scenario 50 --------
Answer was 'Yes' to question 57 (Equine 2)
-------- or Scenario 51 --------
Answer was 'Yes' to question 57 (Equine 3)
-------- or Scenario 52 --------
Answer was 'Yes' to question 57 (Equine 4)
-------- or Scenario 53 --------
Answer was 'Yes' to question 58 (Equine 1)
-------- or Scenario 54 --------
Answer was 'Yes' to question 58 (Equine 2)
-------- or Scenario 55 --------
Answer was 'Yes' to question 58 (Equine 3)
-------- or Scenario 56 --------
Answer was 'Yes' to question 58 (Equine 4)
-------- or Scenario 57 --------
Answer was 'Yes' to question 58 (Equine 5)

Example answer:
Equine 1: COB
Equine 2: -
Equine 3: Cushing's
If a horse does not have a chronic condition, or you have fewer horses than answer choices given, please enter a dash in the answer box.

Equine 1: [ ]

Equine 2: [ ]

Equine 3: [ ]

Equine 4: [ ]

Equine 5: [ ]

F 61

Bekommt das Pferd für die Erkrankungen regelmäßig Medikamente?
Diese Frage wird nur angezeigt, wenn folgende Bedingungen erfüllt sind:
Antwort war 'Ja' bei Frage '54'
Bitte wählen Sie nur eine der folgenden Antworten aus:

- Ja
- Nein

Is the equine receiving regular medication for the conditions?
This question is only displayed if the following conditions are met:
Answer was 'Yes' to question 54.
Please select only one of the following answers:

- Yes
- No

F 62

Bekommen die Pferde für die Erkrankungen regelmäßig Medikamente?
Diese Frage wird nur angezeigt, wenn folgende Bedingungen erfüllt sind:
-------- Scenario 44 --------
Antwort war 'Ja' bei Frage '55' (Pferd 1)
-------- oder Scenario 45 --------
Antwort war 'Ja' bei Frage '55' (Pferd 2)
-------- oder Scenario 46 --------
Antwort war 'Ja' bei Frage '56' (Pferd 1)
-------- oder Scenario 47 --------
Antwort war 'Ja' bei Frage '56' (Pferd 2)
-------- oder Scenario 48 --------
Antwort war 'Ja' bei Frage '56' (Pferd 3)
-------- oder Scenario 49 --------
Antwort war 'Ja' bei Frage '57' (Pferd 1)
-------- oder Scenario 50 --------
Antwort war 'Ja' bei Frage '57' (Pferd 2)
-------- oder Scenario 51 --------
Antwort war 'Ja' bei Frage '57' (Pferd 3)
-------- oder Scenario 52 --------
Antwort war 'Ja' bei Frage '57' (Pferd 4)
-------- oder Scenario 53 --------
Antwort war 'Ja' bei Frage '58' (Pferd 1)
-------- oder Scenario 54 --------
Antwort war 'Ja' bei Frage '58' (Pferd 2)
-------- oder Scenario 55 --------
Antwort war 'Ja' bei Frage '58' (Pferd 3)
-------- oder Scenario 56 --------
Antwort war 'Ja' bei Frage '58' (Pferd 4)
-------- oder Scenario 57 --------
Antwort war 'Ja' bei Frage '58' (Pferd 5)
Bitte wählen Sie nur eine der folgenden Antworten aus:

- Ja
- Teilweise
- Nein

Do the equines receive regular medication for the conditions?
This question is only displayed if the following conditions are met:
-------- Scenario 44 --------
Answer was 'Yes' to question 55 (Equine 1)
-------- or Scenario 45 --------
Answer was 'Yes' to question 55 (Equine 2)
-------- or Scenario 46 --------
Answer was 'Yes' to question 56 (Equine 1)
-------- or Scenario 47 --------
Answer was 'Yes' to question 56 (Equine 2)
-------- or Scenario 48 --------
Answer was 'Yes' to question 56 (Equine 3)
-------- or Scenario 49 --------
Answer was 'Yes' to question 57 (Equine 1)
-------- or Scenario 50 --------
Answer was 'Yes' to question 57 (Equine 2)
-------- or Scenario 51 --------
Answer was 'Yes' to question 57 (Equine 3)
-------- or Scenario 52 --------
Answer was 'Yes' to question 57 (Equine 4)
-------- or Scenario 53 --------
Answer was 'Yes' to question 58 (Equine 1)
-------- or Scenario 54 --------
Answer was 'Yes' to question 58 (Equine 2)
-------- or Scenario 55 --------
Answer was 'Yes' to question 58 (Equine 3)
-------- or Scenario 56 --------
Answer was 'Yes' to question 58 (Equine 4)
-------- or Scenario 57 --------
Answer was 'Yes' to question 58 (Equine 5)
Please select only one of the following answers:

- Yes
- Partially
- No

F 63

Welche Medikamente bekommt das Pferd / die Pferde?
Diese Frage wird nur angezeigt, wenn folgende Bedingungen erfüllt sind:
-------- Scenario 1 --------
Antwort war 'Teilweise' *oder* 'Ja' bei Frage '62'
-------- oder Scenario 50 --------
Antwort war 'Ja' bei Frage '61'
Beispielantwort:
Pferd 1: Budesonid
Wenn bei einem Pferd keine Medikamente im Equidenpass vermerkt sind, bzw. Sie weniger Pferde besitzen, als Antwortmöglichkeiten gegeben sind, tragen Sie bitte einen Strich in das Antwortfeld ein.
Pferd 1: [ ]

Pferd 2: [ ]

Pferd 3: [ ]

Pferd 4: [ ]

Pferd 5: [ ]

What medication is/are the equine(s) receiving?
This question is only displayed if the following conditions are met:
-------- Scenario 1 --------
Answer was 'Partially' or 'Yes' to question 62
-------- or Scenario 50 --------
Answer was 'Yes' to question 61
Example response:
Equine 1: Budesonide
If an equine does not have any medications listed on its equine passport, or you have fewer horses than answer choices given, please enter a dash in the answer box.
Equine 1: [ ]

Equine 2: [ ]

Equine 3: [ ]

Equine 4: [ ]

Equine 5: [ ]

F 64

Aus welchen Quellen beziehen Sie Medikamente für Ihre Pferde? *

Bitte wählen Sie alle zutreffenden Antworten aus:
- Tierarzt/Tierärztin
- Apotheker*in
- Tierheilpraktiker*in
- Hufschmied*in
- Chiropraktiker*in
- Aus dem Internet
- Sonstiges: [ ]

Als Medikamente gelten auch z.B. Wundpflegemittel wie Zink- oder Jodsalbe, Wurmkuren, Schmerzmittel, Augentropfen wie Euphrasia, Antibiotika
Falls Sie "Sonstiges" wählen, können Sie dies im Kommentarfeld erläutern.

From which sources do you obtain medication for your equine/s? *

Please select all that apply:
- Veterinarian
- Pharmacist
- Animal healer
- Farrier
- Chiropractor
- From the Internet
- Other: [ ]

Medication also includes, for example, wound care products such as zinc or iodine ointment, deworming treatments, painkillers, eye drops such as Euphrasia, antibiotics.
If you select "Other", you can explain this in the comment field.

F 65

Beziehen Sie Medikamente von einem/einer im Ausland ansässigen Tierarzt/Tierärztin? *

Bitte wählen Sie nur eine der folgenden Antworten aus:

- o Ja, alle Medikamente
- o Ja, aber nur manche Medikamente
- o Nein

Do you obtain medications from a veterinarian(s) located abroad? *

Please select only one of the following answers:

- o Yes, all medications
- o Yes, but only some medications
- o No

F 66

In welchem Land ist Ihr Tierarzt / Ihre Tierärztin ansässig? *

Diese Frage wird nur angezeigt, wenn folgende Bedingungen erfüllt sind:
Antwort war 'Ja, aber nur manche Medikamente' *oder* 'Ja, alle Medikamente' bei Frage '65'.
Bitte geben Sie Ihre Antwort hier ein:

In which country is your veterinarian/veterinarian located? *

This question is only displayed if the following conditions are met:
Answer was 'Yes, but only some medications' or 'Yes, all medications' for question 65.
Please enter your answer here:

Allgemeine Fragen / General specialized questions

F 67

Wann ist Ihrer Meinung nach ein Pferd ein Schlachtpferd? *

Bitte wählen Sie nur eine der folgenden Antworten aus:

- o Wenn es im Pferdepass eingetragen wird
- o Jedes Pferd ist ein Schlachtpferd bis es im Pferdepass ausgetragen wird
- o In Deutschland gibt es keine Schlachtpferde
- o Weiß ich nicht
- o Sonstiges: ☐

Falls Sie "Sonstiges" wählen, können Sie dies im Kommentarfeld erläutern.

Under which circumstances is an equine considered for slaughter? *

Please select only one of the following answers:

- o It is considered for slaughter after it is classified as such in the equine passport
- o Every equine is considered for slaughter until its status is changed in the equine passport.
- o There are no slaughter equines in Germany
- o I do not know
- o Other: ☐

If you select "Other", you can explain this in the comment field.

F 68

Ist Ihr Pferd laut Pferdepass ein Schlachttier? *

Diese Frage wird nur angezeigt, wenn folgende Bedingungen erfüllt sind:
Antwort war '1 Pferd' bei Frage '2'.
Bitte wählen Sie nur eine der folgenden Antworten aus:

- o Ja
- o Nein
- o Weiß ich nicht

Does your equine have the status of a slaughter equine and is therefore considered for human consumption? *

This question is only displayed if the following conditions are met:
Answer was '1 equine' for question 2.
Please select only one of the following answers:

- o Yes
- o No
- o I do not know

F 69

Sind Ihre Pferde laut Pferdepass Schlachttiere? *

Diese Frage wird nur angezeigt, wenn folgende Bedingungen erfüllt sind:
Antwort war '2 Pferde' bei Frage '2'.
Bitte wählen Sie die zutreffende Antwort für jeden Punkt aus:

Pferd 1:

- o Ja
- o Nein
- o Weiß ich nicht

Pferd 2:

- o Ja
- o Nein
- o Weiß ich nicht

Do your equines have the status of a slaughter equine and are therefore considered for human consumption? *

This question is only displayed if the following conditions are met:
Answer was '2 equines' for question 2.
Please select the applicable answer for each item:

Equine 1:

- o Yes
- o No
- o I do not know

Equine 2:

- o Yes
- o No
- o I do not know

F 70

Sind Ihre Pferde laut Pferdepass Schlachttiere? *
Diese Frage wird nur angezeigt, wenn folgende Bedingungen erfüllt sind:
Antwort war '3 Pferde' bei Frage '2'.
Bitte wählen Sie die zutreffende Antwort für jeden Punkt aus:
Pferd 1:
- o Ja
- o Nein
- o Weiß ich nicht

Pferd 2:
- o Ja
- o Nein
- o Weiß ich nicht

Pferd 3:
- o Ja
- o Nein
- o Weiß ich nicht

Do your equines have the status of a slaughter equine and are therefore considered for human consumption? *
This question is only displayed if the following conditions are met:
Answer was '3 equines' for question 2.
Please select the applicable answer for each item:
Equine 1:
- o Yes
- o No
- o I do not know

Equine 2:
- o Yes
- o No
- o I do not know

Equine 3:
- o Yes
- o No
- o I do not know

F 71

Sind Ihre Pferde laut Pferdepass Schlachttiere? *
Diese Frage wird nur angezeigt, wenn folgende Bedingungen erfüllt sind:
Antwort war '4 Pferde' bei Frage '2'.
Bitte wählen Sie die zutreffende Antwort für jeden Punkt aus:
Pferd 1:
- o Ja
- o Nein
- o Weiß ich nicht

Pferd 2:
- o Ja
- o Nein
- o Weiß ich nicht

Pferd 3:
- o Ja
- o Nein
- o Weiß ich nicht

Pferd 4:
- o Ja
- o Nein
- o Weiß ich nicht

Do your equines have the status of a slaughter equine and are therefore considered for human consumption? *
This question is only displayed if the following conditions are met:
Answer was '4 equines' for question 2.
Please select the applicable answer for each item:
Equine 1:
- o Yes
- o No
- o I do not know

Equine 2:
- o Yes
- o No
- o I do not know

Equine 3:
- o Yes
- o No
- o I do not know

Equine 4:
- o Yes
- o No
- o I do not know

F 72

Sind Ihre Pferde laut Pferdepass Schlachttiere? *

Diese Frage wird nur angezeigt, wenn folgende Bedingungen erfüllt sind:
Antwort war '5 oder mehr Pferde' bei Frage '2'.
Bitte wählen Sie die zutreffende Antwort für jeden Punkt aus:

Pferd 1:
- Ja
- Nein
- Weiß ich nicht

Pferd 2:
- Ja
- Nein
- Weiß ich nicht

Pferd 3:
- Ja
- Nein
- Weiß ich nicht

Pferd 4:
- Ja
- Nein
- Weiß ich nicht

Pferd 5:
- Ja
- Nein
- Weiß ich nicht

Do your equines have the status of a slaughter equine and are therefore considered for human consumption? *

This question is only displayed if the following conditions are met:
Answer was '5 or more equines' for question 2.
Please select the applicable answer for each item:

Equine 1:
- Yes
- No
- I do not know

Equine 2:
- Yes
- No
- I do not know

Equine 3:
- Yes
- No
- I do not know

Equine 4:
- Yes
- No
- I do not know

Equine 5:
- Yes
- No
- I do not know

F 73

Würden Sie Ihr Pferd/Ihre Pferde zum Schlachten geben?

Bitte wählen Sie nur eine der folgenden Antworten aus:
- Ja
- Vielleicht
- Nein

Would you hypothetically hand in your equine(s) for slaughter?

Please select only one of the following responses:
- Yes
- Maybe
- No

F 74

In welchem Jahr wurde Ihr Pferd / Ihre Pferde im Pferdepass als Schlachttier ausgetragen? *
Diese Frage wird nur angezeigt, wenn folgende Bedingungen erfüllt sind:
-------- Scenario 1 --------
Antwort war 'Nein' bei Frage '68'
-------- oder Scenario 2 --------
Antwort war 'Nein' bei Frage '69' (Pferd 1)
-------- oder Scenario 3 --------
Antwort war 'Nein' bei Frage '69' (Pferd 2)
-------- oder Scenario 4 --------
Antwort war 'Nein' bei Frage '70' (Pferd 1)
-------- oder Scenario 5 --------
Antwort war 'Nein' bei Frage '70' (Pferd 2)
-------- oder Scenario 6 --------
Antwort war 'Nein' bei Frage '70' (Pferd 3)
-------- oder Scenario 7 --------
Antwort war 'Nein' bei Frage '71' (Pferd 1)
-------- oder Scenario 8 --------
Antwort war 'Nein' bei Frage '71' (Pferd 2)
-------- oder Scenario 9 --------
Antwort war 'Nein' bei Frage '71' (Pferd 3)
-------- oder Scenario 10 --------
Antwort war 'Nein' bei Frage '71' (Pferd 4)
-------- oder Scenario 11 --------
Antwort war 'Nein' bei Frage '72' (Pferd 1)
-------- oder Scenario 12 --------
Antwort war 'Nein' bei Frage '72' (Pferd 2)
-------- oder Scenario 13 --------
Antwort war 'Nein' bei Frage '72' (Pferd 3)
-------- oder Scenario 14 --------
Antwort war 'Nein' bei Frage '72' (Pferd 4)
-------- oder Scenario 15 --------
Antwort war 'Nein' bei Frage '72' (Pferd 5)

Bitte geben Sie Ihre Antwort hier ein:
Pferd 1: [ ]

Pferd 2: [ ]

Pferd 3: [ ]

Pferd 4: [ ]

Pferd 5: [ ]
Antwortbeispiel:
Pferd 1: 2015
Pferd 2: 2018
Pferd 3: Kein Datum lesbar

In what year was/were your equine(s) documented as a slaughter equine in the equine passport? *
This question is only displayed if the following conditions are met:
-------- Scenario 1 --------
Answer was 'No' to question 68
-------- or Scenario 2 --------
Answer was 'No' to question 69 (Equine 1)
-------- or Scenario 3 --------
Answer was 'No' to question 69 (Equine 2)
-------- or Scenario 4 --------
Answer was 'No' to question 70 (Equine 1)
-------- or Scenario 5 --------
Answer was 'No' to question 70 (Equine 2)
-------- or Scenario 6 --------
Answer was 'No' to question 70 (Equine 3)
-------- or Scenario 7 --------
Answer was 'No' to question 71 (Equine 1)
-------- or Scenario 8 --------
Answer was 'No' to question 71 (Equine 2)
-------- or Scenario 9 --------
Answer was 'No' to question 71 (Equine 3)
-------- or Scenario 10 --------
Answer was 'No' to question 71 (Equine 4)
-------- or Scenario 11 --------
Answer was 'No' to question 72 (Equine 1)
-------- or Scenario 12 --------
Answer was 'No' to question 72 (Equine 2)
-------- or Scenario 13 --------
Answer was 'No' to question 72 (Equine 3)
-------- or Scenario 14 --------
Answer was 'No' to question 72 (Equine 4)
-------- or Scenario 15 --------
Answer was 'No' to question 72 (Equine 5)

Please enter your answer here:
Equine 1: [ ]

Equine 2: [ ]

Equine 3: [ ]

Equine 4: [ ]

Equine 5:
Sample answer:
Equine 1: 2015
Equine 2: 2018
Equine 3: No date readable

Pferdepass / Specialized questions – Equine passport

F 75

Wo befindet sich Ihr Pferdepass / wo befinden sich Ihre Pferdepässe? *
Bitte wählen Sie alle zutreffenden Antworten aus:

- Bei mir zu Hause
- Im Pferdehänger
- Im Stall
- Bei dem/der Tierarzt/Tierärztin
- Weiß ich nicht
- Sonstiges: ____

Falls Sie "Sonstiges" wählen, können Sie dies im Kommentarfeld erläutern.

Where is/are your equine passport(s)? *
Please select all that apply:

- At my home
- In the horse trailer
- In the stable
- At the veterinarian's office
- I do not know
- Other: ____

If you select "Other," you can explain in the comment box.

F 76

Wie oft lässt sich der Tierarzt / die Tierärztin den Pferdepass / die Pferdepässe zeigen? *
Bitte wählen Sie nur eine der folgenden Antworten aus:

- Nie
- Bei Erstvorstellung des Pferdes
- Je nach Behandlung
- Vor jeder Behandlung
- Weiß ich nicht
- Sonstiges: ____

Falls Sie "Sonstiges" wählen, können Sie dies im Kommentarfeld erläutern.

How frequently does your attending veterinarian inspect the equine passport/s? *
Please select only one of the following answers:

- Never
- During first admission of the equine
- Depending on the type of treatment
- Before every treatment
- I do not know
- Other: ____

If you select "Other", you can explain in the comment field.

F 77

Wissen Sie, was Arzneimittelanwendungs- und Abgabebelege (*AuA-Belege) sind? *
Bitte wählen Sie nur eine der folgenden Antworten aus:

- Ja
- Nein

Do you know what drug application and dispersing forms (*AuA-Belege) are? *
Please select only one of the following answers:

- Yes
- No

F 78

Gibt der Tierarzt / die Tierärztin AuA-Belege an Sie ab? (z.B. bei Abgabe von Wurmkuren) *
Diese Frage wird nur angezeigt, wenn folgende Bedingungen erfüllt sind:
Antwort war 'Ja' bei Frage '77'.

Bitte wählen Sie nur eine der folgenden Antworten aus:
- Ja, immer
- Ja, manchmal
- Nein

AuA-Belege sind Papiere, auf den die Anwendung, oder die Abgabe von Medikamenten vermerkt ist. Rechnungen sind <u>keine</u> AuA-Belege.

Do you receive drug application and dispersion forms ('AuA-Belege') from your attending veterinarians, for example in the scope of receiving anthelmintic treatment for your equine? *.
This question is only displayed if the following conditions are met:
Answer was 'Yes' to question 77.

Please select only one of the following answers:
- Yes, always
- Yes, sometimes
- No

AuA-Belege receipts are papers on which the use, or dispensing, of medications is noted. Invoices are <u>not</u> AuA-Belege receipts.

F 79

Was tun Sie mit den AuA-Belegen, die Ihnen der Tierarzt / die Tierärztin gibt? *
Diese Frage wird nur angezeigt, wenn folgende Bedingungen erfüllt sind:
Antwort war 'Ja, immer' *oder* 'Ja, manchmal' bei Frage '78'.

Bitte wählen Sie nur eine der folgenden Antworten aus:
- Ich hebe die AuA-Belege ein Jahr auf.
- Ich hebe die AuA-Belege fünf Jahre auf.
- Ich hebe die AuA-Belege so lange auf, wie sich das Pferd in meinem Besitz befindet.
- Ich gebe die AuA-Belege dem/der Betreiber*in des Stalles, in dem mein Pferd steht.
- Ich werfe die AuA-Belege weg.
- Sonstiges: [ ]

Falls Sie "Sonstiges" wählen, können Sie dies im Kommentarfeld erläutern.

What do you do with the drug application and dispersion forms ('AuA-Belege') that you receive from your attending veterinarian? *
This question is only displayed if the following conditions are met:
Answer was 'Yes, always' or 'Yes, sometimes' to question 78.

Please select only one of the following answers:
- I keep the AuA-forms for one year.
- I keep the AuA-forms for five years.
- I keep the AuA- forms as long as the equine is in my possession.
- I give the AuA-forms to the operator of the stable where my equine is kept.
- I throw away the AuA-forms.
- Other: [ ]

If you choose "Other", you can explain this in the comment field.

Feedback und Anmerkungen / Feedback and comments

Falls Sie Anmerkungen zum Fragebogen oder Ihren Antworten haben, können Sie diese in das Textfeld eintragen.
Bitte geben Sie Ihre Antwort hier ein:

If you have any comments about the questionnaire or your answers, you can enter them in the text box.
Please enter your answer here:

Vielen Dank für Ihre Teilnahme!

Thank you for your participation!

Postanschrift:
Freien Universität Berlin
Fachbereich Veterinärmedizin
Institut für Lebensmittelsicherheit und –hygiene
AG Fleischhygiene
Königsweg 67, Gebäude 21/22
14163 Berlin

E-Mail:
schneides91@zedat.fu-berlin.de

Postal address:
Freie Universität Berlin
Department of Veterinary Medicine
Institute for Food Safety and Hygiene
WG Meat Hygiene
Königsweg 67, Building 21/22
14163 Berlin

Email:
schneides91@zedat.fu-berlin.de

Übermittlung Ihres ausgefüllten Fragebogens: Vielen Dank für die Beantwortung des Fragebogens.

Transmission of your completed questionnaire: Thank you very much for answering the questionnaire.

* Pflichtfrage / Mandatory question
