# Supplementary material for: A comparative survey of veterinarians, equine owners, and equine keepers regarding the knowledge and implementation of legal requirements in Germany for the use and documentation of veterinary medicines in equines intended for slaughter
Source: PLoS One. 2023 Apr 6;18(4):e0283371. doi: 10.1371/journal.pone.0283371 (PMC10079036; doi:10.1371/journal.pone.0283371)
Supplement: S3 File — (PDF) [file pone.0283371.s012.pdf]

S3 File – Questionnaire for equine keepers

Tierarzneimitteldokumentation bei Equiden - Fragebogen für Stallbetreiber*innen

Vielen Dank für Ihr Interesse und Ihre Unterstützung für das Forschungsvorhaben „Arzneimittelanwendung und Dokumentation bei Equiden“.
Mein Name ist Shary Schneider, ich bin Tierärztin und schreibe eine Doktorarbeit an der Freien Universität Berlin am Fachbereich Veterinärmedizin.
Im Rahmen meiner Doktorarbeit befrage ich Tierbesitzer*innen, Stallbetreiber*innen und Tierärzte/Tierärztinnen zu verschiedenen Punkten der Arzneimittelanwendung bei Pferden und Eseln.
Ziel dieser Befragung ist es, den Stand der aktuell bei Equiden praktizierten Tiermedizin abzubilden. So sollen langfristig Ansatzpunkte zur Verbesserung der Arzneimittelsicherheit bei Pferden und Eseln gefunden werden.
Der nachfolgende Fragebogen für Stallbetreiber*innen und Pferdehalter*innen umfasst allgemeine Fragen, Fragen zur Behandlung von Pferden und Eseln, Anwendungen von Arzneimitteln, sowie Arzneimitteldokumentation.
Die Dauer der Befragung beträgt ca. 15 Minuten.
Sämtliche Daten werden nach den Richtlinien der DSGVO (Datenschutz-Grundverordnung) streng vertraulich behandelt. Eine personenbezogene Darstellung, sowie die Weitergabe an Dritte sind grundsätzlich ausgeschlossen. Dieses Forschungsprojekt steht in keinerlei Verbindung zur behördlichen Überwachung.
Vielen Dank, dass Sie sich Zeit nehmen, um an dieser Studie teilzunehmen!
In dieser Umfrage sind 42 Fragen enthalten.

Veterinary drug documentation for equines - Questionnaire for equine keepers (i.e., stable operators)

Thank you for your interest and support for the research project "Medication use and documentation in equines".
My name is Shary Schneider, I am a veterinarian and I am writing a doctoral thesis at the Freie Universität Berlin in the Department of Veterinary Medicine.
As part of my doctoral thesis, I am interviewing animal owners, stable operators, and veterinarians about various aspects of drug use in horses and donkeys.
The aim of this survey is to map the state of veterinary medicine currently practiced for equids. In this way, starting points for improving drug safety in horses and donkeys will be found in the long term.
The following questionnaire for stable operators and horse owners includes general questions, questions about the treatment of horses and donkeys, applications of medicines, and drug documentation.
The duration of the questionnaire is about 15 minutes.
All data will be treated as strictly confidential according to the guidelines of the DSGVO (Data Protection Regulation). Any personal representation or passing any data on to third parties are principally excluded. This research project is in no way connected to official monitoring.
Thank you for taking the time to participate in this study!
There are 42 questions in this survey.

Demographische Fragen / Demographic questions

F 1

In welchem Bundesland befindet sich Ihr Betrieb? *

Bitte wählen Sie nur eine der folgenden Antworten aus:

- o Baden-Württemberg
- o Bayern
- o Berlin
- o Brandenburg
- o Bremen
- o Hamburg
- o Hessen
- o Mecklenburg-Vorpommern
- o Niedersachsen
- o Nordrhein-Westfalen
- o Rheinland-Pfalz
- o Saarland
- o Sachsen
- o Sachsen-Anhalt
- o Schleswig-Holstein
- o Thüringen

In which federal state is your stable located? *

Please select only one of the following answers:

- o Baden-Wuerttemberg
- o Bavaria
- o Berlin
- o Brandenburg
- o Bremen
- o Hamburg
- o Hessia
- o Mecklenburg-Western Pomerania
- o Lower Saxony
- o North Rhine-Westphalia
- o Rhineland-Palatinate
- o Saarland
- o Saxony
- o Saxony-Anhalt
- o Schleswig-Holstein
- o Thuringia

F 2

Was für einen Pferde- oder Esel-haltenden Betrieb betreiben Sie? *

Bitte wählen Sie alle zutreffenden Antworten aus:

- o Pensionsbetrieb
- o Zuchtbetrieb
- o Reitschule
- o Stutenmilchbetrieb
- o Sonstiges: [ ]

Falls Sie "Sonstiges" wählen, können Sie dies im Kommentarfeld erläutern.

What kind of horse- or donkey-keeping stable do you operate? *

Please select all that apply:

- o Boarding stable
- o Breeding stable
- o Horse-riding school
- o Mare milk farm
- o Other: [ ]

If you select "Other", you can explain in the comment field.

F 3

Wie viele Pferde stehen ungefähr auf Ihrem Betrieb? *

Bitte wählen Sie nur eine der folgenden Antworten aus:

- o <10 Pferde
- o 10 bis <20 Pferde
- o 20 bis <50 Pferde
- o 50 bis 100 Pferde
- o Mehr als 100 Pferde
- o Ich halte nur Esel

How many horses do you keep? *

Please select only one of the following answers:

- o Less than 10 horses
- o From 10 to less than 20 horses
- o From 20 to less than 50 horses
- o From 50 to 100 horses
- o More than 100 horses
- o I only keep donkeys

F 4

Wie viel Prozent der bei Ihnen eingestallten Pferde sind Schlachtpferde? *

Diese Frage wird nur angezeigt, wenn folgende Bedingungen erfüllt sind:
Antwort war '<10 Pferde' oder '10 bis <20 Pferde' oder '20 bis <50 Pferde' oder '50 bis 100 Pferde' oder 'Mehr als 100 Pferde' bei Frage '3'.
Bitte wählen Sie nur eine der folgenden Antworten aus:

- <10%
- 10% bis <25%
- 25% bis <50%
- 50% bis <75%
- 75% bis ≤100%
- Ist mir nicht bekannt

What percentage of the kept horses is considered for slaughter? *

This question is only displayed if the following conditions are met:
Answer was '<10 horses' or '10 to <20 horses' or '20 to <50 horses' or '50 to 100 horses' or 'More than 100 horses' for question 3.
Please select only one of the following responses:

- <10%
- 10% to <25
- 25% to <50%
- 50% to <75%
- 75% to ≤100%
- Not known to me

F 5

Wie häufig werden neue Pferde durchschnittlich in Ihrem Betrieb pro Jahr eingestallt? *

Beantworten Sie diese Frage nur, wenn folgende Bedingungen erfüllt sind:
Antwort war '<10 Pferde' oder '10 bis <20 Pferde' oder '20 bis <50 Pferde' oder '50 bis 100 Pferde' oder 'Mehr als 100 Pferde' bei Frage '3'.
In dieses Feld dürfen nur Zahlen eingegeben werden.
Bitte geben Sie Ihre Antwort hier ein:

On average, how often are new horses stabled on your farm per year? *

Answer this question only if the following conditions are met:
Answer was '<10 horses' or '10 to <20 horses' or '20 to <50 horses' or '50 to 100 horses' or 'More than 100 horses' for question 3.
Only numbers may be entered in this field.
Please enter your answer here:

F 6

Züchten Sie Pferde? *

Diese Frage wird nur angezeigt, wenn folgende Bedingungen erfüllt sind:
Antwort war bei Frage '2' und Antwort war '<10 Pferde' oder 'Mehr als 100 Pferde' oder '50 bis 100 Pferde' oder '20 bis <50 Pferde' oder '10 bis <20 Pferde' bei Frage '3'.
Bitte wählen Sie nur eine der folgenden Antworten aus:

- Ja
- Nein

Do you breed horses? *

This question is only displayed if the following conditions are met:
Answer was '<10 horses' for question '2' and Answer was 'More than 100 horses' or '50 to 100 horses' or '20 to <50 horses' or '10 to <20 horses' for question 3.
Please select only one of the following answers:

- Yes
- No

F 7

Wie viele Fohlen werden im Durchschnitt jährlich auf Ihrem Betrieb geboren? *
Diese Frage wird nur angezeigt, wenn folgende Bedingungen erfüllt sind:
Antwort war 'Ja' bei Frage '6'.
In dieses Feld dürfen nur Zahlen eingegeben werden.
Bitte geben Sie Ihre Antwort hier ein:

On average, how many foals are born annually on your farm? *
This question is only displayed if the following conditions are met:
Answer was 'Yes' to question 6.
Only numbers may be entered in this field.
Please enter your answer here:

F 8

Wurde schon einmal ein Pferd auf Ihrem Betrieb notgeschlachtet?
Diese Frage wird nur angezeigt, wenn folgende Bedingungen erfüllt sind:
Antwort war '<10 Pferde' oder '10 bis <20 Pferde' oder '20 bis <50 Pferde' oder '50 bis 100 Pferde' oder 'Mehr als 100 Pferde' bei Frage '3'.
Bitte wählen Sie nur eine der folgenden Antworten aus:

- Ja
- Nein
- Weiß ich nicht

Was it ever necessary to perform an emergency slaughter of a horse in your stable?
This question is only displayed if the following conditions are met:
Answer was '<10 horses' or '10 to <20 horses' or '20 to <50 horses' or '50 to 100 horses' or 'More than 100 horses' for question 3.
Please select only one of the following responses:

- Yes
- No
- I do not know

F 9

Wie viele Esel stehen auf Ihrem Betrieb? *
Bitte wählen Sie nur eine der folgenden Antworten aus:

- <10 Esel
- 10 bis <20 Esel
- 20 bis <50 Esel
- 50 bis 100 Esel
- Mehr als 100 Esel
- Ich halte nur Pferde

How many donkeys do you keep? *
Please select only one of the following answers:

- Less than 10 donkeys
- From 10 to less than 20 donkeys
- From 20 to less than 50 donkeys
- From 50 to 100 donkeys
- More than 100 donkeys
- I only keep horses

F 10

Wie viel Prozent der bei Ihnen eingestallten Esel sind Schlachttiere? *
Diese Frage wird nur angezeigt, wenn folgende Bedingungen erfüllt sind:
Antwort war '<10 Esel' oder '10 bis <20 Esel' oder '20 bis <50 Esel' oder '50 bis 100 Esel' oder 'Mehr als 100 Esel' bei Frage '9'.
Bitte wählen Sie nur eine der folgenden Antworten aus:

- <10%
- 10% bis <25%
- 25% bis <50%
- 50% bis <75%
- 75% bis ≤100%
- Ist mir nicht bekannt

What percentage of the kept donkeys is considered for slaughter? *
This question is only displayed if the following conditions are met:
Answer was '<10 donkeys' or '10 to <20 donkeys' or '20 to <50 donkeys' or '50 to 100 donkeys' or 'More than 100 donkeys' for question 9.
Please select only one of the following answers:

- <10%
- 10% to <25
- 25% to <50
- 50% to <75%
- 75% to ≤100%
- Not known to me

F 11

Wie häufig werden neue Esel durchschnittlich in Ihrem Betrieb pro Jahr eingestallt? *
Diese Frage wird nur angezeigt, wenn folgende Bedingungen erfüllt sind:
Antwort war 'Mehr als 100 Esel' oder '50 bis 100 Esel' oder '20 bis <50 Esel' oder '10 bis <20 Esel' oder '<10 Esel' bei Frage '9'.
In dieses Feld dürfen nur Zahlen eingegeben werden.
Bitte geben Sie Ihre Antwort hier ein:

On average, how often are new donkeys stabled on your farm per year? *
This question is only displayed if the following conditions are met:
Answer was 'More than 100 donkeys' or '50 to 100 donkeys' or '20 to <50 donkeys' or '10 to <20 donkeys' or '<10 donkeys' for question 9.
Only numbers may be entered in this field.
Please enter your answer here:

F 12

Züchten Sie Esel? *
Diese Frage wird nur angezeigt, wenn folgende Bedingungen erfüllt sind:
Antwort war bei Frage '2' und Antwort war '<10 Esel' oder '10 bis <20 Esel' oder '20 bis <50 Esel' oder '50 bis 100 Esel' oder 'Mehr als 100 Esel' bei Frage '9'.
Bitte wählen Sie nur eine der folgenden Antworten aus:

- Ja
- Nein

Do you breed donkeys? *
This question is only displayed if the following conditions are met:
Answer was '<10 donkeys' or '10 to <20 donkeys' or '20 to <50 donkeys' or '50 to 100 donkeys' or 'More than 100 donkeys' at question '2' and answer was '<10 donkeys' or '10 to <20 donkeys' or '20 to <50 donkeys' or '50 to 100 donkeys' at question 9.
Please select only one of the following answers:

- Yes
- No

F 13

Wie viele Eselfohlen werden im Durchschnitt jährlich auf Ihrem Betrieb geboren? *
Diese Frage wird nur angezeigt, wenn folgende Bedingungen erfüllt sind:
Antwort war 'Ja' bei Frage '12'.
In dieses Feld dürfen nur Zahlen eingegeben werden.
Bitte geben Sie Ihre Antwort hier ein:

On average, how many donkey foals are born on your farm each year? *
This question is only displayed if the following conditions are met:
Answer was 'Yes' to question 12.
Only numbers may be entered in this field.
Please enter your answer here:

F 14

Wurde schon einmal ein Esel auf Ihrem Betrieb notgeschlachtet?
Diese Frage wird nur angezeigt, wenn folgende Bedingungen erfüllt sind:
Antwort war '<10 Esel' oder '10 bis <20 Esel' oder '20 bis <50 Esel' oder '50 bis 100 Esel' oder 'Mehr als 100 Esel' bei Frage '9'.
Bitte wählen Sie nur eine der folgenden Antworten aus:

- Ja
- Nein
- Weiß ich nicht

Was it ever necessary to perform an emergency slaughter of a donkey in your stable?
This question is only displayed if the following conditions are met:
Answer was '<10 donkeys' or '10 to <20 donkeys' or '20 to <50 donkeys' or '50 to 100 donkeys' or 'More than 100 donkeys' for question 9.
Please select only one of the following answers:

- Yes
- No
- I do not know

F 15

Wie viele verschiedene Tierärzte/Tierärztinnen betreuen Tiere auf Ihrem Betrieb? *

In dieses Feld dürfen nur Zahlen eingegeben werden.

Bitte geben Sie Ihre Antwort hier ein:

How many veterinarians attend to animals in your stable? *

Only numbers may be entered in this field.

Please enter your answer here:

Behandlung und Anwendung von Arzneimitteln / Treatment and application of drugs – spezific questions

F 16

Werden Entwurmungen bei Ihnen im Betrieb nach einem einheitlichen, vorgegebenen Schema durchgeführt? *

Bitte wählen Sie eine der folgenden Antworten:
Bitte wählen Sie nur eine der folgenden Antworten aus:

- Ja, alle Tiere werden regelmäßig, gemeinsam entwurmt.
- Ja, es werden gemeinsame Entwurmungen durchgeführt, aber nicht bei allen Tieren gleichzeitig.
- Nein, es werden keine gemeinsamen Entwurmungen durchgeführt.

Are deworming treatments carried out in your stable according to a uniform, predefined scheme? *

Please select one of the following answers:
Please select only one of the following answers:

- Yes, all animals are dewormed regularly, together.
- Yes, common deworming is performed, but not on all animals at the same time.
- No, no common deworming tratments are performed.

F 17

Wie häufig werden in Ihrem Betrieb gemeinsame Entwurmungen durchgeführt? *

Diese Frage wird nur angezeigt, wenn folgende Bedingungen erfüllt sind:
Antwort war 'Ja, alle Tiere werden regelmäßig, gemeinsam entwurmt.' oder 'Ja, es werden gemeinsame Entwurmungen durchgeführt, aber nicht bei allen Tieren gleichzeitig.' bei Frage '16'.
Bitte wählen Sie nur eine der folgenden Antworten aus:

- Monatlich
- Vierteljährlich
- Halbjährlich
- Jährlich
- Sonstiges: ____

Falls Sie "Sonstiges" wählen, können Sie dies im Kommentarfeld erläutern.

How often are common deworming treatments performed in your stable? *

This question is only displayed if the following conditions are met:
Answer was 'Yes, all animals are dewormed regularly, together.' or 'Yes, joint deworming is performed, but not on all animals at the same time.' for question 16. Please select only one of the following answers:

- Monthly
- Quarterly
- Biannually
- Annually
- Other: ____

If you select "Other", you can explain in the comment field.

F 18

Aus welchen Quellen werden Medikamente für die bei Ihnen eingestallten Tiere bezogen? *

Bitte wählen Sie alle zutreffenden Antworten aus:

- Tierarzt/Tierärztin
- Apotheker*in
- Tierheilpraktiker*in
- Hufschmied*in
- Chiropraktiker*in
- Aus dem Internet
- Sonstiges: ____

Als Medikamente gelten auch z.B. Wurmkuren, Wundpflegemittel wie Zink- oder Jodsalbe,

From which sources do you obtain medication for your equine/s? *

Please select all that apply:

- Veterinarian
- Pharmacist
- Animal healer
- Farrier
- Chiropractor
- From the Internet
- Other: ____

Medication also includes e.g., deworming treatments, wound care products such as zinc or iodine ointment, painkillers, eye drops such as Euphrasia, antibiotics

Schmerzmittel, Augentropfen wie Euphrasia, Antibiotika

F 19

Beziehen Sie Medikamente von einem im Ausland ansässigen Tierarzt/Tierärztin? *
Bitte wählen Sie nur eine der folgenden Antworten aus:

- o Ja, alle Medikamente
- o Ja, aber nur manche Medikamente
- o Nein

Do you obtain medications from a veterinarian located abroad? *
Please select only one of the following answers:

- o Yes, all medications
- o Yes, but only some medications
- o No

F 20

Welche Personen wenden in Ihrem Betrieb Medikamente (z.B. Wurmkuren) an? *
Bitte wählen Sie alle zutreffenden Antworten aus:

- o Tierarzt/Tierärztin
- o Pferdebesitzer*in
- o Stallbetreiber*in
- o Angestellte/r
- o Sonstiges: [ ]

Which people apply the medications (e.g., deworming) on your farm? *
Please select all that apply:

- o Veterinarian
- o Horse owner
- o Stable operator
- o Employee
- o Other: [ ]

F 21

Wird die Anwendung von Medikamenten (z.B. Wurmkuren) durch Sie, Ihr Personal, Tierarzt / Tierärztin oder Tierbesitzer*in dokumentiert? *
Bitte wählen Sie nur eine der folgenden Antworten aus:

- o Ja
- o Manchmal
- o Nein
- o Weiß ich nicht

Is the administration of drugs documented either by yourself, the attending veterinarian, the equine owner, or by your staff? *
Please select only one of the following answers:

- o Yes
- o Sometimes
- o No
- o I do not know

F 22

Von wem wird die Anwendung dokumentiert? *
Diese Frage wird nur angezeigt, wenn folgende Bedingungen erfüllt sind:
Antwort war 'Ja' oder 'Manchmal' bei Frage '21'.
Bitte wählen Sie alle zutreffenden Antworten aus:

- o Derjenige, der das Medikament anwendet
- o Tierbesitzer*in
- o Stallbetreiber*in
- o Personal
- o Tierarzt/Tierärztin
- o Sonstiges: [ ]

By whom is the application documented? *
This question is only displayed if the following conditions are met:
Answer was 'Yes' or 'Sometimes' for question 21.
Please select all that apply:

- o The person who uses the medication
- o Animal owner
- o Stable operator
- o Employee
- o Veterinarian
- o Other: [ ]

F 23

Werden Medikamente auf Ihrem Betrieb gelagert? *
Bitte wählen Sie nur eine der folgenden Antworten aus:
- o Ja
- o Nein
- o Weiß ich nicht

Are medications stored on your farm? *
Please select only one of the following answers:
- o Yes
- o No
- o I do not know

F 24

Wo werden Medikamente für die Tiere auf Ihrem Betrieb gelagert? *
Diese Frage wird nur angezeigt, wenn folgende Bedingungen erfüllt sind:
Antwort war 'Ja' bei Frage '23'.
Bitte wählen Sie alle zutreffenden Antworten aus:
- o Jede/r Pferdebesitzer*in lagert seine eigenen Medikamente
- o In der Sattelkammer
- o Im Stallbüro
- o Im Wohngebäude
- o Sonstiges: [ ]

Falls Sie "Sonstiges" wählen, können Sie dies im Kommentarfeld erläutern.

Where are medications for the animals stored on your farm? *
This question is only displayed if the following conditions are met:
Answer was 'Yes' to question 23.
Please select all that apply:
- o Each horse owner* stores his/her own medications.
- o In the tack room
- o In the stable office
- o In the living quarters
- o Other: [ ]

If you select "Other", you can explain in the comment box.

F 25

Wer hat alles Zugriff auf die Medikamente für die Tiere auf Ihrem Betrieb? *
Diese Frage wird nur angezeigt, wenn folgende Bedingungen erfüllt sind:
Antwort war 'Ja' bei Frage '23'.
Bitte wählen Sie nur eine der folgenden Antworten aus:
- o Nur Stallbetreiber*in
- o Nur Tierbesitzer*in
- o Nur Angestellte/r
- o Stallbetreiber*in und angestellte Personen
- o Stallbetreiber*in, angestellte Personen und jeweilige/r Tierbesitzer*in
- o Sonstiges: [ ]

Who has access to the medications for the animals on your farm? *
This question is only displayed if the following conditions are met:
Answer was 'Yes' to question 23.
Please select only one of the following answers:
- o Stable operator*only
- o Animal owner only
- o Employee only
- o Stable owner* and employed persons
- o Stable owner, employed persons, and respective animal owner
- o Other: [ ]

F 26

Werden Medikamente zwischen den Pferdebesitzer*innen / Eselbesitzer*innen geteilt? *
Bitte wählen Sie nur eine der folgenden Antworten aus:
- o Ja
- o Nein
- o Weiß ich nicht

Are medications shared between horse owners/donkey owners? *
Please select only one of the following answers:
- o Yes
- o No
- o I do not know

F 27

Werden alternative Medikamente angewandt? *

Bitte wählen Sie alle zutreffenden Antworten aus:

- Homöopathika
- Phytotherapeutika (Medikamente aus Heilpflanzen)
- Chinesische Heilpilze
- MMS (Miracle Mineral Supplement)
- CDS/CDL (Chlordioxid)
- EM (effektive Mikroorgansimen)
- Weiß ich nicht
- Nein
- Sonstiges: [ ]

Falls Sie "Sonstiges" wählen, können Sie dies im Kommentarfeld erläutern.

Are alternative medications used? *

Please select all that apply:

- Homeopathics
- Phytotherapeutics (medicines from medicinal plants)
- Chinese medicinal mushrooms
- MMS (Miracle Mineral Supplement)
- CDS/CDL (chlorine dioxide)
- EM (effective microorganisms)
- Do not know
- No
- Other: [ ]

If you select "Other", you can explain in the comment box.

F 28

Verwenden Sie, bzw. werden Pferde / Esel auf Ihrem Hof mit Canabidiol- (CBD, Stoff der Hanfpflanze) haltigen Futterergänzungsmitteln gefüttert?

Bitte wählen Sie nur eine der folgenden Antworten aus:

- Ja
- Nein
- Weiß ich nicht

Do you use or are horses / donkeys on your farm fed with feed supplements containing canabidiol substances (CBD, a substance of the hemp plant)?

Please select only one of the following answers:

- Yes
- No
- I do not know

Dokumentation / Documentation – specific questions

F 29

Sehen Sie den Equidenpass vor Einstallung eines neuen Tieres ein? *
Bitte wählen Sie die zutreffende Antwort für jeden Punkt aus:
- Immer
- Meistens
- Gelegentlich
- Selten
- Nie

How frequently do you inspect the equine passport before boarding a new equine in your stable? *
Please select the applicable answer for each item:
- Always
- Most of the time
- Occasionally
- Rarely
- Never

F 30

Wo befinden sich die Equidenpässe? *
Bitte wählen Sie alle zutreffenden Antworten aus:
- Bei dem/der Tierbesitzer*in
- Im Betrieb
- Beim Tierarzt/ bei der Tierärztin
- Das ist von Tier zu Tier unterschiedlich
- Weiß ich nicht
- Sonstiges: [ ]

Falls Sie "Sonstiges" wählen, können Sie dies im Kommentarfeld erläutern.

Where are the equine passports located? *
Please select all that apply:
- With the animal owner
- At the farm
- At the veterinarian's office
- It varies from animal to animal
- I do not know
- Other: [ ]

If you select "Other," you can explain in the comment box.

F 31

Wie oft lässt sich der Tierarzt / die Tierärztin den Equidenpass zeigen?
Bitte wählen Sie nur eine der folgenden Antworten aus:
- Nur bei Erstvorstellung des Pferdes
- Ab und zu, je nach Behandlung
- Vor jeder Behandlung
- Weiß ich nicht
- Nie

How frequently does your attending veterinarian inspect the equine passport/s?
Please select only one of the following answers:
- Only when the horse is first seen
- From time to time, depending on the treatment
- Before each treatment
- I do not know
- Never

F 32

Wann ist Ihrer Meinung nach ein Pferd ein Schlachtpferd, bzw. ein Esel ein Schlachtesel? *
Bitte wählen Sie nur eine der folgenden Antworten aus:
- Wenn es im Equidenpass eingetragen wird
- Jedes Pferd / jeder Esel ist ein Schlachttier, bis es im Equidenpass ausgetragen wird
- In Deutschland gibt es keine Schlachtpferde / Schlachtesel
- Weiß ich nicht

Under which circumstances is an equine considered for slaughter? *
Please select only one of the following answers:
- When it is registered in the equine passport.
- Every horse/donkey is a slaughter animal until it is entered in the equine passport
- There are no horses / donkeys for slaughter in Germany
- I do not know

F 33

Ist bei jedem auf Ihrem Betrieb stehenden Pferd / Esel, das / der nicht geschlachtet werden soll, dies auch im Equidenpass eingetragen? *

Bitte wählen Sie nur eine der folgenden Antworten aus:

- o Ja, bei jedem Tier
- o Ja, bei den meisten Tieren
- o Nein
- o Weiß ich nicht

Is the status of companion animal documented in the equines´ passports for every equine in your stable that is not destined for slaughter? *

Please select only one of the following answers:

- o Yes, for every animal
- o Yes, for most animals
- o No
- o I do not know

F 34

Kennen Sie die Vorgaben der Tierhalter-Arzneimittelanwendungs- und Nachweis-Verordnung? *

Bitte wählen Sie die zutreffende Antwort für jeden Punkt aus:

Ich kenne die Vorgaben der Tierhalter-Arzneimittelanwendungs- und Nachweis-Verordnung:

- o Sehr gut
- o Gut
- o Teilweise
- o Wenig
- o Gar nicht

How well do you know the documentation requirements of drug usage in equines (THAMNV 2015)? *

Please select the correct answer for each item:

I know the requirements of the Ordinance on the Use and Certification of Medicinal Products for Animal Husbandry:

- o Very well
- o Well
- o Moderate
- o Poor
- o Not at all

F 35

Wissen Sie, was Arzneimittelanwendungs- und Abgabebelege 'AuA-Belege') sind? ***

Bitte wählen Sie nur eine der folgenden Antworten aus:

- o Ja
- o Nein

**AuA-Belege sind Papiere, auf denen die Anwendung/Abgabe von Medikamenten vermerkt ist.

Do you know what drug application and dispersion forms ('AuA-Belege') are? ***

Please select only one of the following responses:

- o Yes
- o No

**AuA-Belege are papers that indicate the use/dispensing of medications.

F 36

Gibt der Tierarzt / die Tierärztin AuA-Belege an Sie oder Ihr Personal ab (z.B. bei Abgabe von Wurmkuren)? *

Diese Frage wird nur angezeigt, wenn folgende Bedingungen erfüllt sind:

Antwort war 'Ja' bei Frage '35'.

Bitte wählen Sie nur eine der folgenden Antworten aus:

- o Ja, immer
- o Ja, manchmal
- o Nein

Rechnungen sind <u>keine</u> AuA-Belege.

Do you receive drug application and dispersion forms ('AuA-Belege') from your attending veterinarians, for example in the scope of receiving anthelmintic treatment for equines you keep? *

This question is only displayed if the following conditions are met:

Answer was 'Yes' to question 35.

Please select only one of the following answers:

- o Yes, always
- o Yes, sometimes
- o No

Invoices are <u>not</u> AuA receipts.

F 37

Werden die AuA-Belege auf Ihrem Betrieb aufbewahrt? *
Diese Frage wird nur angezeigt, wenn folgende Bedingungen erfüllt sind:
Antwort war 'Ja, immer' oder 'Ja, manchmal' bei Frage '36'.
Bitte wählen Sie nur eine der folgenden Antworten aus:

- Ja, immer
- Ja, manchmal
- Nein

Rechnungen sind <u>keine</u> AuA-Belege.

Do you store the drug application and dispersion forms '(AuA-Belege') which you receive from the veterinarian/s? *
This question is only displayed if the following conditions are met:
Answer was 'Yes, always' or 'Yes, sometimes' to question 36.
Please select only one of the following answers:

- Yes, always
- Yes, sometimes
- No

Invoices are <u>not</u> AuA-Belege.

F 38

Wie lange werden die AuA-Belege auf Ihrem Betrieb aufbewahrt? *
Diese Frage wird nur angezeigt, wenn folgende Bedingungen erfüllt sind:
Antwort war 'Ja, immer' oder 'Ja, manchmal' bei Frage '37'.
Bitte wählen Sie nur eine der folgenden Antworten aus:

- Ein Jahr
- Drei Jahre
- Fünf Jahre
- Solange das Pferd in meinem Stall steht
- Solange, bis ich sie dem Pferdebesitzer überreichen kann

Rechnungen sind <u>keine</u> AuA-Belege.

How long do you store the drug application and dispersion forms ('AuA-Belege')? *
This question is only displayed if the following conditions are met:
Answer was 'Yes, always' or 'Yes, sometimes' for question 37.
Please select only one of the following answers:

- One year
- Three years
- Five years
- As long as the horse is in my stable
- Until I can give it to the horse owner.

Invoices are <u>not</u> AuA-Belege.

F 39

Wer ist bei Ihnen für die Aufbewahrung der AuA- Belege zuständig? *
Diese Frage wird nur angezeigt, wenn folgende Bedingungen erfüllt sind:
Antwort war 'Ja, immer' oder 'Ja, manchmal' bei Frage '37'.
Bitte wählen Sie nur eine der folgenden Antworten aus:

- Der/die Stallbetreiber*in
- Die Person, die die Belege erhält
- Ein Angestellter / eine Angestellte
- Sonstiges: _____

Falls Sie "Sonstiges" auswählen, können Sie dies im Kommentarfeld erläutern.

Who is responsible for the storage of the drug application and dispersion forms? *
This question is only displayed if the following conditions are met:
Answer was 'Yes, always' or 'Yes, sometimes' to question 37.
Please select only one of the following answers:

- The stable operator*.
- The person who receives the form
- An employee / an employee
- Other: _____

If you select "Other", you can explain in the comment field.

F 40

Dokumentieren Sie die Anwendungen von Arzneimitteln, z.B. in Form eines Stallbuches? *

Bitte wählen Sie nur eine der folgenden Antworten aus:

- Ja, immer
- Ja, meistens
- Ja, unregelmäßig
- Nein

Is the administration of drugs documented by yourself? *

Please select only one of the following answers:

- Yes, always
- Yes, most of the time
- Yes, irregularly
- No

F 41

Wäre für Sie eine Vorlage, wie ein Stallbuch zur Dokumentation von Arzneimittelanwendungen zu führen ist, hilfreich? *

Bitte wählen Sie nur eine der folgenden Antworten aus:

- Ja
- Nein
- Gleichgültig

Would you perceive a template on how to document drug administrations as helpful? *

Please select only one of the following responses:

- Yes
- No
- Indifferent

Feedback und Anmerkungen / Feedback and comments

F 42

Falls Sie Anmerkungen zum Fragebogen oder Ihren Antworten haben, können Sie diese in das Textfeld eintragen.
Bitte geben Sie Ihre Antwort hier ein:

If you have any comments about the questionnaire or your answers, you can enter them in the text box.
Please enter your answer here:

Vielen Dank für Ihre Teilnahme!

Thank you for your participation!

Postanschrift:
Freien Universität Berlin
Fachbereich Veterinärmedizin
Institut für Lebensmittelsicherheit und –hygiene
AG Fleischhygiene
Königsweg 67, Gebäude 21/22
14163 Berlin

E-Mail:
schneides91@zedat.fu-berlin.de

Postal address:
Freie Universität Berlin
Department of Veterinary Medicine
Institute for Food Safety and Hygiene
WG Meat Hygiene
Königsweg 67, Building 21/22
14163 Berlin

Email:
schneides91@zedat.fu-berlin.de

Übermittlung Ihres ausgefüllten Fragebogens: Vielen Dank für die Beantwortung des Fragebogens.

Transmission of your completed questionnaire: Thank you very much for answering the questionnaire.

* Pflichtfrage / Mandatory question
